# Quantum Chern–Simons Theories on Cylinders: BV-BFV Partition Functions

**DOI:** 10.1007/s00220-022-04513-8

**Published:** 2022-12-05

**Authors:** Alberto S. Cattaneo, Pavel Mnev, Konstantin Wernli

**Affiliations:** 1 Institut für Mathematik, Universität Zürich, Winterthurerstrasse 190, 8057 Zürich, Switzerland; 2 University of Notre Dame, Notre Dame, Indiana 46556 USA; 3 St. Petersburg Department of V. A. Steklov Institute of Mathematics of the Russian Academy of Sciences, Fontanka 27, 191023 St. Petersburg, Russia; 4 Centre for Quantum Mathematics, IMADA, University of Southern Denmark, Odense, Denmark

## Abstract

We compute partition functions of Chern–Simons type theories for cylindrical spacetimes $I \times \Sigma$, with *I* an interval and $\dim \Sigma = 4l+2$, in the BV-BFV formalism (a refinement of the Batalin–Vilkovisky formalism adapted to manifolds with boundary and cutting–gluing). The case $\dim \Sigma = 0$ is considered as a toy example. We show that one can identify—for certain choices of residual fields—the “physical part” (restriction to degree zero fields) of the BV-BFV effective action with the Hamilton–Jacobi action computed in the companion paper (Cattaneo et al., Constrained systems, generalized Hamilton–Jacobi actions, and quantization, arXiv:2012.13270), without any quantum corrections. This Hamilton–Jacobi action is the action functional of a conformal field theory on $\Sigma$. For $\dim \Sigma = 2$, this implies a version of the CS-WZW correspondence. For $\dim \Sigma = 6$, using a particular polarization on one end of the cylinder, the Chern–Simons partition function is related to Kodaira–Spencer gravity (a.k.a. BCOV theory); this provides a BV-BFV quantum perspective on the semiclassical result by Gerasimov and Shatashvili.

## Introduction

This paper is a sequel to the paper “Constrained systems, generalized Hamilton–Jacobi actions, and quantization” [14] by the same authors (but can be read independently).

As announced in [14], the main result of this paper is the explicit computation of the perturbative partition functions of Chern–Simons theories on cylinders $I \times \Sigma$, with respect to various boundary polarizations. Their restriction to degree zero fields turns out to be the exponential of the corresponding Hamilton–Jacobi action, defined in [14] and recalled in Sect. 2, without any quantum corrections.

Interestingly, the Hamilton–Jacobi actions of the theories we consider can be related to action functionals of conformal field theories on $\Sigma$. This means that the partition function of Chern–Simons theories (with certain boundary conditions) can be identified with the partition function of a conformal field theory (coupled to sources)—a property that one might call “holographic duality”. In that terminology, among other results, we show the following:

- The holographic dual theory of 3D abelian Chern–Simons theory is the 2D free boson CFT, see Sect. 1.2.1 (while for a different choice of boundary polarization, we obtain the beta-gamma system as the dual, see (24)).
- The holographic dual of 3D nonabelian Chern–Simons theory is WZW theory (see Sect. 1.2.2). In particular, the bulk-boundary version of the Batalin–Vilkovisky master equation (referred to below as the modified quantum master equation) corresponds to the Polyakov–Wiegmann formula for the WZW action functional.
- The holographic dual of 7D Chern–Simons theory is a free 2-form theory for the “standard” polarization and the Kodaira–Spencer gravity for a particular nonlinear polarization (see Sect. 1.2.3).

A remark on the terminology: The term “holographic duality” is often used for the case where the bulk theory is a theory of quantum gravity, e.g., a string theory, such as in the celebrated AdS/CFT correspondence [30, 39] and its more general variant, the gauge/gravity correspondence (see [16] for a review). The bulk/boundary correspondences we discuss below were, in a different context, discovered earlier, see for instance [24, 37]. They can be interpreted as special cases of holography, thinking of Chern–Simons theory as a string theory [40].

The first motivating point for this paper and its prequel [14], suggested to us by Shatashvili, concerned precisely the last item in the list above: namely, the systematical understanding of the relation between 7D abelian Chern–Simons theory and 6D Kodaira–Spencer [29] gravity (otherwise known as BCOV theory [8]) from the BV-BFV perspective. At the semiclassical level, the relation is a result of Gerasimov–Shatashvili [24] (see also our review in [14, Section 7.6]). In this paper, we explore the perturbative BV-BFV quantization and show that, for an appropriate choice of gauge fixing, no quantum corrections are added to the semiclassical result. We thus prove the conjecture put forward by Gerasimov and Shatashvili in their original paper.

There are two other key points that motivated this paper and its companion paper [14]. Firstly, we were interested in studying in detail the bulk-boundary or “holographic” correspondences mentioned above. In this paper we prove that in these special cases the boundary theory is simply an effective theory of the bulk theory, in the sense that the bulk fields have been partially integrated out. Given the results in [14], we expect that the effective action viewpoint can explain more general bulk-boundary correspondences. To put it in clear words: holographic duality means that the boundary theory is the semiclassical limit of a certain effective action of the bulk theory. In the theories we consider in this paper, this semiclassical limit is exact,^1^ but in general there is of course no reason to expect this.

Secondly, partition functions on cylinders can be interpreted as kernels of generalized Segal–Bargmann transforms (see Appendix A). They are of interest because, in a *d*-dimensional theory, they describe how a state on a $(d-1)$-dimensional manifold $\Sigma$ depends on the choice of a polarization. One way to interpret our results is that in our examples those generalized Segal–Bargmann transforms (in general, it is only their semiclassical limit) can be described by another quantum field theory that lives on $\Sigma$.

Our results show that in both those cases – seemingly unrelated at first glance – the corresponding boundary theory is given by a (generalized) Hamilton–Jacobi action. The BV-BFV formalism turns out to be a clear conceptual framework in which one can state and prove those results from first principles: the only inputs required are those of a local field theory, namely a space of fields $F_M$ and an action functional $S_M :F_M \rightarrow \mathbb {R}$. From a different perspective, this paper can also be viewed as an invitation to learn the formalism.

Before passing to a detailed description of our results, as a primer on the BV-BFV formalism we give a brief recollection of abelian Chern–Simons theory in the BV-BFV formalism, which can be safely skipped by readers familiar with the subject.

### Chern–Simons theory in the BV-BFV formalism

We consider abelian Chern–Simons theory in dimensions $d = 4l + 3$ with *l* a positive integer. For a *d*-dimensional spacetime manifold *N* (possibly with boundary), the space of fields is defined as $F_N = \Omega ^{2l+1}(N)$ and the action functional is

$$\begin{aligned} S_N[A] = \frac{1}{2} \int _N A \wedge d A. \end{aligned}$$

In dimension $d=3$, we also consider nonabelian Chern–Simons theory. Here there is a structure Lie algebra $\mathcal {G}$ of coefficients endowed with a nondegenerate invariant pairing $\langle \cdot , \cdot \rangle$. The space of fields on *N* is then the space of $\mathcal {G}$-valued 1-forms $F_N = \Omega ^{1}(N,\mathcal {G})$ (thought of as the space of connections on a trivial principal *G*-bundle $N\times G$ with *G* the connected and simply connected Lie group integrating $\mathcal {G}$). The action functional is

$$\begin{aligned} S_N[A] = \int _N \frac{1}{2} \langle A, dA \rangle + \frac{1}{6} \langle A, [A,A]\rangle . \end{aligned}$$

Since these theories are gauge theories, to define the perturbative partition function we need a gauge fixing formalism. In this paper, we will use the BV-BFV formalism, the modification of the Batalin–Vilkovisky (BV) formalism for manifolds with boundary introduced by two of the authors together with Reshetikhin [10, 13]. Let us briefly explain this formalism by means of our main example.

The BV-BFV extension of abelian Chern–Simons theory has $\mathbb {Z}$-graded space of fields $\mathcal {F}_N = \Omega ^\bullet (N)[2l+1]$. This notation is shorthand for saying that a homogeneous form $\omega$ is assigned ghost number $\textrm{gh}(\omega ) = 2l+1 - \deg (\omega )$, so that all forms have *total degree* $\textrm{gh}+ \deg = 2l+1$. In particular $\mathcal {F}^0_N = F_N$. The space $\mathcal {F}_N$ is an odd symplectic vector space with odd symplectic form

$$\begin{aligned} \omega _N(\mathcal {A},\mathcal {A}') = \int _N \mathcal {A}\wedge \mathcal {A}', \end{aligned}$$

where $\mathcal {A},\mathcal {A}'$ are nonhomogeneous differential forms and only the top degree part contributes to the integral.^2^ The BV extended action functional of abelian Chern–Simons theory is

$$\begin{aligned} \mathcal {S}_N[\mathcal {A}] = \frac{1}{2}\int _N \mathcal {A}\wedge d\mathcal {A}. \end{aligned}$$

In particular, restricting to forms of ghost number 0, we recover the classical action $S_N[A]$.

If $\partial N = \emptyset$, then $(\mathcal {S}_N,\mathcal {S}_N) = 0$, where $(\cdot ,\cdot )$ denotes the Poisson bracket induced by $\omega _N$. This equation is called classical master equation in the BV formalism, and it implies $Q_N^2 = 0$, where

$$\begin{aligned} Q_N = \int _N d\mathcal {A}\wedge \frac{\delta }{\delta \mathcal {A}} \end{aligned}$$

is the odd hamiltonian vector field of $\mathcal {S}_N$.

If $\partial N \ne \emptyset$, then we assign additional BFV^3^ data to the boundary. The space of boundary fields is $\mathcal {F}^\partial _{\partial N}= \Omega ^\bullet (\partial N)[2l+1]$ with even symplectic form

$$\begin{aligned} \omega ^\partial _{\partial N}(\mathcal {A},\mathcal {A}') = \int _{\partial N} \mathcal {A}\wedge \mathcal {A}'. \end{aligned}$$

This symplectic form is the de Rham differential (on $\mathcal {F}^\partial _{\partial N}$) of the 1-form

$$\begin{aligned} \alpha ^\partial _{\partial N} = \frac{1}{2} \int _{\partial N} \mathcal {A}\wedge \delta \mathcal {A}. \end{aligned}$$

Finally, using the surjective submersion $\pi :\mathcal {F}_N \rightarrow \mathcal {F}^\partial _{\partial N}$, given by pullback of differential forms from *N* to $\partial N$, we can project the vector field^4^$Q_N$ to $\mathcal {F}^\partial _{\partial N}$. One can check that it is also hamiltonian. For degree reasons it then automatically has a unique odd hamiltonian function that we denote by $\mathcal {S}^\partial _{\partial N}$. The important structural relation between the boundary BFV data $(\mathcal {F}^\partial _{\partial N},\alpha ^\partial _{\partial N}, \mathcal {S}^\partial _{\partial N})$ and the bulk “broken” BV data $(\mathcal {F}_N,\omega _N,\mathcal {S}_N,Q_N,\pi )$ is

1 $$\begin{aligned} \delta \mathcal {S}_N =\iota _{Q_N}\omega _N + \pi ^*\alpha ^\partial _{\partial N} . \end{aligned}$$

The data, together with the structural relation (1), are the content of the classical BV-BFV formalism. For more details we refer to Cattaneo et al. [10].

For *f* a function on $\mathcal {F}^\partial _{\partial N}$, there is a symmetry of the data given by shifting $\mathcal {S}_N \rightarrow \mathcal {S}_N^f =\mathcal {S}_N + \pi ^*f$ and $\alpha ^\partial _{\partial N} \rightarrow \alpha ^{\partial , f}_{\partial N} = \alpha ^\partial _{\partial N} + \delta f$. Clearly this is a symmetry of Eq. (1).

#### Remark 1.1

The BV-BFV formulation of abelian Chern–Simons theory can be extended—as a $\mathbb {Z}_2$-graded theory—to dimension $d = 1$, see Sect. 3. Instead of $\mathbb {R}$-valued forms, there one has to consider forms with values in an odd vector space $\Pi \mathfrak {g}$, with $\mathfrak {g}$ an ordinary vector space equipped with an inner product.

This is the abelian version of the model studied in [2].

Let us explain now how to define the BV-BFV partition function. We will be very brief here; for a detailed exposition we refer to Cattaneo et al. [13]. We will require some additional pieces of data. The first one is a polarization $\mathcal {P}$ (involutive lagrangian distribution) on $\mathcal {F}^\partial _{\partial N}$. We say that the boundary 1-form $\alpha ^\partial$ is compatible with $\mathcal {P}$ if it vanishes on vectors belonging to $\mathcal {P}$. Typically this is not the case, but it may be achieved by means of the symmetry $\alpha ^\partial \rightarrow \alpha ^\partial + \delta f$ discussed above. Denote by $\mathcal {B}$ the leaf space of the polarization. In the examples of this paper we actually have $\mathcal {F}^\partial _{\partial N} \cong T^*\mathcal {B}$.

#### Remark 1.2

In the examples in this paper the graded manifold $\mathcal {F}^\partial _{\partial N}$ is actually a vector space, and the simplest polarizations are splittings into complex lagrangian subspaces $\mathcal {F}^\partial _{\partial N} \otimes \mathbb {C}= \mathcal {B} \oplus \mathcal {B}'$, we call those *linear polarizations*. However, it is interesting to consider more general polarizations. An example is the *Hitchin polarization* on $\Omega ^3(M,\mathbb {C})$ explained in Sect. 6.3.2.

Next, we require a splitting $\mathcal {F}_N \cong \mathcal {B}\times \mathcal {Y}$ where $\mathcal {Y}$ is also an odd symplectic vector space. Finally, we choose the data of a gauge fixing on $\mathcal {Y}$: another splitting $\mathcal {Y}\cong \mathcal {V}\times \mathcal {Y}'$ into odd symplectic vector spaces and a lagrangian $\mathcal {L}\subset \mathcal {Y}'$ such that 0 is an isolated critical point of $\mathcal {S}_N$ when restricted to $\mathcal {B}\times \mathcal {V}\times \mathcal {L}\subset \mathcal {B}\times \mathcal {V}\times \mathcal {Y}' \cong \mathcal {F}_M$, fiberwise over $\mathcal {B}\times \mathcal {V}$. The odd symplectic space $\mathcal {V}$ is called the space of residual fields and $\mathcal {L}$ is called the gauge-fixing lagrangian.

Given all these data, we can define the perturbative partition function as the integral of the exponentiated BV action over $\mathcal {L}$:

$$\begin{aligned} Z_N(\textsf{A},\textsf{a}) = \int _{\alpha \in \mathcal {L}\subset \mathcal {Y}'}\mathcal {D}\alpha \exp \left( \frac{i}{\hbar }\mathcal {S}^f_N(\textsf{A},\textsf{a},\alpha )\right) = \exp \left( \frac{i}{\hbar }S_\textrm{eff}(\textsf{A},\textsf{a}) \right) . \end{aligned}$$

The partition function *Z* and the effective action $S_\textrm{eff}$ are both functions on $\mathcal {B}\times \mathcal {V}$.

The integral is defined as a sum over Feynman diagrams—i.e., modeled on finite-dimensional Gaussian integrals. As a consequence of the structural Eq. (1), one expects $Z_N$ to satisfy the modified quantum master equation (mQME)

2 $$\begin{aligned} (\Omega _\mathcal {B}- \hbar ^2\Delta _\mathcal {V})Z_N = 0, \end{aligned}$$

where $\Delta _\mathcal {V}$ is the BV operator acting on functions on the odd symplectic vector space $\mathcal {V}$ of residual fields, given in Darboux coordinates $(q^i,p_i)$ by $\sum _i \pm \frac{\partial }{\partial q^i}\frac{\partial }{\partial p_i}$, and $\Omega _\mathcal {B}$ is a quantization of the BFV action $\mathcal {S}^\partial _{\partial N}$ acting on functions on $\mathcal {B}$ as a differential operator. If we write $\mathcal {F}^\partial _{\partial N} = T^*\mathcal {B}\ni (b,b')$, then $\Omega _\mathcal {B}$ is given by $\mathcal {S}^\partial _{\partial N}(b,-i\hbar \frac{\partial }{\partial b})$, with all derivatives to the right. At lowest order in $\hbar$, we have $\Omega _\mathcal {B}^2 = 0$ as a consequence of $(S,S)=0$. To ensure this to all orders, one might have to add higher order corrections (although there is no guarantee in general that the corrections exist). In all problems considered in this paper, $\Omega _\mathcal {B}$ squares to zero without further corrections (see Theorem A).

Since these operators anticommute with each other and square to zero, there is a double complex where $Z_N$ defines a cohomology class $[Z_N]$. This cohomology class is invariant under deformation of the choices made in the construction. For more details on the mQME (2), we refer to Cattaneo et al. [11, 13].

#### Remark 1.3

(*Choice of residual fields*) The choice of the space $\mathcal {V}\subset \mathcal {Y}$ is not unique. In fact, there is a partially ordered set of such choices, with maximal element $\mathcal {Y}$ and a minimal element $\mathcal {V}_\textrm{min}$, and one can pass from a bigger to a smaller choice by a BV pushforward. A more detailed discussion can be found in [13, Appendix F]. In this paper, when we deal with dimensions $d \ne 1$, we usually first have a “big” (infinite-dimensional) choice of $\mathcal {V}$. In some cases we are able to compute the BV pushforward to $\mathcal {V}_\textrm{min}$.

### Main results of the paper

We are now ready to describe the main results of this paper. We consider only spacetime manifolds *N* that are cylinders: $N = I \times \Sigma$. We think of the interval as $I = [0,1]$, so that $\partial N = \{0\} \times \Sigma \, \sqcup \, \{1\} \times \Sigma$, and we denote by $\Sigma _\textrm{in},\Sigma _\textrm{out}$ the two components. The BFV space of boundary fields $\mathcal {F}^\partial _{\partial N}$ then splits as $\mathcal {F}^\partial _{\partial N} = \mathcal {F}^\partial _\textrm{in}\times \mathcal {F}^\partial _\textrm{out}$.

We will consider polarizations of the space of boundary fields $\mathcal {F}^\partial _{\partial N}$ that are products of two polarizations on the two factors. We will work mostly with linear polarizations, i.e., splittings $\mathcal {F}^\partial _{\Sigma }\otimes \mathbb {C}= \mathcal {B}\oplus \mathcal {B}'$ where $\mathcal {B},\mathcal {B}'$ are complementary complex lagrangian subspaces of $\mathcal {F}^\partial _{\Sigma }\otimes \mathbb {C}$, so that we have an injection $\omega _\Sigma ^\sharp :\mathcal {B}' \rightarrow \mathcal {B}^*$. We will then write (suppressing the complexification) $\mathcal {F}^\partial _\Sigma \cong T^*\mathcal {B}$ and say that we are using the $\mathcal {B}$-representation.^5^ In ghost number 0 we also allow nonlinear polarizations with smooth leaf space $\mathcal {B}$ such that $F^\partial _\Sigma \cong T^*B$.^6^

Consider now a representation $\mathcal {F}^\partial _{\partial N}\cong T^*\mathcal {B}_\textrm{in}\times T^*\mathcal {B}_\textrm{out}$. Denote by $\mathcal{E}\mathcal{L}$ the zero locus of *Q*. It consists of (nonhomogeneous) closed forms in the abelian case and of “flat” nonhomogoneous forms in the nonabelian one. We call the projection $\mathcal {L}:=\pi (\mathcal{E}\mathcal{L})\subset \mathcal {F}^\partial _{\partial N}$ the *BV evolution relation*.

Denoting by $F^\partial _{\partial N}$ the ghost number 0 part, we get a product of two ordinary cotangent bundles $F^\partial _{\partial N} \cong T^*B_\textrm{in}\times T^*B_\textrm{out}$. We denote the restriction of the graded evolution relation by $L := \mathcal {L}|_{\textrm{gh}= 0}$ and call it simply the *evolution relation*. One can show that it is a lagrangian subspace and that it consists of boundary fields that can be extended to solutions of the Euler–Lagrange equations. A generalized generating function for *L* is given by the Hamilton–Jacobi action $S_{\textrm{HJ}}[b_\textrm{in},b_\textrm{out},e] \in C^\infty (B_\textrm{in}\times B_\textrm{out}\times V_{\textrm{aux}})$, where $V_\textrm{aux}$ is a space of additional parameters. The requirement on $V_\textrm{aux}$ is that in the fiber of $F_N$ over any

triple $(b_\textrm{in},b_\textrm{out},e) \in B_\textrm{in}\times B_\textrm{out}\times V_{\textrm{aux}}$ there exists a unique solution to the equations of motion. There is a poset of choices for this space. This is discussed in detail in the companion paper [14] and recalled in the Sect. 2 below. A first set of results can then be summarized as follows.

#### Theorem A

Consider one of the following BV-BFV theories:

1. 1D abelian Chern–Simons theory with linear or nonlinear polarizations,
2. $d = 4l+3$-dimensional Chern–Simons theory with linear or nonlinear polarizations,
3. 3-dimensional nonabelian Chern–Simons theory with linear polarizations.

Then there exists a space of residual fields $\mathcal {V}$ and a gauge-fixing lagrangian $\mathcal {L}$ such that the ghost number 0 component of $\mathcal {V}$ coincides with $V_\textrm{aux}$ and the ghost number 0 component of $S_\textrm{eff}$ coincides with $S_{\textrm{HJ}}$.

Here the choice of space of residual fields $\mathcal {V}$ is determined in ghost number 0 by the requirement that it should be isomorphic to $V_\textrm{aux}$. In all cases in this paper, the gauge-fixing Lagrangian is the space of 0-forms along the interval *I* intersected with $\mathcal {Y}$.^7^ In particular, there are no quantum corrections to the ghost number 0 part of the effective action (notice that the HJ action can be computed, as shown in [14, Section 7], completely at the classical level). The leading term in the effective action was expected to be the Hamilton–Jacobi action from the finite-dimensional results in the companion paper [14] (see in partiuclar Theorem 11.4 there). This theorem is nothing but an expression of the fact that to leading order the quantum theory is determined by the Euler-Lagrange locus. The fact that there are no quantum corrections is probably not surprising for the abelian Chern–Simons theory. For nonabelian Chern–Simons theory, the absence of quantum corrections is due to the fact that, given a complex structure on $\Sigma$, the interaction term is affine in both the holomorphic and antiholomorphic components of the connection along $\Sigma$, and the fact that our polarizataion and gauge fixing are compatible with this complex structure.

Our second main result concerns the mQME.

#### Theorem B

In all cases of Theorem A with linear polarizations, the BV-BFV partition function *Z* satisfies the modified quantum master equation

$$\begin{aligned} (\Omega - \hbar ^2 \Delta )Z = 0 \end{aligned}$$

with $\Omega = \Omega _{\mathcal {B}_\textrm{in}} + \Omega _{\mathcal {B}_\textrm{out}}$ given by the standard quantization (i.e. with derivatives to the right of multiplication operators) of the boundary action at both endpoints. For nonlinear polarizations $F^\partial _{\Sigma } = T^*B \ni (b,b')$, the mQME is satisfied whenever the constraint $d_{\Sigma }A = 0$ is linear in the momenta $b'$.

Again, in this case there are no quantum corrections to $\Omega$. These theorems summarize the results obtained in the various sections of this paper, where we discuss the different examples individually. We will outline the paper in slightly more detail in Sect. 1.3 below. Before that, let us comment on some of the more specific results in more detail.

#### Three-dimensional abelian Chern–Simons theory

In three-dimensional Chern–Simons theory, in ghost number 0 we have the lagrangian splitting

$$\begin{aligned} F^\partial _\Sigma \otimes \mathbb {C}= \Omega ^1(\Sigma ,\mathbb {C}) = \Omega ^{1,0}(\Sigma ) \oplus \Omega ^{0,1}(\Sigma ). \end{aligned}$$

For instance, one can define

$$\begin{aligned} \mathcal {B}_\textrm{out}= \Omega ^0(\Sigma ,\mathbb {C}) \oplus \Omega ^{1,0}(\Sigma ) \ni (\textsf{A}^0_\textrm{out},\textsf{A}^{1,0}_\textrm{out}) \end{aligned}$$

and

$$\begin{aligned} \mathcal {B}_\textrm{in}= \Omega ^{0,1}(\Sigma ) \oplus \Omega ^2(\Sigma ,\mathbb {C})\ni (\textsf{A}^{0,1}_\textrm{in},\textsf{A}^2_\textrm{in}). \end{aligned}$$

As discussed in Sect. 4.2, a possible choice for the space of residual fields is

$$\begin{aligned} \mathcal {V}_\textrm{small} = \{dt\cdot (\textsf{A}^0_{I\,\textrm{res}}+\textsf{A}^2_{I\,\textrm{res}})+(1-t)\cdot \textsf{A}^0_\textrm{res}+ t\cdot \textsf{A}^2_\textrm{res}\} \subset \Omega ^\bullet (I \times \Sigma ,\mathbb {C}), \end{aligned}$$

where $\textsf{A}^k_{I\,\textrm{res}}, \textsf{A}^k_\textrm{res}$ are complex valued *k*-forms on $\Sigma$, *t* is the coordinate on $I = [0,1]$ and the ghost numbers are $\textrm{gh}(\textsf{A}^k_{I\,\textrm{res}}) =-k, \textrm{gh}(\textsf{A}^k_\textrm{res}) = 1-k$. We will denote by $\sigma := A^0_{I\,\textrm{res}}$ the only ghost number 0 field in $\mathcal {V}_\textrm{small}$. The BV-BFV partition function is then computed as

$$\begin{aligned} Z_\textrm{small}= & {} \exp \frac{i}{\hbar } \Big (\underbrace{\int _\Sigma \Big ( \textsf{A}^{1,0}_\textrm{out}\textsf{A}^{0,1}_\textrm{in}+(\partial \textsf{A}^{0,1}_\textrm{in}+\bar{\partial }\textsf{A}^{1,0}_\textrm{out})\; \sigma +\frac{1}{2} \sigma \partial \bar{\partial }\sigma \Big )}_{S_\text {HJ}}\\{} & {} + \,\int _\Sigma \Big (-\textsf{A}^0_\textrm{out}\textsf{A}^2_\textrm{in}+\textsf{A}^0_\textrm{out}\textsf{A}^2_\textrm{res}-\textsf{A}^2_\textrm{in}\textsf{A}^0_\textrm{res}+ \frac{1}{2} \textsf{A}^2_\textrm{res}\textsf{A}^0_\textrm{res}\Big ) \Big ). \end{aligned}$$

In particular, focusing on the summand of the effective action in the first line, we recognize the Hamilton–Jacobi action from Example 2.2, as an instance of Theorem A. It is the action functional of a 2D free boson conformal field theory, coupled to the boundary fields $\textsf{A}^{1,0}_\textrm{out}$ and $\textsf{A}^{0,1}_\textrm{in}$. We arrive at the same result in ghost number 0 in Sect. 5.3.1, using $\mathcal {B}_\textrm{in}= \Omega ^0(\Sigma ,\mathbb {C}) \oplus \Omega ^{0,1}(\Sigma )$. One can integrate out the remaining residual fields to obtain then the fact that the partition function of three-dimensional Chern–Simons theory for the minimal space of residual fields (cf. Remark 1.3) coincides with the partition function of the 2D free boson CFT. In particular, one can observe the Weyl anomaly in the 3D Chern–Simons partition function. See Remark 4.3.

#### Three-dimensional nonabelian Chern–Simons theory and CS-WZW correspondence

The same lagrangian splitting as above can be used to study the 3D nonabelian Chern–Simons theory—see Sect. 5.3. The representation we use in that section is $\mathcal {F}^\partial _{\partial N} = T^*\mathcal {B}_\textrm{in}\times T^*\mathcal {B}_\textrm{out}$ with

$$\begin{aligned} \mathcal {B}_\textrm{out}= \Omega ^0(\Sigma ,\mathcal {G}_\mathbb {C}) \oplus \Omega ^{1,0}(\Sigma ,\mathcal {G}) \ni (\textsf{A}^0_\textrm{out},\textsf{A}^{1,0}_\textrm{out}) \end{aligned}$$

and

$$\begin{aligned} \mathcal {B}_\textrm{in}= \Omega ^0(\Sigma ,\mathcal {G}_\mathbb {C}) \oplus \Omega ^{0,1}(\Sigma ,\mathcal {G}). \end{aligned}$$

As a space of residual fields one can use

$$\begin{aligned} dt\cdot \Omega ^0(\Sigma ,\mathcal {G}_\mathbb {C}) \oplus \Omega ^2(\Sigma ,\mathcal {G}_\mathbb {C})[-1] \ni (dt\cdot \sigma , \textsf{A}^*_\textrm{res}) \end{aligned}$$

with $\textrm{gh}(\sigma ) = 0, \textrm{gh}(\textsf{A}^*_\textrm{res}) = -1$. We compute the effective action in Lemma 5.12 and see that it has a tree part $S^{\textrm{eff}(0)}$ and a 1-loop part $\mathbb {W}$:

$$\begin{aligned} S^\textrm{eff}= S^{\textrm{eff}(0)} - i\hbar \mathbb {W} = S^{\textrm{eff}(0)}_\textrm{ph}+ S^{\textrm{eff}(0)}_\textrm{gh}- i\hbar \mathbb {W} \end{aligned}$$

(the subscript $\textrm{ph}$ denotes the terms involving only fields of ghost number 0, the subscript $\textrm{gh}$ denotes terms involving fields with nonzero ghost number). At first glance the explicit formula (61) seems obscure, but we observe a number of interesting phenomena:

(i) One has to restrict the residual field $\sigma$ to a certain “Gribov region” $B_0 \subset \mathcal {G}_\mathbb {C}$—a region where the exponential map $\exp :\mathcal {G}_\mathbb {C}\rightarrow G_\mathbb {C}$ is injective—to make sure that certain power series appearing in $S^{\textrm{eff}(0)}_\textrm{gh}$ converge (Remark 5.13).
(ii) As shown in Lemma 5.14, when we restrict $\sigma$ to $B_0$, we can reparametrize by $g = e^{-\sigma }:\Sigma \rightarrow G_\mathbb {C}$. In this reparametrization, we can rewrite $S^{\textrm{eff}(0)}_\textrm{ph}$ as
  $$\begin{aligned} S^{\textrm{eff}(0)}_\textrm{ph}= \int _\Sigma \Big ( \langle \textsf{A}^{1,0}_\textrm{out}, g\, \textsf{A}^{0,1}_\textrm{in}g^{-1} \rangle - \langle \textsf{A}^{1,0}_\textrm{out},\bar{\partial }g\cdot g^{-1} \rangle - \langle \textsf{A}^{0,1}_\textrm{in}, g^{-1}\partial g \rangle \Big ) +\textrm{WZW}(g) \end{aligned}$$
  with the Wess–Zumino–Witten term
  $$\begin{aligned} \textrm{WZW}(g)=- \frac{1}{2} \int _\Sigma \langle \partial g\cdot g^{-1}, \bar{\partial }g\cdot g^{-1} \rangle -\frac{1}{12} \int _{\Sigma \times I}\langle d h\cdot h^{-1},[d h\cdot h^{-1},d h\cdot h^{-1}] \rangle \end{aligned}$$
  and $h = e^{(t-1)\sigma }.$ This coincides with the Hamilton–Jacobi action of Chern–Simons theory, see Example 2.3.
(iii) The term $-i\hbar \mathbb {W}$ in principle violates Theorem A and is divergent. However, it has a nice interpretation as a change of path integral measure from $\mathcal {D}\sigma$ to $\mathcal {D}g$, see Sect. 5.3.4. In particular, if one interprets *Z* as a *half-density* rather than a function on the space of residual fields, and thus $S^\textrm{eff}$ as a log-half-density, the effective action has no quantum corrections in the $(g,g^*)$ coordinates on $\mathcal {V}$ (here $g^*$ is the Darboux coordinate for *g*). It is in this sense that Theorem A holds.
(iv) In Sect. 5.3.6 we show that *Z* satisfies the modified quantum master equation in the different interpretations of *Z* (partition function vs. half-density). Interestingly, in the $(g,g^*)$ representation the mQME implies the well-known Polyakov–Wiegmann identity for the WZW action.

We thus observe a strong version of the CS-WZW correspondence: Namely, the effective theory of nonabelian Chern–Simons theory on $I \times \Sigma$ is a “gauged WZW theory,” i.e., a WZW theory on $\Sigma$ coupled to chiral gauge fields $\textsf{A}^{1,0}_\textrm{in},\textsf{A}^{0,1}_\textrm{out}$.

We also compute expectation values of vertical Wilson lines (Sect. 5.3.7) and show that they are given by field insertions in this WZW theory. This extends the CS-WZW correspondence to the level of observables. See the discussion in Sect. 5.3.8.

Formally, after integrating over the residual group-valued field *g*, the Chern–Simons partition function agrees with the partition function of the gauged WZW theory. One can use this to heuristically show the holomorphic factorization of the WZW model, as argued in Sect. 5.3.9.

Different versions of the relation between nonabelian Chern–Simons theory and the WZW model were studied in the literature before. A connection somewhat close to the one we are discussing appeared in [9, Section 4]; one important difference is that we are focusing on the homological (BV-BFV) aspects obtaining WZW as an effective BV theory. The other point is that the logic of our computation is different (it is a pure perturbative computation; it does not rely on quantum gauge invariance but has it as a result), see Remark 5.22.

#### Seven-dimensional Chern–Simons theory and the CS-BCOV correspondence

Finally, let us consider seven-dimensional Chern–Simons theory on a cylinder $N = I \times M$ with *M* a Calabi–Yau manifold. In particular, the complex structure on *M* defines a lagrangian splitting of $F^\partial _{M} = \Omega ^3(M,\mathbb {C})$:

$$\begin{aligned} \Omega ^3(M, \mathbb {C}) = X^+ \oplus X^- , \quad X^+ = \Omega ^{3,0}(M) \oplus \Omega ^{2,1}(M), \quad X^- = \Omega ^{1,2}(M) \oplus \Omega ^{0,3}(M). \end{aligned}$$

This lagrangian splitting determines a polarization of $F^\partial _M$.

On a Calabi–Yau manifold, however, there is another polarization of $F^\partial _M$ due to Hitchin [27]. Namely, a complex three-form *A* on *M* which is not itself decomposable, i.e., a wedge product of three 1-forms on *M*, has a decomposition $A = A^{+,\textrm{nl}} + A^{-,\textrm{nl}}$ where $A^{\pm ,\textrm{nl}}$ are decomposable three-forms uniquely defined up to exchange of $+$ and −. This polarization is discussed in Sect. 6.3.2.

We can compute the partition function *Z* on the cylinder with

$$\begin{aligned} \mathcal {B}_\textrm{in}= \Omega ^{\le 2}(M,\mathbb {C}) \oplus X^+ \ni (\textsf{c}_\textrm{in},\textsf{A}_\textrm{in}^{+,\textrm{l}}) \end{aligned}$$

and

$$\begin{aligned} \mathcal {B}_\textrm{out}= \Omega ^{\le 2}(M,\mathbb {C}) \times X^{-,\textrm{nl}} \ni (\textsf{c}_\textrm{out},\textsf{A}_\textrm{out}^{-,\textrm{nl}}). \end{aligned}$$

In this case, Theorem A holds—as shown in Sect. 5.2—and Theorem B holds because the constraint $d_MA = 0$ is linear in the momentum $A^{+,\textrm{nl}}$. Thus, the physical part of the effective action coincides with the Hamilton–Jacobi action computed in [14, Section 7.6] and is given bywith no quantum corrections in our choice of gauge fixing. Here $\textsf{A}^{p,q}_{I\,\textrm{res}}$ denote 2-forms of Hodge type (*p*, *q*) which are the residual fields of ghost number 0, and $G(A^{+,\textrm{l}},A^{-,\textrm{nl}})$ is the generating function satisfying $\delta G = A^{-,\mathrm l}\delta A^{+,l} - A^{+,\textrm{nl}}\delta A^{-,\textrm{nl}}$. Since the partition function *Z* satisfies, by Theorem B, the modified quantum master equation, when changing the gauge fixing the partition function changes by an $(\Omega - \hbar ^2\Delta )$-exact term.

The partition function *Z* can be interpreted as the integral kernel of a generalized Segal–Bargmann transform, see Appendix A. We thus show that the approximation used by Gerasimov and Shatashvili in [24]—where they were only assuming this representation to be true in the semiclassical limit—is exact.

Following Gerasimov and Shatashvili [24], we can then relate the Chern–Simons partition function to the partition function of Kodaira–Spencer or BCOV theory, defined in [8] and recalled in Appendix B, as follows. One can consider a certain ($\Omega$-closed) state $\psi (\textsf{A}_\textrm{out}^{-,\textrm{nl}},\textsf{c}_\textrm{out})$ in the $A^{-,\textrm{nl}}$-representation. We then apply the operator *Z* to $\psi$—by multiplying and formally integrating over $\mathcal {B}_\textrm{out}$—and show that the result $Z\cdot \psi$ is still $(\Omega - \hbar ^2\Delta )$-closed. Next we identify a gauge-fixing lagrangian $\mathcal {L}\subset \mathcal {V}$ and compute $Z''[\textsf{A}^{3,0}_\textrm{in},\textsf{A}^{2,1}_\textrm{in},c_\textrm{in}] = \int _\mathcal {L}Z\cdot \psi .$ One can then show that in ghost number 0

$$\begin{aligned} Z''_\textrm{ph}[\textsf{A}^{3,0}_\textrm{in}=\omega _0,\textsf{A}^{2,1}_\textrm{in}= x] \sim Z_{KS}[x] , \end{aligned}$$

where $\omega _0$ is a normalized generator of $H_{\partial }^{3,0}(M)$, *x* is a $\partial$-harmonic form, and $Z_{KS}[x]$ is the Kodaira–Spencer partition function with background *x*. For the precise statement see Sect. 6.3.3. In particular, we see that this statement holds not only in the semiclassical approximation to $Z_{CS}$ as in [24], but that it is exact. For general boundary conditions $\textsf{A}^{3,0}_\textrm{in},\textsf{A}^{2,1}_\textrm{in}$, the Chern–Simons partition function can be computed from the mQME.

### Structure of the paper

We summarize the remaining results by outlining the structure of the paper.

In Sect. 2, we recall the construction of the Hamilton–Jacobi action from Cattaneo et al. [14], and the important examples (abelian and nonabelian Chern–Simons theory) from that paper.

In Sect. 3, we consider as a warm-up the example of the abelian 1D CS theory. This is the 1D AKSZ theory with target a vector space $\mathfrak {g}$ that we assume to have an inner product and a compatible complex structure *J*, so that $\mathfrak {g}\otimes \mathbb {C}= \mathfrak {g}^+ \oplus \mathfrak {g}^-$ splits into $\pm i$-eigenspaces of *J*. We then compute the partition function for both $\mathcal {B}_\textrm{in}= \mathcal {B}_\textrm{out}= \mathfrak {g}^+$ in Sect. 3.1 and $\mathcal {B}_\textrm{in}=\mathfrak {g}^-, \mathcal {B}_\textrm{out}= \mathfrak {g}^+$ in Sect. 3.2 and comment briefly on the Theorems A and B in this context (which are in this case rather trivial).

In Sect. 4, we consider the 3D abelian Chern–Simons theory on $I\times \Sigma$ as a 1D theory with values in $\mathfrak {g}= \Omega ^\bullet (\Sigma )$. Choosing a complex structure on $\Sigma$, we split $\mathfrak {g}= \mathfrak {g}^+ \oplus \mathfrak {g}^-$ and consider $\mathcal {B}_\textrm{in}= \mathcal {B}_\textrm{out}= \mathfrak {g}^+$ in Sect. 4.1 and $\mathcal {B}_\textrm{in}=\mathfrak {g}^-, \mathcal {B}_\textrm{out}= \mathfrak {g}^+$ in Sect. 4.2. In both cases, we comment on the HJ and mQME properties, and in the second case also the pushforward to the minimal space of residual fields and the relation to the 2D free boson CFT is discussed.

In Sect. 5, we consider the case where $\mathcal {B}_\textrm{in}$ and $\mathcal {B}_\textrm{out}$ both have components only in nonnegative ghost number, and agree in positive ghost number. We call these “parallel ghost polarization”. In Sect. 5.1, we consider 1D Chern–Simons theory with values in a complex, with opposite linear polarizations in ghost number 0. In Sect. 5.2, we consider the same theory with a possibly nonlinear polarization on the $\textrm{out}$-boundary. These subsections serve as a toy model for the higher-dimensional Chern–Simons theories considered later. In Sect. 5.3, we return to the three-dimensional Chern–Simons theory, with opposite linear polarization in degree 0. After briefly studying again the abelian case in Sect. 5.3.1, we discuss the nonabelian case in more detail, the results are summarized already in Sect. 1.2.2 above. Finally in Sect. 5.4, we consider the nonabelian theory with parallel polarizations both in the ghost and physical sectors.

In Sect. 6, we turn to Chern–Simons theories of arbitrary dimension. We consider both linear polarizations that are transversal in the ghost sector at opposite ends (Sect. 6.1) and parallel in the ghost sector (Sect. 6.2). Finally in Sect. 6.3 we turn our attention to nonlinear polarizations at one boundary, in particular the 7D case with Hitchin polarization, that was summarized in Sect. 1.2.3 above.

The appendices contain some complementary material. In Appendix A we show how to recover the usual Segal–Bargmann transform as a BV-BFV partition function on an interval with a particular choice of boundary polarizations. This is an illustration of the maxim that topological partition functions on cylinders yield instances of generalized Segal–Bargmann transforms. We also comment on the contour integration in the complexified space of fields. In Appendix B, we recall very briefly the Kodaira–Spencer theory of deformations of complex structures and the BCOV action functional.

### Outlook

Finally, let us point out some interesting directions for further research.

- All our partition functions depend nontrivially on the choice of complex structure on the boundary.^8^ This dependence should be described by extending the partition function to a (projectively flat) section of a vector bundle over the moduli space of complex structures on the boundary, for instance the one constructed in [4].
- Recently [31] it has been suggested that the partition function of a 3D *U*(1) Chern–Simons theory can be computed by averaging over Narain moduli space of boundary CFT’s. We believe our methods could be generalized to include nontrivial flat bundles and we plan to investigate this proposal.
- Our results on the CS-WZW correspondence strongly suggest that the space of *n*-point conformal blocks can be described as the $\Omega$-cohomology (see Sect. 5.3.8; the genus-zero case of this statement was a result of [1]). This would provide an interesting new description of the space of conformal blocks. We also hope it would lead to a better understanding of the relationship between Chern–Simons theory and the KZ(B) connection.
- It would be highly interesting to compare our findings on the CS-BCOV correspondence to other approaches to the subject such as [15].
- Another proposal to compute holographic duals of action functionals from BV-BFV formalism on manifolds with boundary was made by the second and third authors together with Schiavina [33]. The point of view there was more focused on descent equations and extensions to higher codimension, while the present paper emphasizes the role of the BV effective action. The relationship between the two constructions needs to be explored.
- In [25], the authors show that there exists a 1-loop exact quantization of Chern–Simons theory on $\mathbb {R}^3$, which is similar to the result that we obtain here (in our case, the wheels appear only in the ghost sector of the theory). The gauge fixing they use is different from ours, and the focus there is not on partition functions, but rather on the anomaly-freeness of the theory, a problem which does not appear in our gauge fixing. Nevertheless it would be interesting to investigate this gauge-fixing from the BV-BFV viewpoint and compare it with our current results.

### Notations and conventions

This is a quantum paper and notations fluctuate. Fixing one makes a complementary one explode.

In this paper we study field theories on cylinders $N = I \times \Sigma$ from different viewpoints, with $I=[0,1]$ the interval with its standard orientation, and a $\Sigma$ a $(d-1)$-dimensional closed oriented manifold. Notations are adapted to the individual sections.

We are considering Chern–Simons-type theories, in different dimensions and with different targets. The Chern–Simons superfield is denoted $\mathcal {A} \in \Omega ^\bullet (I \times \Sigma , \Pi \mathfrak {g})$.

When we are considering 1-dimensional theories (with a possibly infinite-dimensional target) as in Sects. 3, 5.1 and 5.2, we denote the components of the superfield $\mathcal {A} = \psi + A$, where $\psi \in \Omega ^0(I,\mathfrak {g})$ and $A \in \Omega ^1(I,\mathfrak {g})$. Decoration of $\psi , A$ with superscripts denotes components with respect to a splitting of $\mathfrak {g}$. Decoration of $\psi ,A$ with subscripts denotes components with respect to a splitting of $\Omega ^\bullet (I)$. Typical subscripts are $\textrm{in}$ and $\textrm{out}$, denoting fields supported on the $\textrm{in}$ or $\textrm{out}$ boundary (elements of $\mathcal {B}_\textrm{in}$ or $\mathcal {B}_\textrm{out}$) respectively, $\textrm{res}$ for residual fields (elements of $\mathcal {V}$), and $\textrm{fl}$ for fluctuations (elements of $\mathcal {L}$).

When we are thinking about higher-dimensional theories (still on cylinders) as in Sects. 4 and 6, we denote the components of $\mathcal {A} = \textsf{A}+ dt \cdot \textsf{A}_I$, with $\textsf{A},\textsf{A}_I\in \Omega ^{0,\bullet }(I \times \Sigma )$. Superscripts now denote components of homogeneous form degree in $\Sigma$.

In Sects. 5.3 and 5.4, it is convenient to revert to a more “traditional” notation $\mathcal {A} = c + A + A^* + c^*$, here the nonhomogeneous differential form is split according to form degree. There we also denote the (finite-dimensional) coefficient Lie algebra by $\mathcal {G}$.

## Constrained Systems and Generalized Hamilton–Jacobi Actions

We start with a short review of the results of [14] that are relevant for this paper. We focus on action functionals of the form^9^

$$\begin{aligned} S[p,q,e]=\int _I(pdq-\langle H(p,q),e\rangle ), \end{aligned}$$

where *I* is the interval [0, 1], (*p*, *q*) are coordinates on a given cotangent bundle $T^*B$ (and, by abuse of notation, also stand for a map from *I* to $T^*B$), *e* is a one-form on *I* taking value in some vector space $\mathfrak {h}$, and *H* is a given map $T^*B\rightarrow \mathfrak {h}^*$. The pairing between the *p* and the *q* coordinates is understood, whereas for the pairing between $\mathfrak {h}$ and its dual we use the notation $\langle \ ,\rangle$. In the applications of this paper the space $\mathfrak {h}$ and the manifold *B* are infinite-dimensional (typically, Fréchet spaces).

To be more precise, $T^*B$ denotes some given vector bundle over *B* with a nondegenerate pairing to *TB*; we denote by $\theta$ the canonical one-form on it (which we will also call the Noether 1-form in the following) and by $\omega =d\theta$ the canonical symplectic form; by $\mathfrak {h}^*$ we denote a given subspace of the dual of $\mathfrak {h}$ such that its pairing to $\mathfrak {h}$ is still nondegenerate. The first term in the action can also be written in coordinate-free way as $x^*\theta$ in terms of a path $x:I\rightarrow T^*B$. For the second term, we assume a given map *X* from $\mathfrak {h}$ to the vector fields on $T^*B$ and define, up to carefully chosen constants, the map *H* by $\iota _X\omega =d H$. (Note that *H* is a map from $\mathfrak {h}$ to the functions on $T^*B$, and we assume that, dually, it belongs to the chosen subspace $\mathfrak {h}^*$.)

### Example 2.1

(*3D Chern–Simons theory*). Consider 3D Chern–Simons theory for aquadratic Lie algebra $\mathcal {G}$ on $I\times \Sigma$, where $\Sigma$ is a closed oriented surface with a chosen complex structure. The complexified phase space is $T^*B=\Omega ^{1,0}(\Sigma )\otimes \mathcal {G}\oplus \Omega ^{0,1}(\Sigma )\otimes \mathcal {G}$ with $B=\Omega ^{0,1}(\Sigma )\otimes \mathcal {G}$. We then have $\mathfrak {h}=\Omega ^0(\Sigma )\otimes \mathcal {G}$ and $\mathfrak {h}^*=\Omega ^2(\Sigma )\otimes \mathcal {G}$. The pairings are induced by the given pairing on $\mathcal {G}$ and by integration on $\Sigma$. An element of $T^*B$ is a connection one-form, the map *X* yields the gauge transformations, and *H* is the curvature two-form.

We split the fields into two classes: the dynamical field (the map *x* to $T^*B$) and the Lagrange multiplier (the $\mathfrak {h}$-valued one-form *e*). We accordingly split the Euler–Lagrange (EL) equations into the evolution equation, the variations with respect to the dynamical field,

$$\begin{aligned} dx = \langle X,e \rangle , \end{aligned}$$

and the constraints, the variations with respect to the Lagrange multiplier,

$$\begin{aligned} H=0. \end{aligned}$$

Note that the constraints must be satisfied at every time.

We define the evolution relation *L* as the possible boundary values (at 0 and 1 in *I*) that a solution to the EL equations can have. Assuming it to be a (possibly immersed) submanifold, *L* turns out to be an isotropic submanifold of $\overline{T^*B}\times T^*B$ [10], where the bar means that we use the opposite symplectic form. We assume it to be actually split lagrangian (i.e., for every point *v* of *L*, its tangent space $T_vL$, which is isotropic in general, must have an isotropic complement).^10^ Thanks to the Hodge decomposition theorem, this assumption is satisfied in all the examples of this paper.

We then denote by *C* the projection of *L* on either factor $T^*B$ and we assume it to be a submanifold. As observed in [11], if *L* is lagrangian, then *C* is coisotropic. In particular, at every point $c\in C$ and for every $\xi \in \mathfrak {h}$, the vector $\langle X(c),\xi \rangle$ is tangent to *C*. Moreover, the span of these vectors at each point defines an involutive distribution on *C*, called the characteristic distribution (the reduced phase space of the theory is then defined as the reduction of *C* with respect to its characteristic distribution).^11^

In the case at hand, we have that *C* is the zero locus of *H*. The evolution equation, for a given *e*, is then the hamiltonian evolution for the (time-dependent) hamiltonian $\langle H,e\rangle$. Since *C* is coisotropic, this evolution does not leave *C*—so it is enough to implement the constraint $H=0$ at the initial, or final, endpoint—and lies along the characteristic distribution. It follows that the evolution relation *L* consists of pairs of points on *C* lying on the same leaf of the characteristic distribution.

Next we are interested in solutions to the EL equations. For this we have to fix boundary conditions; namely, we have to choose lagrangian submanifolds $L_0$ and $L_1$ of $T^*B$ at the endpoints of *I*, and we assume that the intersection of $L_0\times L_1$ with the evolution relation *L* is discrete.^12^ For simplicity, we will work with a unique solution. We are also interested in letting boundary conditions vary, so we consider families of lagrangian submanifolds (polarizations). Concretely, at the initial endpoint we take the $L_0$s to be the fibers of $T^*B$, which we then parametrize by *B*, whereas at the final endpoint we realize $T^*B$ as $T^*B'$, with $B'$ a possibly different manifold, and take the $L_1$s to be the fibers of $T^*B'$, which we then parametrize by $B'$.^13^ We want the variations of the action with the given boundary conditions not to have boundary terms. This is automatically satisfied at the initial point, where we take the polarization $T^*B$, but we have to adapt the action to the canonical one-form $\theta '$ of $T^*B'$ at the final endpoint. For this, we assume that there is a function *f* on $B\times B'$ such that $\theta = \theta ' + df$ and we modify the action to

$$\begin{aligned} S^f[p,q,e]:=S[x,e]-f(q(1),Q(p(1),q(1))), \end{aligned}$$

where *Q* is the base coordinate of $T^*B'$.

We define the Hamilton–Jacobi (HJ) action $S^f_\text {HJ}$ of the theory with respect to the given polarizations as the evaluation of $S^f$ on a solution (which we assume to be unique) to the evolution equation for each choice of *e*. Note that $S^f_{\text {HJ}}$ is a function on $B\times B'\times \Omega ^1(I,\mathfrak {h})\ni (q_\textrm{in},Q_\textrm{out},e)$. Also note that we do not impose the constraints in the definition of $S^f_{\text {HJ}}$. It was proved in [14] *i*) that $S^f_{\text {HJ}}$ is invariant under certain equivalence transformations of *e*, and *ii*) that it is a generalized generating function for the evolution relation *L* with respect to the given polarizations.

Let us elaborate on this. As for *i*), assume for simplicity that, as in every example of this paper, $\mathfrak {h}$ is actually a Lie algebra and *H* is an equivariant momentum map (for the infinitesimal action *X* of $\mathfrak {h}$ on $T^*B$). Then *e* may be regarded as a connection one-form on *I*. The equivalence transformations are in this case gauge transformations that are trivial at the endpoints. As for *ii*), the statement means that, upon setting to zero the variation of $S^f_{\text {HJ}}$ with respect to (the equivalence class of) *e*, we recover the final *P* variables of a solution as the variation of $S^f_{\text {HJ}}$ with respect to $Q_\textrm{out}$ and the initial *p* variables of a solution as minus the variation of $S^f_{\text {HJ}}$ with respect to $q_\textrm{in}$.

Explicit examples, relevant for this paper, are discussed in [14, Section 7]. We recall the results.

### Example 2.2

(*Abelian 3D Chern–Simons theory*). We use the notations of Example 2.1, but now with $\mathfrak {g}=\mathbb {R}$. We take the initial polarization as $T^*B$, with $B=\Omega ^{0,1}(\Sigma )$, and the final polarization as $T^*B'$, with $B'=\Omega ^{1,0}(\Sigma )$.^14^ We denote by $\partial$ and $\bar{\partial }$ the Dolbeault operators. The HJ action then reads

$$\begin{aligned} S^f_\text {HJ}=\int _\Sigma \left( \textsf{A}^{1,0}_\textrm{out}\textsf{A}^{0,1}_\textrm{in}+ \sigma (\bar{\partial }\textsf{A}^{1,0}_\textrm{out}+\partial \textsf{A}^{0,1}_\textrm{in})+\frac{1}{2} \sigma \partial \bar{\partial }\sigma \right) , \end{aligned}$$

with $$\textsf{A}^{0,1}_\textrm{in}\in B$$, $$\textsf{A}^{1,0}_\textrm{out}\in B'$$, and $$\sigma \in \Omega ^0(\Sigma )$$.

### Example 2.3

(*Nonabelian 3D Chern–Simons theory*). Again we use the notations of Example 2.1. The initial and final polarizations now are $T^*B$, with $B=\Omega ^{0,1}(\Sigma )\otimes \mathcal {G}$, and $T^*B'$, with $B'=\Omega ^{1,0}(\Sigma )\otimes \mathcal {G}$. We assume the exponential map from $\mathcal {G}$ to the its simply connected Lie group *G* to be surjective. In this case the gauge-invariant parameter $g\in {\text {Map}}(\Sigma ,G)$ is of the form $g=e^{-\sigma }$ with $\sigma \in {\text {Map}}(\Sigma ,\mathcal {G})$. The HJ action then reads

$$\begin{aligned} S^f_\text {HJ} = \int _\Sigma \Big ( \langle \textsf{A}^{1,0}_\textrm{out}, g\, \textsf{A}^{0,1}_\textrm{in}g^{-1} \rangle - \langle \textsf{A}^{1,0}_\textrm{out},\bar{\partial }g\cdot g^{-1} \rangle - \langle \textsf{A}^{0,1}_\textrm{in}, g^{-1}\partial g \rangle \Big ) +\textrm{WZW}(g) \end{aligned}$$

with the Wess–Zumino–Witten term

$$\begin{aligned} \textrm{WZW}(g)=- \frac{1}{2} \int _\Sigma \langle \partial g\cdot g^{-1}, \bar{\partial }g\cdot g^{-1} \rangle -\frac{1}{12} \int _{\Sigma \times I}\langle dh\cdot h^{-1},[dh\cdot h^{-1},dh\cdot h^{-1}] \rangle , \end{aligned}$$

where $$h=e^{(t-1)\sigma }$$.^15^

Thus, the HJ action of Chern–Simons theory can be identified with a “gauged WZW action” (see for instance [21]). This points at a deep relationship between these two theories.

## BV-BFV Approach Warm-Up: 1D Abelian Chern–Simons

As a warm-up exercise before the BV-BFV treatment of 3D Chern–Simons, let us consider one-dimensional abelian Chern–Simons theory^16^ on an interval $I=[0,1]$—the AKSZ theory with $\mathbb {Z}_2$-graded space of BV fields

$$\begin{aligned} \mathcal {F}=\textrm{Map}(T[1]I,\Pi \mathfrak {g})=\Omega ^\bullet (I)\otimes \Pi \mathfrak {g}. \end{aligned}$$

Here $\mathfrak {g}$ is a vector space of coefficients endowed with a nondegenerate inner product (, ) and $\Pi$ is the parity-reversal symbol. A vector in $\mathcal {F}$ is the superfield $\psi +A$, with $\psi$ a $\Pi \mathfrak {g}$-valued 0-form and *A* a $\mathfrak {g}$-valued 1-form, and the BV action is:

3 $$\begin{aligned} S(\psi +A)=\int _I\frac{1}{2} (\psi , d_I\psi ) \end{aligned}$$

with $d_I=dt \frac{d}{dt}$ the de Rham differential on the interval $t\in [0,1]$. The odd symplectic form on $\mathcal {F}$ is given by $\omega =-\int _I (\delta A, \delta \psi )$. The cohomological vector field (BRST operator) *Q* on $\mathcal {F}$ is defined by $Q:\psi \mapsto 0,\; A\mapsto d_I\psi$.

The BFV phase space assigned to a point is $\mathcal {F}^\partial _{pt}=\Pi \mathfrak {g}$, equipped with Noether 1-form $\alpha _{pt^\pm }=\pm \frac{1}{2}(\psi ,\delta \psi )$ where ± corresponds to the orientation of the point; the BFV action is zero,^17^$S_{pt}=0$. We are using the following sign convention for the BV-BFV structure relation:

4 $$\begin{aligned} \delta S=\iota _Q \omega +\pi ^* \alpha _\partial . \end{aligned}$$

Assume that $\mathfrak {g}$ is equipped with a complex structure $J\in \textrm{End}(\mathfrak {g})$, $J^2=-\textrm{Id}$, compatible with the inner product. We have a splitting of the complexification of $\mathfrak {g}$ into $\pm i$-eigenspaces of *J*:

5 $$\begin{aligned} \mathfrak {g}_\mathbb {C}=\mathfrak {g}^{+}\oplus \mathfrak {g}^{-} \end{aligned}$$

—the “holomorphic” and “antiholomorphic” subspaces of $\mathfrak {g}_\mathbb {C}=\mathfrak {g}\otimes \mathbb {C}$, which are lagrangian due to compatibility between *J* and (, ).

### Holomorphic-to-holomorphic boundary conditions

Consider the boundary polarization $\textrm{Span}(\frac{\partial }{\partial \psi ^{-}})$ imposed at both $t=0$ and $t=1$ (a.k.a. $\psi ^{+}-\psi ^{+}$ representation, as the partition function will depend on the boundary value $\psi ^{+}_\textrm{in}$ at $t=0$ and boundary value $\psi ^{+}_\textrm{out}$ at $t=1$). For compatibility with this polarization, we need to modify the action (3) by boundary terms:

6 $$\begin{aligned} S\mapsto S^f=S+\frac{1}{2} (\psi ^+,\psi ^-)\big |_{t=1}-\frac{1}{2} (\psi ^+,\psi ^-)\big |_{t=0} . \end{aligned}$$

Then the corresponding boundary 1-form is:

$$\begin{aligned} \alpha _{\partial I}^f= & {} \left. \left( \frac{1}{2}(\psi ,\delta \psi )+\delta \frac{1}{2}(\psi ^+,\psi ^-)\right) \right| _{t=1}- \left. \left( \frac{1}{2}(\psi ,\delta \psi )+\delta \frac{1}{2}(\psi ^+,\psi ^-)\right) \right| _{t=0}\\= & {} (\psi ^-,\delta \psi ^+)\big |_{t=1}- (\psi ^-,\delta \psi ^+)\big |_{t=0} \end{aligned}$$

—the canonical 1-form in the chosen representation, as desired (cf. Sect. 1.1; see [14, Section 9] and references therein for more details). The space of fields $\mathcal {F}$ is fibered over the base $\mathcal {B}=\Pi \mathfrak {g}^+\oplus \Pi \mathfrak {g}^+ =\{(\psi ^+_\textrm{in},\psi ^+_\textrm{out})\}$ with the fiber

$$\begin{aligned} \mathcal {Y}=\Omega ^\bullet (I,\partial I;\Pi \mathfrak {g}^+)\oplus \Omega ^\bullet (I;\Pi \mathfrak {g}^-) . \end{aligned}$$

Here the first summand on the r.h.s. is $\mathfrak {g}^+$-valued forms vanishing on the boundary and the second summand is $\mathfrak {g}^-$-valued forms with free boundary conditions. The cochain complex $\mathcal {Y}$ admits the following splitting (a Hodge decomposition):

7 $$\begin{aligned} \mathcal {Y}= & {} \underbrace{\big (dt\cdot \mathfrak {g}^+\oplus 1\cdot \Pi \mathfrak {g}^-\big )}_{\mathcal {V}}\nonumber \\{} & {} \bigoplus \underbrace{\big (\Omega ^0(I,\partial I;\Pi \mathfrak {g}^+)\oplus \Omega ^0_{\int =0}(I;\Pi \mathfrak {g}^-)\big )}_{\mathcal {Y}'_{K-ex}}\bigoplus \underbrace{\big (\Omega ^1_{\int =0}(I;\mathfrak {g}^+)\oplus \Omega ^1(I;\mathfrak {g}^-)\big )}_{\mathcal {Y}'_{d-ex}} . \end{aligned}$$

Here the first term (“residual fields”) is a deformation retract of $\mathcal {Y}$ (in this case, in fact, its cohomology). The subscript $\int =0$ means “forms with vanishing total integral” (against *dt* in 0-form case). The two last terms jointly form an acyclic subcomplex $\mathcal {Y}'$ of $\mathcal {Y}$, split into a *d*-exact part and its direct complement—the *K*-exact part, where $K:\mathcal {Y}^\bullet \rightarrow \mathcal {Y}^{\bullet -1}$ is the chain homotopy between identity and projection onto $\mathcal {V}$. Explicitly, *K* kills all 0-forms and acts on $\mathfrak {g}^+$- and $\mathfrak {g}^-$-valued 1-forms as follows:

8 $$\begin{aligned} K:\begin{array}{lcl} dt\,g^+(t) &{}\mapsto &{} \int _0^t dt' g^+(t')-t\int _0^1 dt' g^+(t') , \\ dt\, g^-(t) &{}\mapsto &{} -\int _t^1 dt' g^-(t')+ \int _0^1 dt'\, t'\, g^-(t') . \end{array} \end{aligned}$$

The integral kernel of *K* is the propagator:

9 $$\begin{aligned} \eta (t,t')=\pi ^+\otimes (\theta (t-t')-t)+\pi ^-\otimes (t'-\theta (t'-t)) , \end{aligned}$$

where $\pi ^\pm$ are the projectors from $\mathfrak {g}$ to $\mathfrak {g}^\pm$ and $\theta$ is the step function.

The BV-BFV partition function is given by the following path integral (see [13] for the general construction):

10 $$\begin{aligned}{} & {} Z(\psi ^+_\textrm{in},\psi ^+_\textrm{out}; \psi ^-_\textrm{res}, A^+_\textrm{res})\nonumber \\{} & {} \quad = \int _{\mathcal {Y}'_{K-ex}\subset \mathcal {Y}'} \mathcal {D} \psi ^+_\textrm{fl} \mathcal {D} \psi ^-_\textrm{fl} \; e^{\frac{i}{\hbar }S^f\left( \widetilde{\psi ^+_\textrm{in}}+\widetilde{\psi ^+_\textrm{out}}+\psi ^+_\textrm{fl}+\psi ^-_\textrm{res}+\psi ^-_\textrm{fl}+dt\cdot A^+_\textrm{res} \right) } . \end{aligned}$$

Here the notations are:

- $\widetilde{\psi ^+_\textrm{in}}$ is the discontinuous extension^18^ of $\psi ^+_\textrm{in}$ at $t=0$ by zero at $t>0$; likewise, $\widetilde{\psi ^+_\textrm{out}}$ is the discontinuous extension of $\psi ^+_\textrm{out}$ at $t=1$ by zero at $t< 1$;
- the “fluctuation” $(\psi ^+_\textrm{fl}, \psi ^-_\textrm{fl})\in \mathcal {Y}'_{K-ex}$ is the field we integrate over (while setting to zero the component in $\mathcal {Y}'_{d-ex}$ is the gauge fixing);
- $(\psi ^-_\textrm{res}, dt\cdot A^+_\textrm{res})\in \mathcal {V}$, with $\psi ^-_\textrm{res}\in \Pi \mathfrak {g}^-$ and $A^+_\textrm{res}\in \mathfrak {g}^+$, is the residual field.

Continuing the computation (10), we have the Gaussian integral

11 $$\begin{aligned} Z= & {} \int \mathcal {D}\psi ^+_\textrm{fl}\mathcal {D}\psi ^-_\textrm{fl}\; \exp \frac{i}{\hbar }\Big ( \underbrace{\frac{1}{2} \int _I \big ( \psi ^-_\textrm{res}+\psi ^-_\textrm{fl}, d_I(\widetilde{\psi ^+_\textrm{out}}+ \widetilde{\psi ^+_\textrm{in}}) \big )}_a \nonumber \\{} & {} +\,\underbrace{\frac{1}{2}\int _I \big (\widetilde{\psi ^+_\textrm{out}}+ \widetilde{\psi ^+_\textrm{in}}, d_I(\psi ^-_\textrm{res}+\psi ^-_\textrm{fl}) \big )}_b+ \underbrace{\frac{1}{2} \int _I \big ( \psi ^-_\textrm{res}+\psi ^-_\textrm{fl}, d_I\psi ^+_\textrm{fl}\big ) }_c\nonumber \\{} & {} +\, \underbrace{\frac{1}{2} \int _I \big (\psi ^+_\textrm{fl}, d_I(\psi ^-_\textrm{res}+\psi ^-_\textrm{fl})\big )}_d\nonumber \\{} & {} +\,\underbrace{\frac{1}{2} (\psi ^+_\textrm{out},\psi ^-_\textrm{res}+\psi ^-_\textrm{fl}\big |_{t=1})}_e \underbrace{- \frac{1}{2} (\psi ^+_\textrm{in},\psi ^-_\textrm{res}+\psi ^-_\textrm{fl}\big |_{t=0})}_f \Big ) \nonumber \\= & {} \int \mathcal {D} \psi ^+_\textrm{fl} \mathcal {D} \psi ^-_\textrm{fl} \; e^{\frac{i}{\hbar }\left( \int _I ( \psi ^-_\textrm{fl}, d_I\psi ^+_\textrm{fl}) +(\psi ^+_\textrm{out},\psi ^-_\textrm{res}+\psi ^-_\textrm{fl}(1))- (\psi ^+_\textrm{in},\psi ^-_\textrm{res}+\psi ^-_\textrm{fl}(0))\right) }\nonumber \\= & {} e^{\frac{i}{\hbar } (\psi ^+_\textrm{out}-\psi ^+_\textrm{in},\psi ^-_\textrm{res})} . \end{aligned}$$

Here the terms in the first expression above are:

- Term *a* is a pure boundary term, in fact $a=e+f$, which leads to $\frac{1}{2}$ factors of the boundary terms *e*, *f* being doubled and replaced by 1 in the second equality in (11).
- $$b=0$$.
- $$c=d=\frac{1}{2} \int _I (\psi ^-_\textrm{fl},d_I\psi ^+_\textrm{fl})$$.

### Antiholomorphic-to-holomorphic boundary conditions

Next, consider imposing the polarization $\frac{\partial }{\partial \psi ^+}$ at $t=0$ and $\frac{\partial }{\partial \psi ^-}$ at $t=1$ (a.k.a. $\psi ^--\psi ^+$ representation: we are fixing the boundary value $\psi ^-_\textrm{in}$ at $t=0$ and $\psi ^+_\textrm{out}$ at $t=1$). The “polarized action” (the counterpart of (6)) in this case is:

12 $$\begin{aligned} S^f=S+\frac{1}{2}(\psi ^+,\psi ^-)\big |_{t=1}+\frac{1}{2}(\psi ^+,\psi ^-)\big |_{t=0} \end{aligned}$$

and the corresponding boundary 1-form is:

$$\begin{aligned} \alpha ^f_{\partial I}= & {} \left. \left( \frac{1}{2}(\psi ,\delta \psi )+\delta \frac{1}{2}(\psi ^+,\psi ^-)\right) \right| _{t=1}- \left. \left( \frac{1}{2}(\psi ,\delta \psi )-\delta \frac{1}{2}(\psi ^+,\psi ^-)\right) \right| _{t=0}\\= & {} (\psi ^-,\delta \psi ^+)\big |_{t=1}- (\psi ^+,\delta \psi ^-)\big |_{t=0} . \end{aligned}$$

This 1-form vanishes along the chosen polarization, as desired.

Next, the fiber of the space of fields $\mathcal {F}$ over the base $\mathcal {B}=\Pi \mathfrak {g}^-\oplus \Pi \mathfrak {g}^+=\{(\psi ^-_\textrm{in},\psi ^+_\textrm{out})\}$ is the complex

13 $$\begin{aligned} \mathcal {Y}=\Omega ^\bullet (I,\{1\};\Pi \mathfrak {g}^+)\oplus \Omega ^\bullet (I,\{0\};\Pi \mathfrak {g}^-) , \end{aligned}$$

which admits the following decomposition:

14 $$\begin{aligned} \mathcal {Y}= & {} \underbrace{\big (dt\cdot \mathfrak {g}_{\mathbb {C}} \oplus (1-t)\cdot \Pi \mathfrak {g}^+\oplus t\cdot \Pi \mathfrak {g}^-\big )}_{\mathcal {V}} \nonumber \\{} & {} \bigoplus \underbrace{\big (\Omega ^0_{\int =0}(I,\{1\};\Pi \mathfrak {g}^+)\oplus \Omega ^0_{\int =0}(I,\{0\};\Pi \mathfrak {g}^-)\big )}_{\mathcal {Y}'_{K-ex}} \nonumber \\{} & {} \bigoplus \underbrace{ \begin{array}{l} \Big (\Big \{g^+(t)dt \in \Omega ^1(I;\mathfrak {g}^+)\;\Big |\; \int _I dt\,g^+(t)\cdot t=0\Big \}\oplus \\ \oplus \Big \{g^-(t)dt \in \Omega ^1(I;\mathfrak {g}^-)\;\Big |\; \int _I dt\,g^-(t)\cdot (1-t)=0\Big \} \Big ) \end{array} }_{\mathcal {Y}'_{d-ex}} \end{aligned}$$

Again, this is a splitting of $\mathcal {Y}$ into a deformation retract^19^ and an acyclic subcomplex, with the latter split in turn into the *d*-exact part and a direct complement—the *K*-exact part, with the chain homotopy *K* taking the form

$$\begin{aligned} K:\begin{array}{lcl} dt\, g^+(t) &{}\mapsto &{} -\int _t^1 dt'\, g^+(t')+ 2(1-t) \int _0^1 dt' \, t'\,g^+(t') , \\ dt\, g^-(t) &{}\mapsto &{} \int _0^t dt'\, g^-(t')-2t \int _0^1 dt' \, (1-t')\, g^-(t') . \end{array} \end{aligned}$$

Its integral kernel—the propagator—is

15 $$\begin{aligned} \eta (t,t')=\pi ^+\otimes \big (-\theta (t'-t)+2(1-t)\,t'\big )+\pi ^-\otimes \big (\theta (t-t') -2t\, (1-t')\big ) . \end{aligned}$$

We write an element of the space of residual fields $\mathcal {V}$ as $(1-t)\cdot \psi ^+_\textrm{res}+t\cdot \psi ^-_\textrm{res}+dt \cdot A_\textrm{res}$, with $\psi ^+_\textrm{res}\in \Pi \mathfrak {g}^+, \psi ^-_\textrm{res}\in \Pi \mathfrak {g}^-,A_\textrm{res}\in \mathfrak {g}_{\mathbb {C}}$.

The BV-BFV partition function is:

16 $$\begin{aligned}{} & {} Z(\psi ^-_\textrm{in},\psi ^+_\textrm{out};\psi ^+_\textrm{res},\psi ^-_\textrm{res},A_\textrm{res})\nonumber \\{} & {} \quad = \int _{\mathcal {Y}'_{K-ex}\subset \mathcal {Y}'} \mathcal {D}\psi ^+_\textrm{fl}\, \mathcal {D}\psi ^-_\textrm{fl}\; e^{\frac{i}{\hbar }S^f\left( \widetilde{\psi ^-_\textrm{in}}+\widetilde{\psi ^+_\textrm{out}}+ (1-t)\cdot \psi ^+_\textrm{res}+t\cdot \psi ^-_\textrm{res}+\psi ^+_\textrm{fl}+\psi ^-_\textrm{fl}+dt\cdot A_\textrm{res}\right) }\nonumber \\{} & {} \quad =\int \mathcal {D}\psi ^+_\textrm{fl}\, \mathcal {D}\psi ^-_\textrm{fl}\; \exp \frac{i}{\hbar }\Big ( \int (\psi ^-_\textrm{fl},d_I\psi ^+_\textrm{fl}) +\frac{1}{2} (\psi ^-_\textrm{res},\psi ^+_\textrm{res}) \nonumber \\{} & {} \qquad +\,(\psi ^+_\textrm{out},\psi ^-_\textrm{res}+\psi ^-_\textrm{fl}(1)) - (\psi ^-_\textrm{in},\psi ^+_\textrm{res}+\psi ^+_\textrm{fl}(0)) \Big )\nonumber \\{} & {} \quad =\exp {\frac{i}{\hbar }\left( \frac{1}{2} (\psi ^-_\textrm{res},\psi ^+_\textrm{res})+(\psi ^+_\textrm{out},\psi ^-_\textrm{res})-(\psi ^-_\textrm{in},\psi ^+_\textrm{res})+(\psi ^-_\textrm{in},\psi ^+_\textrm{out}) \right) } . \end{aligned}$$

Here the last term comes from the simple Feynman diagram with a single propagator connecting $\psi ^+_\textrm{out}$ and $\psi ^-_\textrm{in}$.

#### Remark 3.1

One can further integrate out $\psi ^\pm _\textrm{res}$ in (16) resulting in the partition function

17 $$\begin{aligned} Z(\psi ^-_\textrm{in},\psi ^+_\textrm{out})=e^{\frac{i}{\hbar }(\psi ^+_\textrm{out},\psi ^-_\textrm{in})}. \end{aligned}$$

It corresponds to choosing the space of residual fields in (13) to be zero (which is possible since the full complex $\mathcal {Y}$ is acyclic). Thus, (17) is the minimal realization of the partition function of the theory on the interval with prescribed boundary polarizations, and it is the BV pushforward of the nonminimal realization (16).

#### Remark 3.2

The exponent $S_\textrm{HJ}=(\psi ^+_\textrm{out},\psi ^-_\textrm{in})$ in (17) is the Hamilton–Jacobi action for the theory: it is the action (12) evaluated on the (unique) solution of EL equation $\dot{\psi }=0$ satisfying the boundary conditions $\psi ^-|_{t=0}=\psi ^-_\textrm{in}$, $\psi ^+|_{t=1}=\psi ^+_\textrm{out}$. Also, $S_\textrm{HJ}$ is the generating function for the evolution relation of the theory:

$$\begin{aligned} L_{S_\textrm{HJ}}= & {} \Big \{\psi |_{t=1}=\psi ^+_\textrm{out}+\frac{\partial S_\textrm{HJ}}{\partial \psi ^+_\textrm{out}}=\psi ^+_\textrm{out}+\psi ^-_\textrm{in}\;\; ,\;\; \psi |_{t=0}=\psi ^-_\textrm{in}- \frac{\partial S_\textrm{HJ}}{\partial \psi ^-_\textrm{in}} = \psi ^-_\textrm{in}+\psi ^+_\textrm{out}\Big \} \\= & {} \big \{ \psi |_{t=1}=\psi |_{t=0}\big \} \quad \subset \;\; \Pi \mathfrak {g}\times \Pi \mathfrak {g}. \end{aligned}$$

This provides a simple example of Hamilton–Jacobi formalism, see [14] and Sect. 2, with the phase space being the symplectic *super*manifold $\Pi \mathfrak {g}$.

Moreover, the exponent in (16) is a *generalized* generating function for the evolution relation, with $\psi ^\pm _\textrm{res}$ the auxiliary parameters. It can also be seen as the Hamilton–Jacobi action for the action $S^f+\int dt (\lambda , \psi -\frac{1}{2} \psi _\textrm{res})$ with $S^f$ as in (12) and where $\lambda \in \Pi \mathfrak {g}$ (a constant along *I*) is a Lagrange multiplier.

Likewise, the exponent $(\psi ^+_\textrm{out}-\psi ^+_\textrm{in},\psi ^-_\textrm{res})$ in the right hand side of (11) is the generalized generating function for the same evolution relation, with respect to $(\psi ^+_\textrm{out},\psi ^+_\textrm{in})$-polarization, with $\psi ^-_\textrm{res}$ the auxiliary parameter, cf. [14, Section 6.1].

To summarize, in these three cases Threorem A holds:

- Case of (17): 1D abelian Chern–Simons with $(\psi ^+_\textrm{out},\psi ^-_\textrm{in})$-polarization at the endpoints of the interval, with $\mathcal {V}=V_\textrm{aux}=0$.
- Case of (16): 1D abelian Chern–Simons with $(\psi ^+_\textrm{out},\psi ^-_\textrm{in})$-polarization, with $\mathcal {V}$parametrized by $(\psi ^+_\textrm{res},\psi ^-_\textrm{res},A_\textrm{res})\in \Pi \mathfrak {g}^+\oplus \Pi \mathfrak {g}^-\oplus \mathfrak {g}_\mathbb {C}$ and with $V_\textrm{aux}=\Pi \mathfrak {g}^+\oplus \Pi \mathfrak {g}^-$ parametrized by $(\psi ^+_\textrm{res},\psi ^-_\textrm{res})$.
- Case of (11): 1D abelian Chern–Simons with $(\psi ^+_\textrm{out},\psi ^+_\textrm{in})$-polarization, with $\mathcal {V}=\Pi \mathfrak {g}^-\oplus \mathfrak {g}^+$ parametrized by $(\psi ^-_\textrm{res},A^+_\textrm{res})$ and $V_\textrm{aux}=\Pi \mathfrak {g}^-$ parametrized by $\psi ^-_\textrm{res}$.

Note that in these cases $V_\textrm{aux}$ is a direct summand in $\mathcal {V}$ but it is not singled out by the condition of vanishing ghost number (rather, it is the *odd* part of $\mathcal {V}$): space of fields of 1D abelian Chern–Simons as considered here does not admit a $\mathbb {Z}$-grading.

We also remark that in all these cases Theorem B works trivially: $\Omega =0$ in 1D abelian Chern–Simons and $\Delta$ contains a derivative in $A_\textrm{res}$ on which $S_\textrm{eff}$ does not depend.

## BV-BFV Approach to 3D Abelian Chern–Simons on a Cylinder

Consider the 3-dimensional abelian Chern–Simons theory on a cylinder $I\times \Sigma$, with $\Sigma$ a closed oriented surface and $I=[0,1]$ the interval parametrized by the coordinate *t*. The space of BV fields, as given by the AKSZ construction, is the $\mathbb {Z}$-graded mapping space

$$\begin{aligned} \mathcal {F}= \textrm{Map}(T[1](I\times \Sigma ), \mathbb {R}[1]) =\Omega ^\bullet (I\times \Sigma )[1]. \end{aligned}$$

Exploiting the fact that the source is a cylinder, we can also write it as a free (i.e., with a quadratic action) 1-dimensional AKSZ theory with the target given by forms on $\Sigma$:

$$\begin{aligned} \mathcal {F}=\textrm{Map}(T[1]I,\textrm{Map}(T[1]\Sigma ,\mathbb {R}[1]))=\Omega ^\bullet (I,\Omega ^\bullet (\Sigma )[1]). \end{aligned}$$

The BV action is:

18 $$\begin{aligned} S=\int _{ I\times \Sigma } \frac{1}{2} \mathcal {A}\wedge d\mathcal {A}= \int _I \frac{1}{2} (\mathcal {A}, d_I\mathcal {A}) + \frac{1}{2} (\mathcal {A} ,d_\Sigma \mathcal {A}). \end{aligned}$$

Here $d=d_I+d_\Sigma$ is the de Rham operator on the cylinder splitting into the surface part and the interval part; the pairing is integration over the surface: $(u,v)=\int _\Sigma u\wedge v$. The field splits into 0- and 1-form components along *I* as

$$\begin{aligned} \mathcal {A}=\textsf{A}+dt\cdot \textsf{A}_I\end{aligned}$$

with $\textsf{A},\textsf{A}_I$ two *t*-dependent nonhomogeneous forms on $\Sigma$; their homogeneous components are prescribed internal $\mathbb {Z}$-grading (ghost number) as follows: $\textrm{gh}(\textsf{A}^{(p)})=1-p$, $\textrm{gh}(\textsf{A}_I^{(p)})=-p$.

Comparing to the discussion of Sect. 3, this theory can be seen as 1-dimensional Chern–Simons on *I* with coefficients in $\mathfrak {g}=\Omega ^\bullet (\Sigma )$. Here the fact that $\mathfrak {g}$ is itself a cochain complex with differential $d_\Sigma$ gives rise to an additional term in the action. Also, the fact that $\mathfrak {g}$ has a degree $-2$ (rather than degree 0) graded-symmetric pairing allows one to prescribe $\mathbb {Z}$-grading to fields (in such a way that the action has degree 0 and the odd symplectic form has degree $-1$) rather than just $\mathbb {Z}_2$-grading.

The BFV phase space assigned to a boundary surface ($\{1\}\times \Sigma$ or $\{0\}\times \Sigma$) is $\mathcal {F}^\partial _\Sigma =\Omega ^\bullet (\Sigma )[1]$ which is 0-symplectic, with the Noether 1-form $\pm \int _\Sigma \frac{1}{2} \textsf{A}\wedge \delta \textsf{A}$ where the sign is $+$ for the out-boundary and − for the in-boundary. The phase space carries a degree $-1$ BFV action

19 $$\begin{aligned} S_\Sigma = \pm \int _\Sigma \frac{1}{2} \textsf{A}\wedge d_\Sigma \textsf{A}. \end{aligned}$$

Next, assume that $\Sigma$ is endowed with a complex structure, so that complex-valued 1-forms split as $\Omega ^1_\mathbb {C}(\Sigma )=\Omega ^{1,0}(\Sigma )\oplus \Omega ^{0,1}(\Sigma )$. Then, mimicking (5), we split the (complexified) space of all forms on $\Sigma$ as follows:

20 $$\begin{aligned} \underbrace{\Omega ^\bullet _\mathbb {C}(\Sigma )}_{\mathfrak {g}_\mathbb {C}} = \underbrace{(\Omega ^0_\mathbb {C}(\Sigma ) \oplus \Omega ^{1,0}(\Sigma ))}_{\mathfrak {g}^+} \bigoplus \underbrace{(\Omega ^{0,1}(\Sigma )\oplus \Omega ^2_\mathbb {C}(\Sigma ))}_{\mathfrak {g}^-} . \end{aligned}$$

This is, clearly, a splitting into lagrangian subspaces.

### Holomorphic-to-holomorphic boundary conditions

Consider the polarization$\textrm{Span}\{\frac{\delta }{\delta \textsf{A}^-}\}$ on both boundary surfaces, at $t=0$ and $t=1$, i.e., the one where we prescribe boundary values $\textsf{A}^+_\textrm{in}$, $\textsf{A}^+_\textrm{out}$. The corresponding modification of the action by boundary terms adjusting for the polarization is:

$$\begin{aligned} S^f=S+\frac{1}{2}\int _{\{1\}\times \Sigma } \textsf{A}^+ \textsf{A}^- -\frac{1}{2}\int _{\{0\}\times \Sigma } \textsf{A}^+ \textsf{A}^- \end{aligned}$$

and the corresponding Noether 1-form is:

$$\begin{aligned} \alpha ^f_{\Sigma \times \partial I}=\int _{\{1\}\times \Sigma } \textsf{A}^- \delta \textsf{A}^+ - \int _{\{0\}\times \Sigma } \textsf{A}^- \delta \textsf{A}^+ . \end{aligned}$$

The fiber of the (complexified) space of fields over the space of boundary conditions $\mathcal {B}=\mathfrak {g}^+[1]\oplus \mathfrak {g}^+[1] =\{(\textsf{A}^+_\textrm{in},\textsf{A}^+_\textrm{out})\}$ is:

$$\begin{aligned} \mathcal {Y}=\Omega ^\bullet (I,\partial I;\mathfrak {g}^+[1]) \oplus \Omega ^\bullet (I; \mathfrak {g}^-[1]) . \end{aligned}$$

Hodge decomposition (7) holds (where one should replace $\Pi$ with degree shift [1]) and the formula for the chain homotopy (8) and the propagator (9) also. Writing out the projectors $\pi ^\pm$ explicitly in our case, we obtain the following formula for the propagator:

21 $$\begin{aligned}{} & {} \eta ((z,\bar{z},t)\, ;\, (z',\bar{z}',t'))\nonumber \\{} & {} \quad =\delta ^{(2)}(z-z')\;\frac{i}{2}\big (-dz\wedge d\bar{z}'+dz'\wedge d\bar{z}'\big ) \; \big (\theta (t-t')-t\big )\nonumber \\{} & {} \qquad +\,\delta ^{(2)}(z-z')\; \frac{i}{2}\big (d\bar{z}\wedge dz' + dz\wedge d\bar{z} \big )\; \big (t'-\theta (t'-t)\big ). \end{aligned}$$

—This is a distributional 2-form on $(I\times \Sigma )\times (I\times \Sigma )$. Here *z* is the local complex coordinate on $\Sigma$. Our convention for the normalization of the delta function is: $\int \frac{i}{2} dz\wedge d\bar{z}\; \delta ^{(2)}(z-z')=1$.

Note that the propagator (21) is for the $d_I$ term in the action (18) only, whereas the $d_\Sigma$ term is treated as a perturbation.

The space of residual fields is:

$$\begin{aligned} \mathcal {V}=dt\cdot \mathfrak {g}^+\oplus 1\cdot \mathfrak {g}^- = \{dt\cdot \textsf{A}^0_{I\,\textrm{res}} +dt\cdot \textsf{A}^{1,0}_{I\,\textrm{res}}+\textsf{A}^{0,1}_\textrm{res}+\textsf{A}^2_\textrm{res}\} , \end{aligned}$$

where $\textsf{A}^0_{I\,\textrm{res}},\; \textsf{A}^{1,0}_{I\,\textrm{res}},\; \textsf{A}^{0,1}_\textrm{res},\; \textsf{A}^2_\textrm{res}$ are *t*-independent forms on $\Sigma$ of de Rham degree 0, (1, 0), (0, 1), 2, respectively, with internal degree $0,-1,0,-1$, respectively.

#### Remark 4.1

(*Axial gauge*) We call the gauge fixing introduced here the *axial gauge*: it sets the “axial” field fluctuations—those which are 1-forms along *I* and forms of any degree along $\Sigma$—to zero.

On the level of homological algebra, for *M*, *N* closed manifolds, one can construct a chain contraction *K* from $\Omega ^\bullet (M\times N)$ to $H^\bullet (M)\otimes \Omega ^\bullet (N)$ of the form $K=K_M\otimes \textrm{id}_N$ with $K_M$ a chain contraction from forms on *M* to its cohomology (cohomology can be swapped for any deformation retract of the de Rham complex in the construction). The integral kernel of *K*—the propagator—is a distributional form on $(M\times N)^{\times 2}$ containing the delta form on $N\times N$. A version of the axial gauge for Chern–Simons theory was first employed in [19]. In our situation, $N=\Sigma$ and $M=I$ is not a closed manifold and hence the construction has to be adapted for boundary conditions—which is exactly what we did above. The chain contraction, corresponding to (21), has the form $K=K_{I,\partial I}\otimes \textrm{id}_{\mathfrak {g}^+}+K_I \otimes \textrm{id}_{\mathfrak {g}^-}$. We will encounter versions of this construction for different choices of boundary conditions further in this paper (e.g., in the case of Sect. 4.2, the chain contraction has the form $K_{I,\{1\}}\otimes \textrm{id}_{\mathfrak {g}^+}+K_{I,\{0\}}\otimes \textrm{id}_{\mathfrak {g}^-}$).^20^

The BV-BFV partition function is readily calculated:

22 $$\begin{aligned}{} & {} Z(\underbrace{\textsf{A}^0_\textrm{in},\textsf{A}^{1,0}_\textrm{in}}_{\textsf{A}^+_\textrm{in}},\underbrace{ \textsf{A}^0_\textrm{out},\textsf{A}^{1,0}_\textrm{out}}_{\textsf{A}^+_\textrm{out}};\underbrace{\textsf{A}^0_{I\,\textrm{res}},\textsf{A}^{1,0}_{I\,\textrm{res}}}_{\textsf{A}^+_{I\,\textrm{res}}},\underbrace{\textsf{A}^{0,1}_\textrm{res},\textsf{A}^2_\textrm{res}}_{\textsf{A}^-_\textrm{res}})\nonumber \\{} & {} \quad = \int _{\mathcal {Y}'_{K-ex}\subset \mathcal {Y}'}\mathcal {D} \textsf{A}^+_\textrm{fl}\;\mathcal {D} \textsf{A}^-_\textrm{fl}\;e^{\frac{i}{\hbar }S^f\left( \widetilde{\textsf{A}^+_\textrm{in}}+\widetilde{\textsf{A}^+_\textrm{out}}+ \textsf{A}^+_\textrm{fl}+\textsf{A}^-_\textrm{res}+\textsf{A}^-_\textrm{fl}+dt\cdot \textsf{A}^+_{I\,\textrm{res}} \right) }\nonumber \\{} & {} \quad =\int \mathcal {D}\textsf{A}^+_\textrm{fl}\;\mathcal {D} \textsf{A}^-_\textrm{fl}\;e^{\frac{i}{\hbar }\big ( \int _{ I\times \Sigma } \textsf{A}^-_\textrm{fl}d_I\textsf{A}^+_\textrm{fl}+ \int _{\{1\}\times \Sigma } \textsf{A}^+_\textrm{out}\textsf{A}^- - \int _{\{0\}\times \Sigma } \textsf{A}^+_\textrm{in}\textsf{A}^- + \int _{ I\times \Sigma } \frac{1}{2}\mathcal {A}\, d_\Sigma \mathcal {A}\big )} \nonumber \\{} & {} \quad =\int \mathcal {D} \textsf{A}^+_\textrm{fl}\;\mathcal {D} \textsf{A}^-_\textrm{fl}\; \exp \frac{i}{\hbar } \Big (\int _{ I\times \Sigma } \textsf{A}^-_\textrm{fl}\, d_I\textsf{A}^+_\textrm{fl}+ \int _{\Sigma } \textsf{A}^+_\textrm{out}\, (\textsf{A}^-_\textrm{res}+\textsf{A}^-_\textrm{fl}\big |_{t=1}) \nonumber \\{} & {} \qquad - \int _{\Sigma } \textsf{A}^+_\textrm{in}\, (\textsf{A}^-_\textrm{res}+\textsf{A}^-_\textrm{fl}\big |_{t=0}) + \int _\Sigma \textsf{A}^0_{I\,\textrm{res}} \partial \textsf{A}^{0,1}_\textrm{res}+ \int _{ I\times \Sigma } dt\; \textsf{A}^+_{I\,\textrm{res}} \,\bar{\partial }\textsf{A}^+_\textrm{fl}\Big ) . \end{aligned}$$

Here we are using the splitting $d_\Sigma =\partial +\bar{\partial }$ of de Rham operator on $\Sigma$ into the holomorphic and antiholomorphic Dolbeault operators. Finally, computing this Gaussian integral, we obtain

23 $$\begin{aligned} Z=\exp \frac{i}{\hbar }\int _\Sigma \Big ( (\textsf{A}^+_\textrm{out}- \textsf{A}^+_\textrm{in})\; \textsf{A}^-_\textrm{res}+\textsf{A}^0_{I\,\textrm{res}}\,\partial \textsf{A}^{0,1}_\textrm{res}+\frac{1}{2} (\textsf{A}^+_\textrm{out}+\textsf{A}^+_\textrm{in})\;\bar{\partial }\textsf{A}^+_{I\,\textrm{res}} \Big ) . \end{aligned}$$

Here the last term arises from the Wick contractionand a similar one with $\textsf{A}^+_\textrm{in}$ talking to $\bar{\partial }\textsf{A}^+_{I\,\textrm{res}}$.

**Fig. 1:**
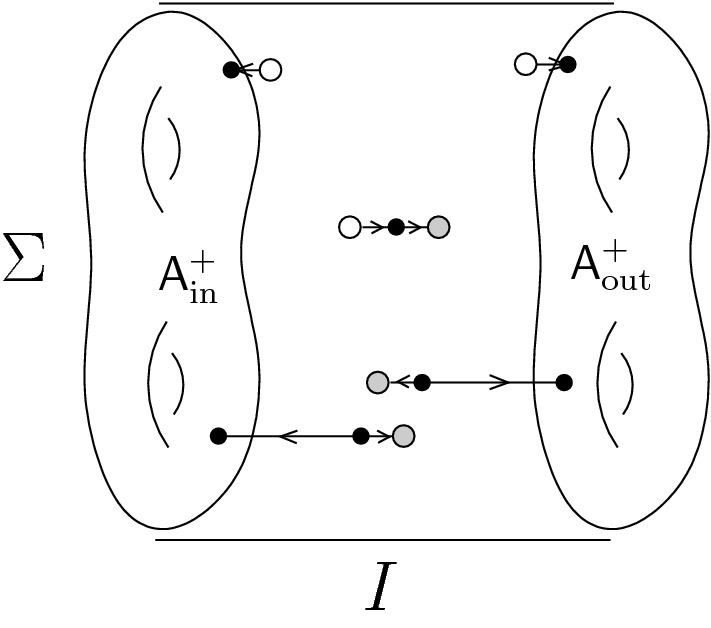
Feynman diagrams for the abelian theory on a cylinder in holomorphic-to-holomorphic polarization

Graphically, the diagrams contributing to (23) are shown in Fig. 1.

Here the conventions (Feynman rules) are:

- Black dots are vertices, which can be on in- or out-boundary (then they are univalent, with single incoming half-edge), or in the bulk (then they are bivalent – with one incoming and one outgoing, or with two outgoing half-edges).
- Half-edges can be internal (joined into pairs forming an edge, depicted as a long edge above) or external – depicted as a short edge ending with a white or a gray blob, depending on orientation.
- Boundary vertices are decorated by $\textsf{A}^+_\textrm{out}$ on the out-boundary and by $\textsf{A}^+_\textrm{in}$ on the in-boundary.
- White blobs are decorated by $\textsf{A}^-_\textrm{res}$, gray blobs are decorated by $\textsf{A}^+_{I\,\textrm{res}}$.
- (Long) edges are decorated by the propagator $\eta$.
- Bulk vertices with one incoming and one outgoing half-edge carry $\partial$; bulk vertices with two outgoing half-edges carry $\bar{\partial }$. (Equivalently, one may say that bulk vertices are decorated by $d_\Sigma$ independently of orientation.)
- For each connected graph $\Gamma$ in Figure 1, we take the product of decorations obtaining a differential form on $\textrm{Conf}_\Gamma =\Sigma ^{\#\mathrm {in-vertices}}\times (I\times \Sigma )^{\#\mathrm {bulk\,vertices}}\times \Sigma ^{\#\mathrm {out-vertices}}$ depending on the residual fields. Then we take integral over $\textrm{Conf}_\Gamma$, obtaining the value of the diagram.

We will return to the version of the result (23) in the context of *nonabelian* Chern–Simons theory in Sect. 5.4.

#### Comparison with Hamilton–Jacobi action

We can write the result (23) in the form

24 $$\begin{aligned} Z= & {} \exp \frac{i}{\hbar }\Big ( \underbrace{\int _\Sigma \Big ( (\textsf{A}^{1,0}_\textrm{out}-\textsf{A}^{1,0}_\textrm{in})\lambda +\lambda \partial \sigma + \frac{1}{2} (\textsf{A}^{1,0}_\textrm{out}+\textsf{A}^{1,0}_\textrm{in})\bar{\partial }\sigma \Big ) }_{S_\text {HJ}} \nonumber \\{} & {} +\, \int _\Sigma \Big ( (\textsf{A}^0_\textrm{out}-\textsf{A}^0_\textrm{in})\textsf{A}^2_\textrm{res}+\frac{1}{2}(\textsf{A}^0_\textrm{out}+\textsf{A}^0_\textrm{in})\bar{\partial }\textsf{A}^{1,0}_{I\,\textrm{res}}\Big ) \Big ) , \end{aligned}$$

where we introduced the alternative notation for degree zero residual fields

$$\begin{aligned} \lambda := \textsf{A}^{0,1}_\textrm{res},\;\; \sigma :=\textsf{A}^0_{I\,\textrm{res}}. \end{aligned}$$

In the first integral in (24) we recognize the Hamilton–Jacobi action [14, Eq. (48)], which can be seen as the conformal $\beta \gamma$-system coupled to the boundary fields, while in the second integral we collected the contribution of nonzero-degree fields.

#### Quantum master equation

The space of states on a surface with $\textsf{A}^+$-fixed polarization is the space of functions of $\textsf{A}^+$ of the form

25 $$\begin{aligned} \Psi (\textsf{A}^+)=\sum _{n\ge 0} \int _{\Sigma ^n}\gamma _n \pi _1^* \textsf{A}^+\cdots \pi _n^* \textsf{A}^+ \end{aligned}$$

where $\{\gamma _n\}$ are $\hbar$-dependent distributional forms on $\Sigma ^n$ and $\pi _i:\Sigma ^n\rightarrow \Sigma$ is the projection to the *i*-th copy of $\Sigma$ (we refer to Cattaneo et al. [13, Section 3.5.1] for details). The space of states is equipped with the differential (the quantum BFV operator)

26 $$\begin{aligned} \Omega _\Sigma ^+=\int _\Sigma \left( -i\hbar \, \partial \textsf{A}^0\,\frac{\delta }{\delta \textsf{A}^{1,0}}+\epsilon \textsf{A}^{1,0}\,\bar{\partial }\textsf{A}^0 \right) \end{aligned}$$

with the sign $\epsilon =+1$ for the out-boundary and $\epsilon =-1$ for the in-boundary^21^; the superscript in $\Omega ^+_\Sigma$ is a reminder of the choice of polarization. This operator is the canonical quantization of the boundary BFV action (19),

27 $$\begin{aligned} S_\Sigma =\epsilon \int _\Sigma \textsf{A}^{1,0}\,\bar{\partial }\textsf{A}^0+ \textsf{A}^{0,1}\,\partial \textsf{A}^0 . \end{aligned}$$

In the quantization, $\textsf{A}^0, \textsf{A}^{1,0}$ become multiplication operators and $\textsf{A}^{0,1}\mapsto -\epsilon \, i\hbar \frac{\delta }{\delta \textsf{A}^{1,0}}$, $\textsf{A}^2\mapsto -\epsilon \, i\hbar \frac{\delta }{\delta \textsf{A}^0}$ become derivations.

##### Lemma 4.2

The partition function (23) satisfies the BV quantum master equation modified by the boundary terms (see [13]):

28 $$\begin{aligned}{} & {} \Big ( \underbrace{ \int _\Sigma \big (-i\hbar \, \partial \textsf{A}^0_\textrm{out}\,\frac{\delta }{\delta \textsf{A}^{1,0}_\textrm{out}}+\textsf{A}^{1,0}_\textrm{out}\,\bar{\partial }\textsf{A}^0_\textrm{out}\big ) }_{\Omega _\textrm{out}^+}+ \underbrace{ \int _\Sigma \big (-i\hbar \, \partial \textsf{A}^0_\textrm{in}\,\frac{\delta }{\delta \textsf{A}^{1,0}_\textrm{in}}-\textsf{A}^{1,0}_\textrm{in}\,\bar{\partial }\textsf{A}^0_\textrm{in}\big ) }_{\Omega _\textrm{in}^+} \nonumber \\{} & {} \qquad -\,\hbar ^2 \underbrace{\int _\Sigma \frac{\delta }{\delta \textsf{A}^-_\textrm{res}}\;\frac{\delta }{\delta \textsf{A}^+_{I\,\textrm{res}}} }_{\Delta _\textrm{res}} \Big )\; Z = 0 . \end{aligned}$$

##### Proof

One checks this by a direct computation:

29 $$\begin{aligned}{} & {} (\Omega _\textrm{out}^+ +\Omega _\textrm{in}^+)Z \nonumber \\{} & {} \quad = Z\cdot \int _\Sigma \Big ((\partial \textsf{A}^0_\textrm{out}- \partial \textsf{A}^0_\textrm{in})\,\textsf{A}^{0,1}_\textrm{res}+\frac{1}{2} (\partial \textsf{A}^0_\textrm{out}+\partial \textsf{A}^0_\textrm{in})\,\bar{\partial }\textsf{A}^0_{I\,\textrm{res}}+ \textsf{A}^{1,0}_\textrm{out}\,\bar{\partial }\textsf{A}^0_\textrm{out}-\textsf{A}^{1,0}_\textrm{in}\,\bar{\partial }\textsf{A}^0_\textrm{in}\Big ) .\nonumber \\ \end{aligned}$$

On the other hand,

30 $$\begin{aligned} \hbar ^2\Delta _\textrm{res}Z= Z\cdot \int _\Sigma \big (\textsf{A}^+_\textrm{out}-\textsf{A}^+_\textrm{in}-\partial \textsf{A}^+_{I\,\textrm{res}}\big )\,\big (\partial \textsf{A}^-_\textrm{res}+\frac{1}{2} \bar{\partial }(\textsf{A}^+_\textrm{out}+\textsf{A}^+_\textrm{in})\big ) . \end{aligned}$$

Inspecting this expression, we see that it coincides with (29), which proves (28). $\square$

Following the terminology of [13], we call the equation $(\Omega _\partial -\hbar ^2\Delta _\textrm{res}) Z=0$ the *modified* (by the boundary term) quantum master equation (mQME).

### Antiholomorphic-to-holomorphic boundary conditions

Consider the polarization $\textrm{Span}\{\frac{\delta }{\delta \textsf{A}^+}\}$ at $t=0$ and $\textrm{Span}\{\frac{\delta }{\delta \textsf{A}^-}\}$ at $t=1$. I.e., we prescribe boundary values $\textsf{A}^-_\textrm{in}, \textsf{A}^+_\textrm{out}$. The corresponding modification of the action by boundary terms adjusting for the polarization is:

$$\begin{aligned} S^f=S+\frac{1}{2}\int _{\{1\}\times \Sigma }\textsf{A}^+\textsf{A}^- + \frac{1}{2} \int _{ \{0\}\times \Sigma }\textsf{A}^+\textsf{A}^- \end{aligned}$$

and the modified boundary Noether 1-form is:

$$\begin{aligned} \alpha ^f_{\Sigma \times \partial I} = \int _{\{1\}\times \Sigma } \textsf{A}^- \delta \textsf{A}^+ -\int _{\{0\}\times \Sigma } \textsf{A}^+\delta \textsf{A}^- . \end{aligned}$$

The fiber of the (complexified) space of fields over the space of boundary conditions $\mathcal {B}=\mathfrak {g}^-[1]\oplus \mathfrak {g}^+[1]=\{(\textsf{A}^-_\textrm{in},\textsf{A}^+_\textrm{out})\}$ is the complex

$$\begin{aligned} \mathcal {Y}= \Omega ^\bullet (I,\{1\};\mathfrak {g}^+[1])\oplus \Omega ^\bullet (I,\{0\};\mathfrak {g}^-[1]) . \end{aligned}$$

Hodge decomposition (14) holds (where one replaces $\Pi \rightarrow [1]$) and the propagator is given by (15) or, more explicitly,

$$\begin{aligned}{} & {} \eta ((z,\bar{z},t)\, ; \, (z',\bar{z}',t'))=\delta ^{(2)}(z-z')\frac{i}{2}(-dz\wedge d\bar{z}'+dz'\wedge d\bar{z}')\;\big (-\theta (t'-t)+2(1-t)\, t'\big )\\{} & {} \qquad +\,\delta ^{(2)}(z-z')\frac{i}{2}(d\bar{z}\wedge dz'+ dz\wedge d\bar{z})\;\big (\theta (t-t')-2t\,(1-t')\big ) . \end{aligned}$$

The space of residual fields is:

31 $$\begin{aligned} \mathcal {V}=dt\cdot \mathfrak {g}_{\mathbb {C}}\oplus (1-t)\cdot \mathfrak {g}^+[1] \oplus t\cdot \mathfrak {g}^-[1] = \{dt\cdot \textsf{A}_{I\,\textrm{res}}+ (1-t)\cdot \textsf{A}^+_\textrm{res}+ t\cdot \textsf{A}^-_\textrm{res}\},\nonumber \\ \end{aligned}$$

where $\textsf{A}_{I\,\textrm{res}}$, $\textsf{A}^+_\textrm{res}$, $\textsf{A}^-_\textrm{res}$ are *t*-independent forms on $\Sigma$. The homogeneous components of these residual fields and their internal degrees (ghost numbers) are as follows:

$$\begin{aligned} \begin{array}{lllll|lll|lll} \textsf{A}_{{I\,\textrm{res}}}= &{} \textsf{A}_{I\,\textrm{res}}^0+ &{} \textsf{A}_{I\,\textrm{res}}^{1,0}+ &{} \textsf{A}_{I\,\textrm{res}}^{0,1}+ &{} \textsf{A}_{I\,\textrm{res}}^2 &{} \textsf{A}^+_\textrm{res}= &{} \textsf{A}^0_\textrm{res}+ &{} \textsf{A}^{1,0}_\textrm{res}&{} \textsf{A}^-_\textrm{res}= &{} \textsf{A}^{0,1}_\textrm{res}+ &{} \textsf{A}^2_\textrm{res}\\ &{} 0 &{} -1 &{} -1 &{} -2 &{} &{} 1 &{} 0 &{} &{} 0 &{} -1 \end{array} \end{aligned}$$

The BV-BFV partition function is:

32 $$\begin{aligned}{} & {} Z(\textsf{A}^-_\textrm{in},\textsf{A}^+_\textrm{out};\textsf{A}_{I\,\textrm{res}}, \textsf{A}^+_\textrm{res},\textsf{A}^-_\textrm{res})\nonumber \\{} & {} \quad =\int _{\mathcal {Y}'_{K-ex}\subset \mathcal {Y}'} \mathcal {D}\textsf{A}^+_\textrm{fl}\,\mathcal {D}\textsf{A}^-_\textrm{fl}\; e^{\frac{i}{\hbar }S^f\Big (\textsf{A}^-_\textrm{in}+\textsf{A}^+_\textrm{out}+(1-t)\cdot \textsf{A}^+_\textrm{res}+t\cdot \textsf{A}^-_\textrm{res}+\textsf{A}^+_\textrm{fl}+\textsf{A}^-_\textrm{fl}+dt\cdot \textsf{A}_{I\,\textrm{res}}\Big )}\nonumber \\{} & {} \quad =\int \mathcal {D}\textsf{A}^+_\textrm{fl}\,\mathcal {D}\textsf{A}^-_\textrm{fl}\; e^\frac{i}{\hbar }\Big (\int _{ I\times \Sigma } \big (\textsf{A}^-_\textrm{fl}+t \textsf{A}^-_\textrm{res}\big )\,d_I\big (\textsf{A}^+_\textrm{fl}+(1-t)\textsf{A}^+_\textrm{res}\big )\nonumber \\{} & {} \qquad +\int _{\{1\}\times \Sigma } \textsf{A}^+_\textrm{out}\textsf{A}^- - \int _{\{0\}\times \Sigma } \textsf{A}^-_\textrm{in}\textsf{A}^+ +\int _{ I\times \Sigma } \frac{1}{2} \mathcal {A}\,d_\Sigma \mathcal {A}\Big )\nonumber \\{} & {} \quad =\int \mathcal {D}\textsf{A}^+_\textrm{fl}\,\mathcal {D}\textsf{A}^-_\textrm{fl}\; \exp \frac{i}{\hbar } \Big ( \int _{ I\times \Sigma } \textsf{A}^-_\textrm{fl}\, d_I\textsf{A}^+_\textrm{fl}+ \frac{1}{2} \int _\Sigma \textsf{A}^-_\textrm{res}\textsf{A}^+_\textrm{res}+\int _\Sigma \textsf{A}^+_\textrm{out}(\textsf{A}^-_\textrm{res}+\textsf{A}^-_\textrm{fl}\big |_{t=1}) \nonumber \\{} & {} \qquad -\, \int _\Sigma \textsf{A}^-_\textrm{in}(\textsf{A}^+_\textrm{res}+\textsf{A}^+_\textrm{fl}\big |_{t=0}) +\frac{1}{2} \int _\Sigma \textsf{A}_{I\,\textrm{res}}\; d_\Sigma (\textsf{A}^+_\textrm{res}+\textsf{A}^-_\textrm{res}) \Big ) \nonumber \\{} & {} \quad =\exp \frac{i}{\hbar }\int _\Sigma \Big ( -\textsf{A}^+_\textrm{out}\textsf{A}^-_\textrm{in}+\textsf{A}^+_\textrm{out}\textsf{A}^-_\textrm{res}-\textsf{A}^-_\textrm{in}\textsf{A}^+_\textrm{res}+\frac{1}{2} \textsf{A}^-_\textrm{res}\textsf{A}^+_\textrm{res}\nonumber \\{} & {} \qquad +\,\frac{1}{2} \big ( \textsf{A}^{0,1}_{I\,\textrm{res}}\partial \textsf{A}^0_\textrm{res}+ \textsf{A}^{1,0}_{I\,\textrm{res}}\bar{\partial }\textsf{A}^0_\textrm{res}+ \textsf{A}^0_{I\,\textrm{res}}(\partial \textsf{A}^{0,1}_\textrm{res}+ \bar{\partial }\textsf{A}^{1,0}_\textrm{res}) \big ) \Big ) . \end{aligned}$$

Here the first term in the final result is a contribution of the diagram where $\textsf{A}^+_\textrm{out}$ is contracted by a propagator with $\textsf{A}^-_\textrm{in}$.

#### Partial integral over residual fields and comparison with Hamilton–Jacobi action

Motivated by comparison with the Hamilton–Jacobi formalism, we consider the BV pushforward of the partition function (32) along the odd symplectic fibration

$$\begin{aligned} p:\mathcal {V}\rightarrow \mathcal {V}_\textrm{small}=\{dt\cdot (\textsf{A}^0_{I\,\textrm{res}}+\textsf{A}^2_{I\,\textrm{res}})+(1-t)\cdot \textsf{A}^0_\textrm{res}+ t\cdot \textsf{A}^2_\textrm{res}\} . \end{aligned}$$

In its kernel, we choose the gauge-fixing lagrangian subspace $\mathcal {L}$ cut out by equations $\textsf{A}^{1,0}_{I\,\textrm{res}}=\textsf{A}^{0,1}_{I\,\textrm{res}}=0$ and parametrized by $\textsf{A}^{1,0}_\textrm{res}, \textsf{A}^{0,1}_\textrm{res}$. The corresponding BV pushforward is:

33 $$\begin{aligned} Z_\textrm{small}= & {} \int \mathcal {D}\textsf{A}^{1,0}_\textrm{res}\,\mathcal {D}\textsf{A}^{0,1}_\textrm{res}\; Z \nonumber \\= & {} \exp \frac{i}{\hbar } \Big (\underbrace{\int _\Sigma \Big ( \textsf{A}^{1,0}_\textrm{out}\textsf{A}^{0,1}_\textrm{in}+(\partial \textsf{A}^{0,1}_\textrm{in}+\bar{\partial }\textsf{A}^{1,0}_\textrm{out})\; \sigma +\frac{1}{2} \sigma \partial \bar{\partial }\sigma \Big )}_{S_\text {HJ}}\nonumber \\{} & {} \quad +\, \int _\Sigma \Big (-\textsf{A}^0_\textrm{out}\textsf{A}^2_\textrm{in}+\textsf{A}^0_\textrm{out}\textsf{A}^2_\textrm{res}-\textsf{A}^2_\textrm{in}\textsf{A}^0_\textrm{res}+ \frac{1}{2} \textsf{A}^2_\textrm{res}\textsf{A}^0_\textrm{res}\Big ) \Big ) . \end{aligned}$$

Here we denoted the degree zero scalar residual field by

$$\begin{aligned} \sigma :=\textsf{A}^0_{I\,\textrm{res}}\;\; \in \Omega ^0_{\mathbb {C}}(\Sigma ) . \end{aligned}$$

In the first bracket in (33) we recognize the Hamilton–Jacobi action [14, Eq. (47)] (see also Example 2.2)—the action of a free (conformal) massless boson interacting with the boundary fields,^22^ while in the second bracket we collected the contributions of nonzero-degree fields.

#### Full integral over residual fields

If we wish to integrate out the remaining residual fields completely, we construct the gauge-fixing lagrangian $\mathcal {L}_\textrm{small}\subset \mathcal {V}_\textrm{small}$ as follows. Choose an area form $\mu$ on $\Sigma$. Consider the splitting of 0-forms into constants and forms with vanishing integral against $\mu$: $\textsf{A}^0=\textsf{A}^0_\textrm{c}+\underline{\textsf{A}^0}$. Also, consider the splitting of 2-forms into constant multiples of $\mu$ and forms of vanishing total integral: $\textsf{A}^2=\mu \cdot \textsf{A}^2_\textrm{c}+\underline{\textsf{A}^2}$. Then, we define the lagrangian $\mathcal {L}_\textrm{small}\subset \mathcal {V}_\textrm{small}$ by equations $\textsf{A}^2_{I\,\textrm{res}}= \sigma _{\textrm{c}}=\underline{\textsf{A}^2_\textrm{res}}=0$. Thus, the lagrangian is parametrized by $\textsf{A}^0_\textrm{res}$, $\underline{\sigma }$, $\textsf{A}^2_{\textrm{res},\textrm{c}}$.^23^

The resulting full BV integral is:

$$\begin{aligned} Z_*= & {} \int _{ \mathcal {L}_\textrm{small}\subset \mathcal {V}_\textrm{small} } \mathcal {D}\textsf{A}^0_\textrm{res}\; \mathcal {D}\underline{\sigma }\;\mathcal {D}\textsf{A}^2_{\textrm{res},\textrm{c}}\;\; \\ Z_\textrm{small}= & {} \delta (\underline{\textsf{A}^2_\textrm{in}})e^{-\frac{i}{\hbar }\int _\Sigma \mu \cdot \textsf{A}^2_{\textrm{in},\textrm{c}}\textsf{A}^0_{\textrm{out},\textrm{c}} } \int \mathcal {D}\underline{\sigma }\; \exp \frac{i}{\hbar }\\ {}{} & {} \times \int _\Sigma \Big ( \textsf{A}^{1,0}_\textrm{out}\textsf{A}^{0,1}_\textrm{in}+(\partial \textsf{A}^{0,1}_\textrm{in}+\bar{\partial }\textsf{A}^{1,0}_\textrm{out})\; \underline{\sigma } +\frac{1}{2} \underline{\sigma } \partial \bar{\partial }\underline{\sigma } \Big ) . \end{aligned}$$

Further, assume that the area form $\mu = \sqrt{\det {g}}\; d^2x$ is the Riemannian area form associated to a certain metric *g* on $\Sigma$ inducing simultaneously the complex structure we use in our polarization. Then the integral over $\underline{\sigma }$ evaluates finally to

34 $$\begin{aligned} Z_*= \delta (\underline{\textsf{A}^2_\textrm{in}})\; e^{-\frac{i}{\hbar }\int _\Sigma \mu \cdot \textsf{A}^2_{\textrm{in},\textrm{c}}\textsf{A}^0_{\textrm{out},\textrm{c}} } \; \cdot \big ({\det }'_{\Omega ^0(\Sigma )}\Delta _g\big )^{-\frac{1}{2}}\cdot e^{\frac{i}{\hbar } \mathbb {I}(\textsf{A}^{1,0}_\textrm{out},\textsf{A}^{0,1}_\textrm{in}) } \end{aligned}$$

where

- $\Delta _g$ is the metric Laplace operator acting on 0-forms, ${\det }'$ means the zeta-regularized product of *nonzero* eigenvalues.
- The exponent in (34) is
  35 $$\begin{aligned} \mathbb {I}= & {} \int _\Sigma \textsf{A}^{1,0}_\textrm{out}P_\textrm{harm}( \textsf{A}^{0,1}_\textrm{in})\nonumber \\{} & {} -i\int _{\Sigma \times \Sigma \;\ni (z,z')} \bar{\partial }\textsf{A}^{1,0}_\textrm{out}\big |_z G(z,z')\, \bar{\partial }\textsf{A}^{1,0}_\textrm{out}\big |_{z'} + \partial \textsf{A}^{0,1}_\textrm{in}\big |_z G(z,z')\, \partial \textsf{A}^{0,1}_\textrm{in}\big |_{z'} . \end{aligned}$$
  Here *G* is the Green’s function for $\Delta _g$, viewed as a function on $\Sigma \times \Sigma$ with a logarithmic singularity at the diagonal.^24^ The operator $P_\textrm{harm}: \textsf{A}^{0,1}\mapsto \textsf{A}^{0,1}- 2i\int _{\Sigma \ni z'} \bar{\partial }G(z,z') \partial \textsf{A}^{0,1}\big |_{z'}$ is the projector onto harmonic (0, 1)-forms in the Hodge decomposition.

Written in different notations, the exponent in (34) is:

36 $$\begin{aligned} \qquad \mathbb {I}\!=\!\int _\Sigma \textsf{A}^{1,0}_\textrm{out}\, (1-\bar{\partial }(\partial \bar{\partial })^{-1}\partial ) \,\textsf{A}^{0,1}_\textrm{in}\!-\!\frac{1}{2} \textsf{A}^{1,0}_\textrm{out}\, \bar{\partial }(\partial \bar{\partial })^{-1} \bar{\partial }\, \textsf{A}^{1,0}_\textrm{out}-\frac{1}{2} \textsf{A}^{0,1}_\textrm{in}\, \partial (\partial \bar{\partial })^{-1} \partial \, \textsf{A}^{0,1}_\textrm{in}.\nonumber \\ \end{aligned}$$

##### Remark 4.3

- The exponent $\mathbb {I}$ in (34) depends only on the complex structure on $\Sigma$, not on the particular compatible metric *g*. In other words, it is invariant under Weyl transformations of the metric $g\mapsto e^{\phi }\, g$. Weyl-invariance of $\mathbb {I}$ is manifest in the form (36).
- Unlike $\mathbb {I}$, the full quantum answer (34) is not Weyl-invariant, since the determinant of the Laplacian is not invariant (a phenomenon known as the “conformal anomaly" or “trace anomaly” of the free scalar field as a conformal field theory). In addition to that quantum effect, the dependence of $Z_*$ on boundary $\textrm{gh}\ne 0$ fields $\textsf{A}_\textrm{in}^2, \textsf{A}_\textrm{out}^0$ involves the metric area form $\mu$.
- The lagrangian generated by $\mathbb {I}$ is
  $$\begin{aligned} L_\mathbb {I}= & {} \left\{ \begin{array}{c} \textsf{A}_\textrm{out}= \textsf{A}_\textrm{out}^{1,0} + \frac{\delta \mathbb {I}}{\delta \textsf{A}^{1,0}_\textrm{out}},\\ \textsf{A}_\textrm{in}= \textsf{A}_\textrm{in}^{0,1} - \frac{\delta \mathbb {I}}{\delta \textsf{A}^{0,1}_\textrm{in}} \end{array} \right\} = \left\{ \begin{array}{c} \textsf{A}_\textrm{out}=(1-\bar{\partial }(\partial \bar{\partial })^{-1}\bar{\partial }) \textsf{A}^{1,0}_\textrm{out}+ P_\textrm{harm} \textsf{A}^{0,1}_\textrm{in},\\ \textsf{A}_\textrm{in}=(1+\partial (\partial \bar{\partial })^{-1}\partial ) \textsf{A}^{0,1}_\textrm{in}+ P_\textrm{harm} \textsf{A}^{1,0}_\textrm{out}\end{array} \right\} . \end{aligned}$$
  It is easy to see that this lagrangian coincides with the evolution relation of abelian Chern–Simons theory on the cylinder $I\times \Sigma$,
  $$\begin{aligned} L_{CS}= & {} \big \{(\textsf{A}_\textrm{out},\textsf{A}_\textrm{in})\; \in \Omega ^1(\Sigma )\times \Omega ^1(\Sigma )\;\;\big | \;\; d\textsf{A}_\textrm{out}\\ {}= & {} 0,\; d\textsf{A}_\textrm{in}=0,\; \textsf{A}_\textrm{out}-\textsf{A}_\textrm{in}=d(\cdots )\big \} . \end{aligned}$$
  Thus, $\mathbb {I}$ is a (nongeneralized^25^) Hamilton–Jacobi action for the abelian theory on the cylinder.
- Classically, one can obtain $\mathbb {I}$ from the generalized Hamilton–Jacobi action (Example 2.2) as the conditional extremum of $S_\text {HJ}$ in $\sigma$, with $\textsf{A}^{1,0}_\textrm{out}$ and $\textsf{A}^{0,1}_\textrm{in}$ fixed.

##### Remark 4.4

To make (4.3) of Remark 4.3 above more explicit: if $g_\tau =e^{\phi _\tau }g_0$ is a $\tau$-dependent family of metrics compatible with the given complex structure on $\Sigma$, one has

37 $$\begin{aligned} \frac{d}{d\tau } Z_*^{g_\tau } = (\Omega ^+_\textrm{out}+\Omega ^-_\textrm{in})(\xi Z_*^{g_\tau })+Z_*^{g_\tau }\cdot \frac{1}{48\pi }\int _\Sigma \mu _{g_\tau } R_{g_\tau }\dot{\phi }_\tau \end{aligned}$$

with *R* the scalar curvature of the metric, $\mu _{g_\tau }$ the Riemannian area form of $g_\tau$, the $\Omega$ operators given by (40), (41) below and^26^$\xi =\textsf{A}_{\textrm{in},c} \int _{\Sigma \times \Sigma }(\bar{\partial }A^{1,0}_\textrm{out}+ \partial A^{0,1}_\textrm{in})_zG(z,z')\dot{\mu }_{z'}$. The second term in (37) is the trace anomaly. Furthermore, one can compensate the anomaly term by including the Liouville action as a counterterm,^27^ i.e., by introducing

$$\begin{aligned} \widehat{Z}^{g}=Z_*^g\cdot e^{-\frac{1}{48\pi }\int _\Sigma \frac{1}{2} d\phi \wedge * d\phi +R_g\phi \mu _g }, \end{aligned}$$

where $\phi$ is defined by $g=e^{\phi }g_0$ with $g_0$ some “reference” metric in the same conformal class (e.g., one can take $g_0$ to be the uniformization metric on $\Sigma$—spherical, flat or hyperbolic metric for $\Sigma$ of genus 0, 1 or $\ge 2$, respectively). Then, for a conformal variation of metric we have $\delta _{\varphi } \widehat{Z}^{e^\varphi g} =(\Omega ^+_\textrm{out}+\Omega ^-_\textrm{in})(\xi Z_*^g)$.

As an aside, it is tempting to compare the two phenomena:

(i) The anomalous metric dependence (under a Weyl transformation $g_\Sigma \rightarrow e^\phi g_\Sigma$) of the partition function on the cylinder and the cancellation of that dependence by a Liouville action counterterm.
(ii) The anomalous metric dependence (under $g_M\rightarrow g_M+\delta g_M$) of the perturbative Chern–Simons partition function on a closed 3-manifold *M* and the cancellation of that dependence by the gravitational Chern–Simons counterterm introducing the dependence on framing *M*, see [5, 37].

But in fact, these effects seem different. In particular, the dependence on Weyl transformations in (4.4) rescales *Z* by a real factor, whereas the anomalous metric dependence in (4.4) affects only the phase of the partition function.

##### Remark 4.5

As implied by (33), one can view the “physical part” $Z_*^\textrm{ph}=e^{\frac{i}{\hbar }\mathbb {I}(\textsf{A}^{1,0}_\textrm{out},\textsf{A}^{0,1}_\textrm{in}) }$ of (34) as a generating function for the correlators of chiral currents $j=i\partial \phi$, $\bar{j}=i\bar{\partial }\phi$ in massless scalar theory (viewed as abelian WZW model):

38 $$\begin{aligned}{} & {} \langle j(z_1)\cdots j(z_n)\bar{j}(w_1)\cdots \bar{j}(w_m) \rangle \nonumber \\{} & {} \quad =\hbar ^{n+m} \frac{1}{ Z^\textrm{ph}_*}\left. \frac{\delta }{\delta \textsf{A}^{0,1}_\textrm{in}(z_1)}\cdots \frac{\delta }{\delta \textsf{A}^{0,1}_\textrm{in}(z_n)} \frac{\delta }{\delta \textsf{A}^{1,0}_\textrm{out}(w_1)}\cdots \frac{\delta }{\delta \textsf{A}^{1,0}_\textrm{out}(w_m)} \right| _{\textsf{A}^{0,1}_\textrm{in}=\textsf{A}^{1,0}_\textrm{out}=0} Z^\textrm{ph}_*\nonumber \\ \end{aligned}$$

Here we are assuming that all points $z_1,\ldots ,z_n,w_1,\ldots ,w_m$ in $\Sigma$ are pairwise distinct [so that we can ignore the term $\int _\Sigma \textsf{A}^{1,0}_\textrm{out}\textsf{A}^{0,1}_\textrm{in}$ in (33)]. We note that (36) implies a short-distance behavior of these correlators consistent with the OPEs (operator product expansions)

39 $$\begin{aligned} j(z) j(w)\sim \frac{1}{(z-w)^2}+\textrm{reg},\quad \bar{j}(z)\bar{j}(w)\sim \frac{1}{(\bar{z}-\bar{w})^2}+\textrm{reg},\quad j(z)\bar{j}(w)\sim \textrm{reg}\qquad \end{aligned}$$

as $z\rightarrow w$ (“reg” stands for the regular part)—the standard fundamental OPEs of abelian WZW model.

#### Quantum master equation

The space of states on the out-surface with $\textsf{A}^+$-fixed polarization was discussed in Sect. 4.1.2: it is the space of functions of $\textsf{A}^+_\textrm{out}$ of the form (25) with the BFV operator

40 $$\begin{aligned} \Omega _\textrm{out}^+ = \int _\Sigma \left( -i\hbar \; \partial \textsf{A}^0_\textrm{out}\frac{\delta }{\delta \textsf{A}^{1,0}_\textrm{out}}+\textsf{A}^{1,0}_\textrm{out}\,\bar{\partial }\textsf{A}^0_\textrm{out}\right) . \end{aligned}$$

The space of states on the in-surface with $\textsf{A}^-$-fixed polarization is the space of functions of $\textsf{A}^-_\textrm{in}$ [defined similarly to (25)] with the BFV operator

41 $$\begin{aligned} \Omega _\textrm{in}^- =\int _\Sigma \Big ( -i\hbar \; \partial \textsf{A}^{0,1}_\textrm{in}\frac{\delta }{\delta \textsf{A}^2_\textrm{in}}+\hbar ^2\frac{\delta }{\delta \textsf{A}^{0,1}_\textrm{in}}\,\bar{\partial }\frac{\delta }{\delta \textsf{A}^2_\textrm{in}} \Big ) . \end{aligned}$$

This is the quantization of the BFV action (27) where $\textsf{A}^{0,1},\textsf{A}^2$ become multiplication operators and $\textsf{A}^{1,0}\mapsto -\epsilon \, i\hbar \frac{\delta }{\delta \textsf{A}^{0,1}}$, $\textsf{A}^0\mapsto -\epsilon \, i\hbar \frac{\delta }{\delta \textsf{A}^2}$ become derivations, where $\epsilon =-1$ for the in-boundary, as in Sect. 4.1.2.

The BV Laplacian on residual fields (31) is^28^:

$$\begin{aligned} \Delta _\textrm{res}=2\int _\Sigma \frac{\delta }{\delta \textsf{A}^-_\textrm{res}} \frac{\delta }{\delta \textsf{A}^+_{I\,\textrm{res}}}+ \frac{\delta }{\delta \textsf{A}^+_\textrm{res}} \frac{\delta }{\delta \textsf{A}^-_{I\,\textrm{res}}} . \end{aligned}$$

##### Lemma 4.6

Partition function (32) satisfies the modified quantum master equation

$$\begin{aligned} \left( \Omega ^+_\textrm{out}+ \Omega ^-_\textrm{in}-\hbar ^2 \Delta _\textrm{res}\right) \;Z =0 . \end{aligned}$$

##### Proof

Indeed, a straightforward computation gives:

$$\begin{aligned} \Omega ^+_\textrm{out}Z&=Z\cdot \int _\Sigma \Big ( -\partial \textsf{A}^0_\textrm{out}\,(\textsf{A}^{0,1}_\textrm{in}-\textsf{A}^{0,1}_\textrm{res})+ \textsf{A}^{1,0}_\textrm{out}\;\bar{\partial }\textsf{A}^0_\textrm{out}\Big ), \\ \Omega ^-_\textrm{in}Z&=Z\cdot \int _\Sigma \Big ( \partial \textsf{A}^{0,1}_\textrm{in}\, (\textsf{A}^0_\textrm{out}-\textsf{A}^0_\textrm{res})-(\textsf{A}^{1,0}_\textrm{out}-\textsf{A}^{1,0}_\textrm{res})\;\bar{\partial }(\textsf{A}^0_\textrm{out}-\textsf{A}^0_\textrm{res}) \Big ), \\ -\hbar ^2 \Delta _\textrm{res}Z&= Z\cdot \int _\Sigma \Big ( (\partial \textsf{A}^{0,1}_\textrm{res}+\bar{\partial }\textsf{A}^{1,0}_\textrm{res})\, (-\textsf{A}^0_\textrm{out}+\frac{1}{2} \textsf{A}^0_\textrm{res})\\&\quad +\,\bar{\partial }\textsf{A}^0_\textrm{res}\, (-\textsf{A}^{1,0}_\textrm{out}+\frac{1}{2} \textsf{A}^{1,0}_\textrm{res}+\frac{1}{2} \partial \textsf{A}^0_{I\,\textrm{res}}) +\partial \textsf{A}^0_\textrm{res}\, (\textsf{A}^{0,1}_\textrm{in}-\frac{1}{2} \textsf{A}^{0,1}_\textrm{res}+\frac{1}{2} \bar{\partial }\textsf{A}^0_{I\,\textrm{res}}) \Big ) . \end{aligned}$$

The sum of these three expressions is zero. $\square$

Similarly, one can check the quantum master equation for the “small” realization (33):

$$\begin{aligned} \left( \Omega ^+_\textrm{out}+ \Omega ^-_\textrm{in}-\hbar ^2 \Delta _\textrm{small} \right) \;Z_\textrm{small} =0, \end{aligned}$$

where

$$\begin{aligned} \Delta _\textrm{small}=2\int _\Sigma \frac{\delta }{\delta \textsf{A}^2_\textrm{res}}\frac{\delta }{\delta \sigma } +\frac{\delta }{\delta \textsf{A}^0_\textrm{res}}\frac{\delta }{\delta \textsf{A}^2_{I\,\textrm{res}}} \end{aligned}$$

is the BV Laplacian on $\mathcal {V}_\textrm{small}$.

Finally, the result of the full integration over residual fields (34) satisfies the BFV cocycle (gauge-invariance) condition

$$\begin{aligned} \left( \Omega ^+_\textrm{out}+ \Omega ^-_\textrm{in}\right) \; Z_*=0. \end{aligned}$$

## Chern–Simons Theory in “Parallel Ghost Polarization”

In three-dimensional Chern–Simons theory there is another way of picking a pair of polarizations on the opposite sides of a cylinder: we can use the $(\textsf{A}^0_\textrm{out},\textsf{A}^{0,1}_\textrm{out})$ representation on the out-boundary surface and the $(\textsf{A}^0_\textrm{in},\textsf{A}^{1,0}_\textrm{in})$ representation on the in-surface. Thus, the corresponding polarizations are transversal in ghost number 0 and parallel in ghost number $\ne 0$. See also the discussion of quantization of 1D systems with this class of polarizations in [14, Section 11].

### One-dimensional Chern–Simons theory with values in a cochain complex

As a warm-up, we consider again the one-dimensional theory, with a slightly different setup. Fix an odd integer *k*. Let

$$\begin{aligned} \mathfrak {g}= \bigoplus \mathfrak {g}^i \end{aligned}$$

be a graded vector space with a differential $d_\mathfrak {g}$ and a compatible graded symmetric pairing $(\cdot ,\cdot )$ of degree $-2k$.^29^ Now, we let $X=\mathfrak {g}[k]$ - this is a 0-shifted graded symplectic vector space. We call the induced grading on $C^\infty (X)$ the ghost number. It is convenient to express elements of $C^\infty (X)$ in terms of the shifted identity map $\psi \in \textrm{Hom}(X,\mathfrak {g})$ which has total degree (ghost number + degree) *k*.^30^ We denote the ghost number *l* component of a field $\varphi$ by $\varphi ^{[l]}$. In particular, the ghost number *l* component $\psi ^{[l]}$ of $\psi$ has degree $k - l$. For instance, the function

$$\begin{aligned} \Theta (\psi ) = \frac{1}{2}(\psi ,d_\mathfrak {g}\psi ) \end{aligned}$$

has ghost number $+1$. Its hamiltonian vector field *Q* has ghost number $+1$ and satisfies $Q^2 = 0$, thus $(X,\omega ,Q)$ is a BFV vector space.

We split the complexification of the ghost number 0 component of *X* as $X^{[0]}_\mathbb {C}= X^+ \oplus X^-$, with $X^\pm$ the degree 0 $\pm i$-eigenspaces of a complex structure *J* on $X^{[0]}$ compatible with the pairing. Thus, $X_\mathbb {C}$ admits the total decomposition

42 $$\begin{aligned} X_\mathbb {C}= X^{[>0]}_\mathbb {C}\oplus X^+ \oplus X^- \oplus X^{[<0]}_\mathbb {C} \end{aligned}$$

where $X^{[>0]},X^{[<0]}$ are the components of positive (resp. negative) ghost number.^31^ We also introduce the notations $d_\mathfrak {g}^+,d_\mathfrak {g}^-$ for the composition of the differential $X^{[1]} \rightarrow X^+ \oplus X^-$ with projections and similarly for the restriction of the differential ${ X^- \oplus X^+ \!\rightarrow \! X^{[-1]} }$ (so that $d_\mathfrak {g}^-=d_\mathfrak {g}\big |_{X^+}$, $d_\mathfrak {g}^+=d_\mathfrak {g}\big |_{X^-}$). We automatically have $(d_\mathfrak {g}^+)^2 = (d_\mathfrak {g}^-)^2 = 0$ and ${d_\mathfrak {g}^+d_\mathfrak {g}^- = - d_\mathfrak {g}^-d_\mathfrak {g}^+}$.^32^

#### Setup

We now consider the 1-dimensional AKSZ theory with target the symplectic graded vector space $(X,(\cdot ,\cdot ))$ and hamiltonian $\Theta (\psi )$. The space of fields is

$$\begin{aligned} \mathcal {F}= \Omega ^\bullet (I;X). \end{aligned}$$

It is parametrized by the superfield $\mathcal {A}$ valued in $\Omega ^\bullet (I;\mathfrak {g})$. We denote the 0- and 1-form components of $\mathcal {A}$ by $\psi$ and *A*, respectively. The total degrees of $\mathcal {A},\psi ,A$ are all odd. The action is

$$\begin{aligned} S[\psi + A]= \frac{1}{2}\int _I({\mathcal {A}},d_I\mathcal {A}) +\frac{1}{2} \int _I(\mathcal {A},d_\mathfrak {g}\mathcal {A}) = \frac{1}{2}\int _I (\psi ,d_I\psi ) +\int _I (A,d_\mathfrak {g}\psi ). \end{aligned}$$

The space of boundary fields is

$$\begin{aligned} \mathcal {F}^\partial = X_{\textrm{in}} \times X_{\textrm{out}} \ni (\psi _\textrm{in},\psi _\textrm{out}). \end{aligned}$$

The boundary 1-form is

$$\begin{aligned} \alpha _{\partial I} = \alpha ^{\partial }_\textrm{out}+ \alpha ^{\partial }_\textrm{in}= \frac{1}{2}(\psi _\textrm{out},\delta \psi _\textrm{out}) - \frac{1}{2}(\psi _\textrm{in},\delta \psi _\textrm{in}) = \frac{1}{2}(\psi ,\delta \psi )\bigg |_{t=0}^{t=1}. \end{aligned}$$

Splitting elements of $X_\mathbb {C}$ according to (42), $\psi = \psi ^{[<0]} + \psi ^+ + \psi ^- + \psi ^{[>0]}$, the boundary 1-form splits similarly:

$$\begin{aligned} \alpha ^{\partial }_\textrm{out}=\frac{1}{2}\Big [ (\psi _\textrm{out}^{[<0]},\delta \psi _\textrm{out}^{[>0]}) + (\psi _\textrm{out}^+,\delta \psi _\textrm{out}^-)+ (\psi _\textrm{out}^-,\delta \psi _\textrm{out}^+)+ (\psi _\textrm{out}^{[>0]},\delta \psi _\textrm{out}^{[<0]})\Big ] \end{aligned}$$

and similarly for $\alpha ^\partial _\textrm{in}$.

#### Parallel ghost polarization

Let us now consider the case where the polarizations are parallel in the ghost sector (of the target) and transversal in the physical sector:

$$\begin{aligned}\mathcal {P}&= \mathcal {P}_\textrm{in}\times \mathcal {P}_\textrm{out}, \\ \mathcal {P}_\textrm{in}&= \left\{ \frac{\delta }{\delta \psi ^{[<0]}_\textrm{in}},\frac{\delta }{\delta \psi _\textrm{in}^{+}}\right\} ,\\ \mathcal {P}_\textrm{out}&= \left\{ \frac{\delta }{\delta \psi ^{[<0]}_\textrm{out}},\frac{\delta }{\delta \psi _\textrm{out}^{-}}\right\} , \end{aligned}$$

so that we are using the $(\psi ^{[>0]}_\textrm{in},\psi ^{-}_\textrm{in})$ representation at $t=0$ and the $(\psi ^{[>0]}_\textrm{out},\psi ^{+}_\textrm{out})$ representation at $t=1$, i.e.:

$$\begin{aligned} \mathcal {B}= \mathcal {B}_\textrm{in}\times \mathcal {B}_\textrm{out}, \qquad \mathcal {B}_\textrm{in}= X^{[> 0]}_\mathbb {C}\oplus X^-, \qquad \mathcal {B}_\textrm{out}= X^{ [> 0]}_\mathbb {C}\oplus X^+. \end{aligned}$$

The polarized 1-form is $\alpha ^f_{\partial I} =\alpha ^{\partial ,\mathcal {P}_\textrm{out}} + \alpha ^{\partial ,\mathcal {P}_\textrm{in}}$ with

$$\begin{aligned} \alpha ^{\partial ,\mathcal {P}_\textrm{in}}&= -(\psi ^{[<0]}_\textrm{in},\delta \psi ^{[>0]}_\textrm{in}) - (\psi _\textrm{in}^{+},\delta \psi _\textrm{in}^-) = \alpha ^{\partial }_\textrm{in}- \delta f_\textrm{in}, \\ \alpha ^{\partial ,\mathcal {P}_\textrm{out}}&=(\psi ^{[<0]}_\textrm{out},\delta \psi ^{[>0]}_\textrm{out}) + (\psi _\textrm{out}^-,\delta \psi _\textrm{out}^{+}) = \alpha ^{\partial }_\textrm{out}+ \delta f_\textrm{out}, \end{aligned}$$

where^33^$f_\textrm{in}=\frac{1}{2} (\psi ^{[>0]}_\textrm{in},\psi ^{[<0]}_\textrm{in}) - \frac{1}{2}(\psi ^+_\textrm{in},\psi ^-_\textrm{in})$, $f_\textrm{out}= \frac{1}{2} (\psi ^{[>0]}_\textrm{out},\psi ^{[<0]}_\textrm{out}) + \frac{1}{2}(\psi _\textrm{out}^+,\psi _\textrm{out}^-)$, so that $\alpha ^f_{\partial I} = \alpha _{\partial I} + \delta f$ with

$$\begin{aligned} f (\psi _\textrm{out},\psi _\textrm{in})= & {} f_\textrm{out}(\psi _\textrm{out}) - f_\textrm{in}(\psi _\textrm{in}) \\= & {} \frac{1}{2} (\psi ^{[>0]}_\textrm{out},\psi ^{[<0]}_\textrm{out}) + \frac{1}{2}(\psi _\textrm{out}^+,\psi _\textrm{out}^{-}) -\frac{1}{2} (\psi ^{[>0]}_\textrm{in},\psi ^{[<0]}_\textrm{in}) + \frac{1}{2}(\psi _\textrm{in}^+,\psi _\textrm{in}^-) . \end{aligned}$$

The polarized action is

$$\begin{aligned} S^{f}[\mathcal {A}]= \frac{1}{2}\int _I({\mathcal {A}},d_I\mathcal {A}) +\frac{1}{2} \int _I(\mathcal {A},d_\mathfrak {g}\mathcal {A}) + f(\mathcal {A}). \end{aligned}$$

#### Gauge fixing

The kernel $\mathcal {Y}$ of the map $\mathcal {F} \rightarrow \mathcal {B}$ is

43 $$\begin{aligned} \mathcal {Y}= \Omega ^\bullet (I,\partial I; X^{[> 0]}_\mathbb {C}) \oplus \Omega ^\bullet (I,\{0\};X^-) \oplus \Omega ^\bullet (I,\{1\};X^+) \oplus \Omega ^\bullet (I;X^{[<0]}_\mathbb {C}). \end{aligned}$$

Choosing the minimal space of residual fields

$$\begin{aligned} \mathcal {V}= \langle dt \rangle \otimes X^{ [> 0]}_\mathbb {C}\oplus \langle 1 \rangle \otimes X^{ [<0]}_\mathbb {C}\ni {\mathcal {A}}_\textrm{res}= dt \cdot A_\textrm{res}+ 1\cdot \psi _{\textrm{res}}, \end{aligned}$$

we obtain

$$\begin{aligned} \mathcal {Y}= \mathcal {V}\times \mathcal {Y}' \ni (\mathcal {A}_\textrm{res},\mathcal {A}_\textrm{fl}) \end{aligned}$$

with

$$\begin{aligned} \mathcal {Y}' = \Omega ^\bullet (I,\partial I; X^{[> 0]}_\mathbb {C})_{\int = 0} \oplus \Omega ^\bullet (I,\{0\};X^-) \oplus \Omega ^\bullet (I,\{1\};X^+) \oplus \Omega ^\bullet (I;X^{[<0]}_\mathbb {C})_{\int \cdot \wedge dt=0}. \end{aligned}$$

Here the notation $\int \cdot = 0$ (resp. $\int \cdot dt = 0$) denotes acylic subcomplexes of forms with vanishing integral (resp. forms whose product with *dt* has vanishing integral). Choosing an extension

$$\begin{aligned} {\widetilde{\psi }} = \widetilde{\psi ^{[>0]}_\textrm{in}} + \widetilde{\psi _\textrm{in}^-} + \widetilde{\psi ^{[>0]}_\textrm{out}} + \widetilde{\psi _\textrm{out}^{+}} \end{aligned}$$

of boundary fields into the bulk, we obtain a splitting of $\mathcal {A}= \psi +A$ into

$$\begin{aligned} \psi + A = \widetilde{\psi } + \psi _\textrm{res}+ \psi _\textrm{fl}+ dt \cdot A_\textrm{res}+ A_\textrm{fl}. \end{aligned}$$

Inside $\mathcal {Y}'$, we have the gauge-fixing lagrangian $\mathcal {L}\subset \mathcal {Y}'$ given by forms of degree 0 in *I* —i.e., $\mathcal {L}$ is given by $A_\textrm{fl}= 0$—and we write for $\psi _\textrm{fl}\in \mathcal {L}$

$$\begin{aligned} \psi _\textrm{fl}= \psi _\textrm{fl}^{[>0]} + \psi ^-_\textrm{fl}+ \psi _\textrm{fl}^{+} + \psi _\textrm{fl}^{[<0]} . \end{aligned}$$

Recollecting, for a field $\psi +A \in \mathcal {B}\times \mathcal {V}\times \mathcal {L}$ we obtain

44 $$\begin{aligned} \psi&= \widetilde{\psi } + \psi _\textrm{res}+ \psi _\textrm{fl}\nonumber \\&= \widetilde{\psi ^{[>0]}_\textrm{in}} + \widetilde{\psi _\textrm{in}^-} + \widetilde{\psi ^{[>0]}_\textrm{out}} + \widetilde{\psi _\textrm{out}^{+}} + \psi _\textrm{res}+ \psi _\textrm{fl}^{[>0]} + \psi ^-_\textrm{fl}+ \psi _\textrm{fl}^{+} + \psi _\textrm{fl}^{[<0]}, \nonumber \\ A&= dt \cdot A_{\textrm{res}}. \end{aligned}$$

The gauge-fixed polarized action is then computed as follows:

##### Lemma 5.1

Restricted to the gauge-fixing lagrangian, the polarized action can be written as

45 $$\begin{aligned} S^f[\textsf{A}]= & {} S_{\textrm{source}}[\psi _\textrm{in},\psi _\textrm{out},\psi _\textrm{fl}] + S_0[\psi _\textrm{fl}] + S_{\textrm{int}}[\psi _\textrm{res},\psi _\textrm{fl},A_\textrm{res}]\nonumber \\{} & {} +S_{\textrm{back}}[\psi _\textrm{in},\psi _\textrm{out},\psi _\textrm{res},A_\textrm{res}] , \end{aligned}$$

where

$$\begin{aligned} S_{\textrm{source}}[\psi _\textrm{in},\psi _\textrm{out},\psi _\textrm{res},\psi _\textrm{fl}]&= (\psi ^+_\textrm{out},\psi _\textrm{fl}^-(1)) - (\psi ^-_\textrm{in},\psi _\textrm{fl}^+(0))\\&\quad +\, (\psi ^{[>0]}_\textrm{out}, \psi ^{[<0]}_\textrm{fl}(1)) - (\psi ^{[>0]}_\textrm{in},\psi _\textrm{fl}^{[<0]}(0)) ,\\ S_{0}[\psi _\textrm{fl}]&= \int _I(\psi _\textrm{fl}^+,d_I\psi _\textrm{fl}^-) + \int _I(\psi ^{[<0]}_\textrm{fl},d_I\psi _\textrm{fl}^{[>0]}) , \\ S_{\textrm{int}}[\psi _\textrm{fl},A_\textrm{res}]&= -\int _I\,dt(d_\mathfrak {g}^+ A^{[1]}_{\textrm{res}},\psi _\textrm{fl}^-) - \int _I\,dt(d_\mathfrak {g}^-A^{[1]}_\textrm{res},\psi ^+_\textrm{fl})\\&\quad +\, \int _Idt (A_{\textrm{res}}^{[>1]},d_\mathfrak {g}\psi _\textrm{fl}^{[<0]}) , \\ S_{\textrm{back}}[\psi _\textrm{in},\psi _\textrm{out},\psi _\textrm{res},A_\textrm{res}]&=(\psi ^{[>0]}_\textrm{out}-\psi ^{[>0]}_\textrm{in},\psi _{\textrm{res}}) + (A_{\textrm{res}}^{[>1]},d_\mathfrak {g}\psi _{\textrm{res}}) . \end{aligned}$$

Here we have introduced the notation $A^{[1]}_\textrm{res},A^{[>1]}_\textrm{res}$ for the components of $A_{\textrm{res}}$ valued in $X^{[1]}$ and in $X^{[>1]}$, respectively.^34^

##### Proof

The polarized action is

$$\begin{aligned} S^f[\psi +A] = \frac{1}{2}\int _I (\psi ,d_I\psi ) + \int _I(A,d_\mathfrak {g}\psi ) + f(\psi ) , \end{aligned}$$

where

$$\begin{aligned} f(\psi )&= f_\textrm{out}(\psi (1)) - f_\textrm{in}(\psi (0))\\&= \frac{1}{2}( \psi ^{[>0]}_\textrm{out},\psi ^{[<0]}_\textrm{fl}(1)) + \frac{1}{2}(\psi ^+_\textrm{out},\psi ^-_\textrm{fl}(1)) -\frac{1}{2}(\psi ^{[>0]}_\textrm{in},\psi ^{[<0]}_\textrm{fl}(0)) \\ {}&\quad + \frac{1}{2} (\psi ^+_\textrm{fl}(0),\psi ^-_\textrm{in}) . \end{aligned}$$

Splitting $\psi$ as in (44) and letting the support of $\widetilde{\psi }$ go towards $\partial I$ we obtain that

$$\begin{aligned} \frac{1}{2} \int _I (\psi ,d_I\psi )&= \frac{1}{2}\int _I (\widetilde{\psi } + \psi _\textrm{res}+ \psi _\textrm{fl},d_I(\widetilde{\psi } + \psi _\textrm{res}+ \psi _\textrm{fl})) \\&=\frac{1}{2}(\widetilde{\psi },\psi _\textrm{res})\bigg |^{t=1}_{t=0} + \frac{1}{2}(\widetilde{\psi },\psi _\textrm{fl})\bigg |^{t=1}_{t=0} + \frac{1}{2}\int _I(\psi _\textrm{fl},d_I\psi _\textrm{fl}) \\&= \frac{1}{2}(\psi ^{[>0]}_\textrm{out}-\psi ^{[>0]}_\textrm{in},\psi _{\textrm{res}}) + \frac{1}{2}(\psi ^{[>0]}_\textrm{out},\psi _\textrm{fl}^{[<0]}(1)) + \frac{1}{2}(\psi _\textrm{out}^{+},\psi _\textrm{fl}^-(1))\\&\quad -\,\frac{1}{2}(\psi ^{[>0]}_\textrm{in},\psi ^{[<0]}_\textrm{fl}(0)) - \frac{1}{2} (\psi _\textrm{in}^-,\psi _\textrm{fl}^+(0)) + \int _I(\psi _\textrm{fl}^+,d_I\psi _\textrm{fl}^-)\\ {}&\quad + \int _I(\psi _\textrm{fl}^{[<0]},d_I\psi _\textrm{fl}^{[>0]})\end{aligned}$$

and

$$\begin{aligned} \int _I (dt \cdot A_\textrm{res},d_\mathfrak {g}\psi )&=-\int _I\,dt(d_\mathfrak {g}^+A^{[1]}_{\textrm{res}},\psi _\textrm{fl}^-) - \int _I\,dt(d_\mathfrak {g}^-A^{[1]}_\textrm{res},\psi ^+_\textrm{fl}) \\&\quad +\, \int _Idt (A_{\textrm{res}}^{[>1]},d_\mathfrak {g}\psi _\textrm{fl}^{[<0]}) + (A_{\textrm{res}}^{[>1]},d_\mathfrak {g}\psi _{\textrm{res}}). \end{aligned}$$

Collecting the various terms, we obtain (45). $\square$

Notice that adding *f* has the effect of doubling the boundary source terms.

#### Effective action

The effective action is defined by

$$\begin{aligned} Z&= e^{\frac{i}{\hbar }S_\textrm{eff}[\psi _\textrm{in},\psi _\textrm{out},\psi _\textrm{res},A_\textrm{res}]} = \int \mathcal {D}\psi _\textrm{fl}\, e^{\frac{i}{\hbar }S^f[\psi _\textrm{in},\psi _\textrm{out},\psi _\textrm{res},\psi _\textrm{fl},A_\textrm{res}]} \\&=e^{\frac{i}{\hbar }S_{\textrm{back}}}\int \mathcal {D}\psi _\textrm{fl}\, e^{\frac{i}{\hbar }(S_{\textrm{source}}+S_{0}+S_{\textrm{int}})} \end{aligned}$$

where the integral is defined in terms of Feynman diagrams.

##### Proposition 5.2

The effective action is given by

$$\begin{aligned} S_\textrm{eff}&= S_\textrm{ph}+S_\textrm{gh}\quad \text{ with } \\ S_\textrm{ph}&= (\psi ^+_\textrm{out},\psi ^-_\textrm{in}) + (\psi ^-_\textrm{in},d_\mathfrak {g}^+A^{[1]}_\textrm{res}) + (\psi _\textrm{out}^+,d_\mathfrak {g}^-A^{[1]}_\textrm{res}) + \frac{1}{2} (d_\mathfrak {g}^- A^{[1]}_\textrm{res},d_\mathfrak {g}^+ A^{[1]}_\textrm{res}), \\ S_\textrm{gh}&= (\psi ^{[>0]}_\textrm{out}- \psi ^{[>0]}_\textrm{in},\psi _\textrm{res}) + (A^{[>1]}_\textrm{res},d_\mathfrak {g}\psi _\textrm{res}) . \end{aligned}$$

##### Proof

**Fig. 2:**
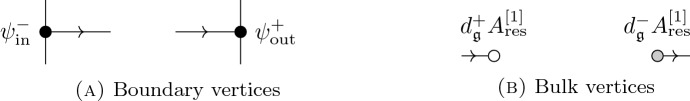
Vertices in 1D AKSZ theories with linear polarizations

In terms of Feynman diagrams, $S_{\textrm{source}}$ generates boundary vertices (Fig. 2a), $S_{\textrm{int}}$ generates bulk vertices (Fig. 2b).^35^

**Fig. 3:**
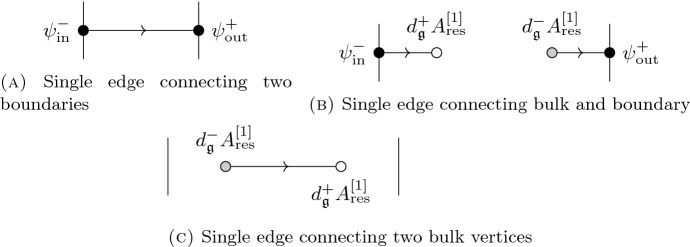
Connected Feynman diagrams in effective action

The term $S_0$ generates the propagators

$$\begin{aligned} \eta _{\textrm{ph}}(t,t')&=\frac{i}{\hbar } \langle \psi ^+_\textrm{fl}(t) \psi ^-_\textrm{fl}(t')\rangle = (\theta (t'-t))\cdot \omega ^{-1} \\ \eta _{\textrm{gh}}(t,t')&=\frac{i}{\hbar } \langle \psi _\textrm{fl}^{[>0]}(t) \psi _\textrm{fl}^{[<0]}(t')\rangle = (\theta (t-t')-t)\cdot \omega ^{-1} \end{aligned}$$

with $\theta (t)$ the Heaviside function and $\omega ^{-1}$ the inverse of the pairing on $\mathfrak {g}$. There are three types of connected Feynman diagrams contributing to $S_\textrm{eff}$ (see Fig. 3):

1. A single edge connecting the two boundary vertices (Fig. 3a). That diagram evaluates to $(\psi _\textrm{in}^-,\psi _\textrm{out}^+)$.
2. A single edge connecting a boundary vertex to a bulk vertex (Fig. 3b). Those diagrams yield $(\psi ^-_\textrm{in},d_\mathfrak {g}^+ A^{[1]}_\textrm{res}) + (\psi ^+_\textrm{out},d_\mathfrak {g}^- A^{[1]}_\textrm{res}).$
3. A single edge connecting the two bulk vertices (Fig. 3c). This diagram gives, using $\int _{I \times I}\eta _{\textrm{ph}}(t,t') = \frac{1}{2}$, the contribution $\frac{1}{2} (d_\mathfrak {g}^- A^{[1]}_\textrm{res},d_\mathfrak {g}^+ A^{[1]}_\textrm{res})$.

In total we obtain the effective action

46 $$\begin{aligned} S_{\textrm{eff}}[\psi _\textrm{in},\psi _\textrm{out},\psi _\textrm{res},A_\textrm{res}]= & {} (\psi ^{[>0]}_\textrm{out}-\psi ^{[>0]}_\textrm{in},\psi _\textrm{res}) - (\psi ^-_\textrm{in},\psi ^+_\textrm{out}) \nonumber \\{} & {} +\, (\psi ^-_\textrm{in},d_\mathfrak {g}^+ A^{[1]}_\textrm{res}) + (\psi _\textrm{out}^+,d_\mathfrak {g}^-A^{[1]}_\textrm{res})\nonumber \\{} & {} +\, \frac{1}{2} (d_\mathfrak {g}^- A^{[1]}_\textrm{res},d_\mathfrak {g}^+ A^{[1]}_\textrm{res}) + (A^{[>1]}_\textrm{res},d_\mathfrak {g}\psi _{\textrm{res}}). \end{aligned}$$

Separating the term depending only on ghost number 0 fields from the rest, we obtain the proof. $\square$

##### Proposition 5.3

The lagrangian generated by the $\textrm{gh}=0$ part of the action is the evolution relation in $X^{[0]}_\textrm{in}\times X^{[0]}_\textrm{out}$.

##### Proof

The Euler–Lagrange equations of the theory in ghost number 0 are

$$\begin{aligned} d_I\psi ^{[0]}&= -d_\mathfrak {g}A^{[1]}, \\ d_\mathfrak {g}\psi ^{[0]}&= 0. \end{aligned}$$

Projecting to boundary values $(\psi _\textrm{in},\psi _\textrm{out})$ we obtain the equations

47 $$\begin{aligned} d^-_\mathfrak {g}\psi ^+_\textrm{in}+ d_\mathfrak {g}^+\psi ^-_\textrm{in}&= d^+_\mathfrak {g}\psi ^-_\textrm{out}+ d_\mathfrak {g}^-\psi ^+_\textrm{out}= 0, \end{aligned}$$

48 $$\begin{aligned} \psi ^+_\textrm{out}- \psi ^+_\textrm{in}&= d^+_\mathfrak {g}a, \end{aligned}$$

49 $$\begin{aligned} \psi ^-_\textrm{out}- \psi ^-_\textrm{in}&= d^-_\mathfrak {g}b, \end{aligned}$$

for some $a,b \in X^{[1]}$, and the first equation forces $a=b$ (up to a $d_\mathfrak {g}^+$ and $d_\mathfrak {g}^-$-closed term). On the other hand, the lagrangian generated by $S_\textrm{ph}$ is given by

$$\begin{aligned} \psi ^+_\textrm{in}&= -\frac{\partial S_\textrm{ph}}{\partial \psi ^-_\textrm{in}} = \psi ^+_\textrm{out}- d_\mathfrak {g}^+A^{[1]}_\textrm{res}, \\ \psi ^-_\textrm{out}&= \frac{\partial S_\textrm{ph}}{\partial \psi ^+_\textrm{out}} = \psi ^-_\textrm{in}+ d_\mathfrak {g}^- A^{[1]}_\textrm{res}, \\ 0&= \frac{\partial S_\textrm{ph}}{\partial A^{[1]}_\textrm{res}} =d_\mathfrak {g}^+\psi _\textrm{in}^- + d_\mathfrak {g}^-\psi ^+_\textrm{out}+ d_\mathfrak {g}^+d_\mathfrak {g}^- A^{[1]}_\textrm{res}. \end{aligned}$$

The first two equations are equivalent to Eqs. (48), (49), while the last equation enforces the constraint (47). $\square$

#### Quantum master equation

The modified quantum master equation

$$\begin{aligned} (-\hbar ^2\Delta _\textrm{res}+ \Omega )e^{\frac{i}{\hbar }S_\textrm{eff}} \end{aligned}$$

is equivalent to

50 $$\begin{aligned} \left( \frac{1}{2}\left\{ S_\textrm{eff},S_\textrm{eff}\right\} _\textrm{res}- i\hbar \Delta _\textrm{res}S_\textrm{eff}+\Omega \right) Z =0 . \end{aligned}$$

Here we denote by $\{\cdot ,\cdot \}_\textrm{res}$ the BV ($+1$-shifted Poisson) bracket on $\mathcal {V}$.

##### Proposition 5.4

The effective action $S_\textrm{eff}$ given by (46) satisfies the mQME (50) with boundary BFV operator $\Omega$ given by the standard quantization of

$$\begin{aligned} \Theta (\psi ) = \frac{1}{2}(\psi ,d_\mathfrak {g}\psi ) = (\psi ^+,d_\mathfrak {g}^-\psi ^{[1]}) + (\psi ^-,d_\mathfrak {g}^+\psi ^{[1]}) + (\psi ^{[<0]},d_\mathfrak {g}\psi ^{[>1]}). \end{aligned}$$

##### Proof

Expanding degree-wise as a differential operator, we obtain $\Omega = \Omega ^{(1)} + \Omega ^{(0)} = \Omega ^{(1)}_\textrm{out}+ \Omega ^{(0)}_\textrm{out}+ \Omega ^{(1)}_\textrm{in}+ \Omega ^{(0)}_\textrm{in}$ with

$$\begin{aligned} \Omega ^{(0)}_\textrm{out}&= \left( d_\mathfrak {g}^-\psi ^{[1]}_\textrm{out},\psi ^+_\textrm{out}\right) , \\ \Omega ^{(0)}_\textrm{in}&= -\left( d_\mathfrak {g}^+\psi ^{[1]}_\textrm{in},\psi ^-_\textrm{in}\right) , \\ \frac{i}{\hbar }\Omega ^{(1)}_\textrm{out}&=\left( d_\mathfrak {g}^+\psi ^{[1]}_\textrm{out},\frac{\delta }{\delta \psi _\textrm{out}^+}\right) + \left( d_\mathfrak {g}\psi _\textrm{out}^{[>1]},\frac{\delta }{\delta \psi _\textrm{out}^{[>0]}}\right) ,\\ \frac{i}{\hbar }\Omega ^{(1)}_\textrm{in}&= \left( d_\mathfrak {g}^-\psi ^{[1]}_\textrm{in},\frac{\delta }{\delta \psi _\textrm{in}^-}\right) + \left( d_\mathfrak {g}\psi ^{[>1]}_\textrm{in},\frac{\delta }{\delta \psi ^{[>0]}_\textrm{in}}\right) . \end{aligned}$$

First of all, notice that $\Delta _\mathcal {V}S_\textrm{eff}= 0$ since in the only possibly nonvanishing term $(A^{[1]}_\textrm{res},d_\mathfrak {g}\psi _\textrm{res})$ fields are not paired with their antifields because of the degree shift by the differential. Computing the BV bracket we obtain

51 $$\begin{aligned}{} & {} \frac{1}{2} \{ S_\textrm{eff},S_\textrm{eff}\}_\textrm{res}\nonumber \\{} & {} \quad =- (\psi ^{[1]}_\textrm{out}-\psi ^{[1]}_\textrm{in},d_\mathfrak {g}^-\psi ^+_\textrm{out}+ d_\mathfrak {g}^+\psi ^-_\textrm{in}+ d_\mathfrak {g}^+d_\mathfrak {g}^- A^{[1]}_\textrm{res}) - (\psi ^{[>1]}_\textrm{out}- \psi ^{[>1]}_\textrm{in},d_\mathfrak {g}\psi _\textrm{res})\qquad \end{aligned}$$

(only terms of opposite ghost number survive in the pairing). On the other hand, since $\Omega ^{(0)}$ is a multiplication operator and $\Omega ^{(1)}$ contains only derivatives of first order, we have $\Omega Z = \Omega ^{(0)} Z + \frac{i}{\hbar }\Omega ^{(1)} (S_\textrm{eff}) Z$ and

$$\begin{aligned} \frac{i}{\hbar }\Omega ^{(1)}_\textrm{out}S_\textrm{eff}&= (\psi ^-_\textrm{in},d_\mathfrak {g}^+\psi ^{[1]}_\textrm{out}) + (d_\mathfrak {g}^+\psi ^{[1]}_\textrm{out},d_\mathfrak {g}^-A^{[1]}_\textrm{res})+ (d_\mathfrak {g}\psi ^{[>1]}_\textrm{out}, \psi _\textrm{res}), \\ \frac{i}{\hbar }\Omega ^{(1)}_\textrm{in}S_\textrm{eff}&= -(d_\mathfrak {g}^-\psi ^{[1]}_\textrm{in},\psi ^+_\textrm{out}) + (d_\mathfrak {g}^-\psi ^{[1]}_\textrm{in},d_\mathfrak {g}^+A^{[1]}_\textrm{res}) - (d_\mathfrak {g}\psi ^{[>1]}_\textrm{in}, \psi _\textrm{res}). \end{aligned}$$

A straightforward computation shows that $\Omega ^{(0)} +\frac{i}{\hbar }\Omega ^{(1)} S_\textrm{eff}$ coincides with (51), thus completing the proof. $\square$

### General polarizations

Next, we will consider the case where $X^{[0]}_\mathbb {C}$ is equipped with another polarization $\mathcal {P}_\textrm{nl}$ which is not necessarily linear (see [14, Section 12] for the corresponding toy model). Let the base $\mathcal {B}^{\mathcal {P}_\textrm{nl}}$ be locally parametrized by a coordinate $\psi ^Q$, and the fibers by a coordinate $\psi ^P$. Suppose $G(\psi ^-,\psi ^Q)$ is a generating function of the canonical transformation^36^$(\psi ^-,\psi ^+) \rightarrow (\psi ^Q,\psi ^P)$. Then we have that $\psi ^+ = F(\psi ^-,\psi ^Q) = \frac{\partial G}{\partial \psi ^-}$. We assume that *G* is analytic in $\psi ^-$ in a neighborhood *U* of $\{0\} \times \mathcal {B}^{\mathcal {P}_\textrm{nl}}$.

We now consider again the 1D AKSZ theory on the interval, where we choose the polarizations at the two endpoints to be parallel in the ghost sector. In the physical sector we choose the $\psi ^-$-representation on the in-boundary and the $\psi ^Q$-representation on the out-boundary:

$$\begin{aligned}\mathcal {P}&= \mathcal {P}_\textrm{in}\times \mathcal {P}_\textrm{out}\quad \text{ with } \\ \mathcal {P}_\textrm{in}&= \left\{ \frac{\delta }{\delta \psi ^{[<0]}_\textrm{in}},\frac{\delta }{\delta \psi _\textrm{in}^{+}}\right\} , \\ \mathcal {P}_\textrm{out}&= \left\{ \frac{\delta }{\delta \psi ^{[<0]}_\textrm{out}},\frac{\delta }{\delta \psi _\textrm{out}^{P}}\right\} . \end{aligned}$$

The base is

$$\begin{aligned} \mathcal {B}= \mathcal {B}_\textrm{in}\times \mathcal {B}_\textrm{out},\;\; \mathcal {B}_\textrm{in}= X^{[> 0]}_\mathbb {C}\oplus X^-,\;\; \mathcal {B}_\textrm{out}= X^{ [> 0]}_\mathbb {C}\times \mathcal {B}^{\mathcal {P}_{\textrm{nl}}}. \end{aligned}$$

The polarized 1-form is $\alpha ^f_{\partial I} =\alpha ^{\partial ,\mathcal {P}_\textrm{out}} + \alpha ^{\partial ,\mathcal {P}_\textrm{in}}$ with

$$\begin{aligned} \alpha ^{\partial ,\mathcal {P}_\textrm{in}}&= -(\psi ^{[<0]}_\textrm{in},\delta \psi ^{[>0]}_\textrm{in}) - (\psi _\textrm{in}^{+},\delta \psi _\textrm{in}^-) = \alpha ^{\partial }_\textrm{in}- \delta f_\textrm{in},\\ \alpha ^{\partial ,\mathcal {P}_\textrm{out}}&=(\psi ^{[<0]}_\textrm{out},\delta \psi ^{[>0]}_\textrm{out}) + (\psi _\textrm{out}^P,\delta \psi _\textrm{out}^{Q}) = \alpha ^{\partial }_\textrm{out}+ \delta f_\textrm{out}, \end{aligned}$$

where

$$\begin{aligned} f_\textrm{in}&=\frac{1}{2}( \psi ^{[>0]}_\textrm{in},\psi ^{[<0]}_\textrm{in}) - \frac{1}{2}(\psi ^ +_\textrm{in},\psi ^-_\textrm{in}), \\ f_\textrm{out}&= \frac{1}{2} (\psi ^{[>0]}_\textrm{out},\psi ^{[<0]}_\textrm{out}) - \frac{1}{2}(\psi _\textrm{out}^+,\psi _\textrm{out}^-) - G(\psi ^-_\textrm{out},\psi ^Q_\textrm{out}). \end{aligned}$$

Thus, $$\alpha ^f_{\partial I} = \alpha _{\partial I} + \delta f$$ with

$$\begin{aligned} f (\psi _\textrm{out},\psi _\textrm{in})= & {} f_\textrm{out}(\psi _\textrm{out}) - f_\textrm{in}(\psi _\textrm{in}) \\= & {} \frac{1}{2} (\psi ^{[>0]}_\textrm{out},\psi ^{[<0]}_\textrm{out}) - \frac{1}{2}(\psi _\textrm{out}^+,\psi _\textrm{out}^-) - G(\psi ^-_\textrm{out},\psi ^Q_\textrm{out})\nonumber \\{} & {} -\, \frac{1}{2} (\psi ^{[>0]}_\textrm{in},\psi ^{[<0]}_\textrm{in}) + \frac{1}{2}(\psi _\textrm{in}^+,\psi _\textrm{in}^-). \end{aligned}$$

#### Splitting the fields

The goal is to find again a symplectomorphism $\Phi :\mathcal {B}\times \mathcal {V}\times \mathcal {Y}' \rightarrow \mathcal {F}$.^37^ Here the trick is that we keep the space of fluctuations $\mathcal {Y}$ as above in Eq. (43). In ghost number zero, the map $\Phi$ is defined as follows. For boundary values $\psi ^-_\textrm{in}\in \mathcal {B}_\textrm{in}^{[0]}$ and $\psi ^Q_\textrm{out}\in \mathcal {B}_\textrm{out}^{[0]}$ and fluctuations $\psi ^-_\textrm{fl}, \psi ^+_\textrm{fl}\in \mathcal {Y}$ (recall that $\psi ^-_\textrm{fl}(0) = \psi ^+_\textrm{fl}(1)= 0$), we let

$$\begin{aligned} \psi ^-(t) = {\left\{ \begin{array}{ll} \psi ^-_\textrm{fl}(t) &{}\textrm{for} \;\; t > 0, \\ \psi ^-_\textrm{in}&{}\textrm{for} \;\; t = 0, \end{array}\right. } \end{aligned}$$

and

$$\begin{aligned} \psi ^+(t) = {\left\{ \begin{array}{ll} \psi ^+_\textrm{fl}(t) &{}\textrm{for} \;\; t < 1, \\ F(\psi ^-_\textrm{fl}(1),\psi ^Q_\textrm{out}) &{} \textrm{for}\;\; t = 1. \end{array}\right. } \end{aligned}$$

The map $\Phi$ is given by

52 $$\begin{aligned} \Phi (\psi ^{[>0]}_\textrm{in},\psi ^-_\textrm{in},\psi ^{[>0]}_\textrm{out},\psi ^{Q}_\textrm{out},\psi _\textrm{res},\psi _\textrm{fl},A)= & {} \psi ^-(\psi ^-_\textrm{in},\psi ^-_\textrm{fl}) + \psi ^+(\psi ^+_\textrm{fl},\psi ^-_\textrm{fl}, \psi ^Q_\textrm{out})\nonumber \\{} & {} +\,{\psi _\textrm{res}}+ \widetilde{\psi ^{[>0]}_\textrm{in}} + \widetilde{\psi ^{[>0]}_\textrm{out}} + \psi _\textrm{fl}^{[\ne 0]} + A.\qquad \end{aligned}$$

In nonzero ghost number, this coincides with the splitting considered in the previous section. In what follows, we will discuss only the physical sector, i.e., the part in ghost number 0. The analysis in the ghost sector proceeds exactly as in Sect. 5.1 and results in the ghost effective action $S_\textrm{gh}$ described in Proposition 5.2.

#### Effective action

Again, we can use the gauge-fixing lagrangian $\mathcal {L}\subset \mathcal {Y}'$ given by zero forms. Restricted to $\mathcal {B}\times \mathcal {V}\times \mathcal {L}$ and fields of ghost number 0, we have

$$\begin{aligned} S^f[\psi ^-_\textrm{in},\psi ^Q_\textrm{out},\psi ^-_\textrm{fl},\psi ^+_\textrm{fl},dt \cdot A_\textrm{res}]= & {} -(\psi ^-_\textrm{in},\psi ^+_\textrm{fl}(0)) - G(\psi ^-_\textrm{fl}(1),\psi ^Q_\textrm{out}) \\{} & {} +\, \int _I (\psi ^+_\textrm{fl},d_I \psi ^-_\textrm{fl}) - dt(d^+_\mathfrak {g}A^{[1]}_\textrm{res},\psi ^-_\textrm{fl})\\ {}{} & {} - dt(d^-_\mathfrak {g}A^{[1]}_\textrm{res},\psi ^+_\textrm{fl}) \end{aligned}$$

where the computation is very similar to the one in the proof of Lemma 5.1. The BV-BFV effective action is defined by

$$\begin{aligned} Z&= e^{\frac{i}{\hbar }S_\textrm{eff}[\psi _\textrm{in},\psi _\textrm{out},\psi _\textrm{res},A_\textrm{res}]} = \int \mathcal {D}\psi _\textrm{fl}\; e^{\frac{i}{\hbar }S^f[\psi _\textrm{in},\psi _\textrm{out},\psi _\textrm{res},\psi _\textrm{fl},A_\textrm{res}]} \\&=e^{\frac{i}{\hbar }S_{\textrm{back}}}\int \mathcal {D}\psi _\textrm{fl}\; e^{\frac{i}{\hbar }(S_{\textrm{source}}+S_{0}+S_{\textrm{int}})} \end{aligned}$$

where the integral is defined in terms of Feynman diagrams.

##### Proposition 5.5

The effective action (in ghost number 0) is

53 $$\begin{aligned} S_\textrm{eff}^\textrm{ph}[\psi _\textrm{in}^-,\psi _\textrm{out}^Q,A^{[1]}_\textrm{res}]= -G(\psi ^-_\textrm{in}+ d_\mathfrak {g}^- A^{[1]}_\textrm{res},\psi _\textrm{out}^Q) - (d_\mathfrak {g}^+A^{[1]}_\textrm{res},\psi _\textrm{in}^- + \frac{1}{2}d_\mathfrak {g}^-A^{[1]}_\textrm{res}) \end{aligned}$$

##### Proof

**Fig. 4:**
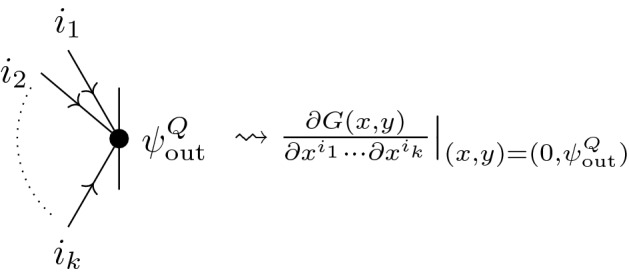
Additional vertex in 1D AKSZ theory with a general polarization on $X_\textrm{out}^{[0]}$

In terms of Feynman diagrams, the source term creates a vertex of arbitrary incoming valence on the out-boundary decorated by derivatives of *G* (see Fig. 4), and a univalent (outgoing) vertex on the in-boundary decorated by $\psi ^-_\textrm{in}$. The interaction term creates univalent in- and outgoing bulk vertices decorated by $d_\mathfrak {g}^\pm A^{[1]}_\textrm{res}$ as in the proof of Proposition 5.2.

**Fig. 5:**
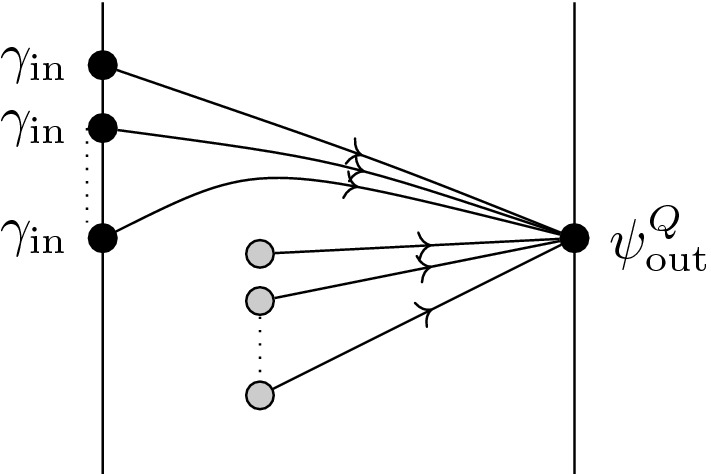
Additional Feynman diagrams in 1D AKSZ with general polarization on $X^{[0]}_\textrm{out}$

The connected Feynman diagrams contributing to the effective action are:

1. Diagrams involving the *G*-vertex on the out-boundary. The outgoing half-edges can connect either to the bulk vertex involving $\psi ^+_\textrm{fl}$ or the vertex on the in-boundary (see Fig. 5). Summing over all valences, we obtain the Taylor series in *x* of *G*(*x*, *y*) in the first argument at $(0,\psi _\textrm{out}^Q)$ evaluated on $\psi ^-_\textrm{in}+ d_\mathfrak {g}^-A^{[1]}_\textrm{res}$. Hence, by analyticity of *G* those vertices sum up to
  $$\begin{aligned} - G(\psi _\textrm{in}^- + d_\mathfrak {g}^-A^{[1]}_\textrm{res}, \psi _\textrm{out}^Q). \end{aligned}$$
2. Diagrams involving the univalent incoming bulk vertex. Here the outgoing half-edges connect to either the vertex on the in-boundary or an outgoing bulk vertex, giving
  $$\begin{aligned} -(d_\mathfrak {g}^+A^{[1]}_\textrm{res},\psi ^-_\textrm{in}+ \frac{1}{2} d_\mathfrak {g}^-A^{[1]}_\textrm{res}). \end{aligned}$$
  Those diagrams are the same as in the linear case (Fig. 3).

In total, we obtain the effective action (53). $\square$

##### Remark 5.6

In the main case of interest for this paper, the target $\mathfrak {g}= \Omega ^\bullet (M)$ is infinite-dimensional and the propagator contains a delta form as the “inverse” of the pairing $(\tau _1,\tau _2) = \int _M \tau _1 \wedge \tau _2$ (cf. Remark 4.1). However, our computations here are still valid. Indeed, even though Feynman diagrams contain products of delta functions, since all these diagrams are actually trees, no problematic terms like $\delta (0)$ arise when computing the integrals.

##### Proposition 5.7

The lagrangian generated by (53) is the evolution relation in $X_\textrm{in}^{[0]} \times X_\textrm{out}^{[0]}$.

##### Proof

We know that in the $\psi ^\pm$ variables, the evolution relation is given by $\psi ^-_\textrm{out}= \psi ^-_\textrm{in}+ d^-_\mathfrak {g}A^{[1]}_\textrm{res}$, $\psi ^+_\textrm{out}= \psi ^+_\textrm{in}+ d_\mathfrak {g}^+A^{[1]}_\textrm{res}$. The lagrangian generated by $S_\textrm{eff}^\textrm{ph}[\psi _\textrm{in}^-,\psi _\textrm{out}^Q,A^{[1]}_\textrm{res}]$ is

$$\begin{aligned} -\psi ^+_\textrm{in}&= \frac{\partial S_\textrm{eff}^\textrm{ph}}{\partial \psi ^-_\textrm{in}} = -F(\psi _\textrm{in}^-+d_\mathfrak {g}^- A^{[1]}_\textrm{res},\psi ^Q_\textrm{out}) + d_\mathfrak {g}^+A^{[1]}_\textrm{res}\\&= -F(\psi ^-_\textrm{out},\psi ^Q_\textrm{out}) + d_\mathfrak {g}^+A^{[1]}_\textrm{res}= -\psi ^+_\textrm{out}+ d_\mathfrak {g}^+A^{[1]}_\textrm{res}, \\ \psi ^P_\textrm{out}&=\frac{\partial S_\textrm{eff}^\textrm{ph}}{\partial \psi ^Q_\textrm{out}} = -\frac{\partial G(\psi ^-_\textrm{out},\psi _\textrm{out}^Q)}{\partial \psi ^Q_\textrm{out}}, \\ 0&= \frac{\partial S_\textrm{ph}}{\partial A^{[1]}_\textrm{res}} =d_\mathfrak {g}^+\psi _\textrm{in}^- + d_\mathfrak {g}^-\psi ^+_\textrm{out}+ d_\mathfrak {g}^+d_\mathfrak {g}^- A^{[1]}_\textrm{res}. \end{aligned}$$

This lagrangian coincides with the evolution relation. $\square$

#### Modified quantum master equation

Let us also comment on the mQME. Again, we can compute the BV bracket (we ignore higher ghosts for simplicity)

$$\begin{aligned} \frac{1}{2}\{S_\textrm{eff},S_\textrm{eff}\}_\textrm{res}= & {} \{S_\textrm{eff}^\textrm{gh},S_\textrm{eff}^\textrm{ph}\}\\= & {} -(\psi ^{[1]}_\textrm{out}-\psi ^{[1]}_\textrm{in},d_\mathfrak {g}^-F(\psi ^-_\textrm{in}+ d_\mathfrak {g}^-A^{[1]}_\textrm{res},\psi _\textrm{out}^Q) + d_\mathfrak {g}^+\psi ^-_\textrm{in}+ d_\mathfrak {g}^+d_\mathfrak {g}^- A^{[1]}_\textrm{res}). \end{aligned}$$

As before, we have $\Omega _\textrm{in}= -(d_\mathfrak {g}^+\psi _\textrm{in}^{[1]},\psi ^-_\textrm{in}) - i\hbar (d_\mathfrak {g}^-\psi _\textrm{in}^{[1]},\frac{\delta }{\delta \psi ^-_\textrm{in}})$ and

$$\begin{aligned} Z^{-1}\Omega _\textrm{in}Z&= -(d_\mathfrak {g}^+\psi _\textrm{in}^{[1]},\psi ^-_\textrm{in})Z - (d_\mathfrak {g}^-\psi ^{[1]}_\textrm{in},F(\psi ^-_\textrm{in}+ d_\mathfrak {g}^-A^{[1]}_\textrm{res},\psi _\textrm{out}^Q)) + (d_\mathfrak {g}^-\psi ^{[1]}_\textrm{in},d_\mathfrak {g}^+ A^{[1]}_\textrm{res}) \\&= - (\psi ^{[1]}_\textrm{in}, d_\mathfrak {g}^-F(\psi ^-_\textrm{in}+ d_\mathfrak {g}^-A^{[1]}_\textrm{res},\psi _\textrm{out}^Q) + d_\mathfrak {g}^+\psi ^-_\textrm{in}+ d_\mathfrak {g}^+d_\mathfrak {g}^- A^{[1]}_\textrm{res}). \end{aligned}$$

Thus, the mQME is equivalent to

54 $$\begin{aligned} Z^{-1}\Omega _\textrm{out}Z = (\psi ^{[1]}_\textrm{out}, d_\mathfrak {g}^-F(\psi ^-_\textrm{in}+ d_\mathfrak {g}^-A^{[1]}_\textrm{res},\psi _\textrm{out}^Q) + d_\mathfrak {g}^+\psi ^-_\textrm{in}+ d_\mathfrak {g}^+d_\mathfrak {g}^- A^{[1]}_\textrm{res}) = (\psi ^{[1]}_\textrm{out},d_\mathfrak {g}\psi ^{[0]}_\textrm{out}).\nonumber \\ \end{aligned}$$

The operator $\Omega _\textrm{out}$ acting on ghost number 0 fields should be obtained as a quantization of $\Theta (\psi )$ in the $\psi ^P_\textrm{out},\psi ^Q_\textrm{out}$ variables,

55 $$\begin{aligned} \Theta (\psi ^P_\textrm{out},\psi ^Q_\textrm{out}) = (d_\mathfrak {g}^+\psi ^{[1]}_\textrm{out},\psi ^-(\psi ^P_\textrm{out},\psi ^Q_\textrm{out})) + (d_\mathfrak {g}^-\psi ^{[1]}_\textrm{out},\psi ^+(\psi ^P_\textrm{out},\psi ^Q_\textrm{out})). \end{aligned}$$

The standard quantization $\Omega _\textrm{out}^{\textrm{std}}$ of (55)—i.e., replacing all $\psi ^P_\textrm{out}$ variables with $-i\hbar \delta /\delta \psi ^Q_\textrm{out}$ and moving all derivatives to the right—satisfies (54) to 0-th order in $\hbar$, but there are terms of higher order in $\hbar$ corresponding to higher derivatives in $\psi ^Q_\textrm{out}$ acting on *G*. To prove the mQME to all orders, one would have to find quantum corrections to $\Omega ^{\textrm{std}}_\textrm{out}$ such that these terms are cancelled and the deformed operator still squares to 0.

##### Remark 5.8

A particularly simple case occurs when $\psi (\psi ^P,\psi ^Q) = \psi ^P + \psi ^Q$. A rather trivial example of this case is $\psi ^Q = \psi ^+,\psi ^P = \psi ^-$. A nontrivial example will be considered in Sect. 6.3. In this case, we may define

$$\begin{aligned} \Omega _\textrm{out}= \left( d_\mathfrak {g}\psi ^{[1]}_\textrm{out},\psi ^Q_\textrm{out}- i\hbar \frac{\delta }{\delta \psi ^Q_\textrm{out}}\right) . \end{aligned}$$

Then, from $\partial G/\partial \psi ^Q = \psi ^P$ we immediately get $Z^{-1}\Omega _\textrm{out}Z = (d_\mathfrak {g}\psi ^{[1]}_\textrm{out},\psi ^Q_\textrm{out}+ \psi ^P_\textrm{out}) = (\psi ^{[1]}_\textrm{out},d_\mathfrak {g}\psi ^{[0]}_\textrm{out})$, i.e., Eq. (54), and hence the mQME, are satisfied. In general, we have the mQME whenever the constraints are linear both in the original and the new momenta, see [14, Section 12].

##### Remark 5.9

Let $Z_{\textrm{nl},\perp }$ be the partition function with transversal ghost polarization and a general polarization in ghost number 0, to be precise, we are choosing the $(\psi ^-,\psi ^{[<0]})$ on the $\textrm{in}$-boundary and the $(\psi ^Q,\psi ^{[>0]})$ on the $\textrm{out}$-boundary. In this case, there are no residual fields, and following a computation similar to the above, one finds^38^

$$\begin{aligned} Z_{\textrm{nl},\perp } = \exp \left( -\frac{i}{\hbar }G(\psi ^-_\textrm{in},\psi ^Q_\textrm{out}) + \frac{i}{\hbar }\psi ^{[<0]}_\textrm{in}\psi ^{[>0]}_\textrm{out}\right) . \end{aligned}$$

The mQME for this partition function is just $(\Omega _\textrm{in}+ \Omega _\textrm{out})Z = 0$, since there are no residual fields. We can observe that the only obstruction for the mQME to hold is the existence of a suitable $\Omega _\textrm{out}$ in a general polarization. Then, one can obtain the partition function $Z_{\textrm{nl},\parallel }$ -with parallel ghost polarization and $(\psi ^-,\psi ^Q)$-representation in ghost number 0, as given by (53) by composition of the partition function $Z_{\textrm{l},\parallel }$ with parallel ghost polarization and linear polarization in ghost number 0, given by (46) with the partition function $Z_{\textrm{nl},\perp }$: $Z_{\textrm{nl},\parallel } = Z_{\textrm{nl},\perp } \circ Z_{\textrm{l},\parallel }$. Since we know that $Z_{\textrm{l},\parallel }$ satisfies the mQME, $Z_{\textrm{nl},\parallel }$ will satisfy it if $Z_{\textrm{nl},\perp }$ does.

### 3D nonabelian Chern–Simons with parallel ghost polarization and antiho lomorphic-to-holomorphic polarization in ghost degree zero

Next, we return to the example of 3D Chern–Simons with parallel ghost polarization. In this context, it is convenient to use the traditional notation for the components of the superfield $\mathcal {A}$:

$$\begin{aligned} \mathcal {A} = c + A + A^* + c^* , \end{aligned}$$

where $\phi ^*$ denotes the BV antifield of the field $\phi$.

In this section we will use some special notations for field components (as compared to Sect. 4): ${a^{1,0}=\textsf{A}_\textrm{fl}^{1,0}}$, $a^{0,1}=\textsf{A}_\textrm{fl}^{0,1}$, $c=\textsf{A}^0$, $\textsf{A}^* = \textsf{A}^2$, $\sigma =\textsf{A}^0_{I\,\textrm{res}}$.

#### Abelian case

The action with polarization terms is:

$$\begin{aligned} S^f= \int _{ I\times \Sigma } \frac{1}{2} \mathcal {A}d \mathcal {A}+ \int _{\{1\}\times \Sigma } \frac{1}{2} \left( \textsf{A}^{1,0} \textsf{A}^{0,1}+c \textsf{A}^* \right) - \int _{\{0\}\times \Sigma } \frac{1}{2} \left( \textsf{A}^{0,1} \textsf{A}^{1,0}+c \textsf{A}^* \right) . \end{aligned}$$

The space of fields is:

$$\begin{aligned} \mathcal {F}=\Omega ^\bullet (I,\Omega ^{1,0}\oplus \Omega ^{0,1}\oplus \Omega ^0[1] \oplus \Omega ^2[-1]) \end{aligned}$$

—here $\Omega ^p$ in the coefficients stands for $\Omega ^p(\Sigma )$. It is fibered over

$$\begin{aligned} \mathcal {B}=(\Omega ^{0,1}\oplus \Omega ^0[1])\bigoplus (\Omega ^{1,0}\oplus \Omega ^0[-1])\;\; \ni ((\textsf{A}^{0,1}_\textrm{in},c_\textrm{in}),\; (\textsf{A}^{1,0}_\textrm{out},c_\textrm{out})) \end{aligned}$$

with fiber

$$\begin{aligned} \mathcal {Y}=\Omega ^\bullet (I,\{0\};\Omega ^{0,1})\oplus \Omega ^\bullet (I,\{1\};\Omega ^{1,0})\oplus \Omega ^\bullet (I,\{0,1\};\Omega ^0[1])\oplus \Omega ^\bullet (I;\Omega ^2[-1]) . \end{aligned}$$

The space of residual fields is given by the (relative) cohomology in *I*-direction:

$$\begin{aligned} \mathcal {V}=H^\bullet (I,\{0,1\};\Omega ^0[1])\oplus H^\bullet (I;\Omega ^2[-1])\quad \ni (dt\cdot \sigma , \textsf{A}^*_\textrm{res}) . \end{aligned}$$

The gauge-fixing lagrangian $\mathcal {L}$ in the fiber of $\mathcal {Y}\rightarrow \mathcal {V}$ is given by setting to zero the (relatively) exact 1-form components of fields along *I*.

Thus, on $\mathcal {L}$ we have

$$\begin{aligned} \textrm{gh}=0:\quad&A^{(1)}=\widetilde{\textsf{A}}^{1,0}_\textrm{out}+\widetilde{\textsf{A}}^{0,1}_\textrm{in}+a^{1,0}+a^{0,1}+dt\cdot \sigma , \\ \textrm{gh}=1:\quad&A^{(0)}=\widetilde{c}_\textrm{out}+\widetilde{c}_\textrm{in}+c_\textrm{fl},\\ \textrm{gh}=-1: \quad&A^{(2)}=\textsf{A}^*_\textrm{res}+\textsf{A}^*_\textrm{fl},\\ \textrm{gh}=-2:\quad&A^{(3)}=0 , \end{aligned}$$

with tilde denoting the discontinuous extension by zero from $t=1$ or $t=0$, respectively. Fluctuations are understood to satisfy

$$\begin{aligned} a^{1,0}|_{t=1}=0,\quad a^{0,1}|_{t=0}=0 , \quad c_\textrm{fl}|_{t=0}=c_\textrm{fl}|_{t=1}=0,\quad \int _0^1 dt\; \textsf{A}^*_\textrm{fl}=0 . \end{aligned}$$

The gauge-fixed polarized action is:

$$\begin{aligned} S^f|_\mathcal {L}= & {} \int _{ I\times \Sigma } a^{1,0} d_Ia^{0,1} + \int _{ I\times \Sigma } dt\, (a^{1,0}+a^{0,1}) d_\Sigma \sigma + \int _\Sigma \textsf{A}^{1,0}_\textrm{out}a^{0,1}\big |_{t=1} - \int _\Sigma \textsf{A}^{0,1}_\textrm{in}a^{1,0}\big |_{t=0}\\{} & {} +\,\int _{ I\times \Sigma } \textsf{A}^*_\textrm{fl}d_Ic_\textrm{fl}-\int _\Sigma (\textsf{A}^*_\textrm{res}+\textsf{A}^*_\textrm{fl}\big |_{t=1}) c_\textrm{out}+ \int _\Sigma (\textsf{A}^*_\textrm{res}+\textsf{A}^*_\textrm{fl}\big |_{t=0}) c_\textrm{in}. \end{aligned}$$

The propagators are given by:

56 $$\begin{aligned} \langle a^{0,1}(t,z) a^{1,0}(t',z') \rangle&=-i\hbar \, \theta (t-t')\, \delta ^{(2)}(z-z')\frac{i}{2}d\bar{z}\,dz' , \end{aligned}$$

57 $$\begin{aligned} \langle c_\textrm{fl}(t,z) \textsf{A}^*_\textrm{fl}(t',z') \rangle&=-i\hbar \, (\theta (t-t')-t)\, \delta ^{(2)}(z-z')\frac{i}{2}dz'\,d\bar{z}' . \end{aligned}$$

The corresponding effective action is:

58 $$\begin{aligned} S^\textrm{eff}=\int _\Sigma \textsf{A}^{1,0}_\textrm{out}\textsf{A}^{0,1}_\textrm{in}+ \textsf{A}^{1,0}_\textrm{out}\bar{\partial }\sigma + \textsf{A}^{0,1}_\textrm{in}\partial \sigma -\frac{1}{2} \partial \sigma \bar{\partial }\sigma - \textsf{A}^*_\textrm{res}(c_\textrm{out}-c_\textrm{in}). \end{aligned}$$

##### Remark 5.10

(*Hamilton–Jacobi property, mQME*) Notice that (58) coincides with (46) above upon specializing $\mathfrak {g}= \Omega ^\bullet (\Sigma )$, $X^+ = \Omega ^{1,0}(\Sigma )$, $X^-=\Omega ^{0,1}(\Sigma )$. Thus (58) satisfies the modified quantum master equation, and the $\textrm{gh}=0$ part of (58) generates the evolution relation of abelian Chern–Simons theory.

##### Remark 5.11

( Integrating out residual fields) As in Sect. 4.2.2, we can integrate out the residual fields $\sigma ,\textsf{A}_\textrm{res}^*$ by choosing a Riemannian metric compatible with the complex structure and decomposing fields as $\sigma = \sigma _c + \underline{\sigma }, \textsf{A}^*_\textrm{res}= \mu \cdot \textsf{A}^*_{\textrm{res},c}+\underline{\textsf{A}^*_\textrm{res}}$. As expected, the result differs from (34) only in the ghost sector:

$$\begin{aligned} Z_* [c_\textrm{out},c_\textrm{in},\textsf{A}^{1,0}_\textrm{out},\textsf{A}^{0,1}_\textrm{in}] = \delta (c_{\textrm{out},c} - c_{\textrm{in},c}) \big ({\det }'_{\Omega ^0(\Sigma )}\Delta _g\big )^{-\frac{1}{2}}\cdot e^{\frac{i}{\hbar } \mathbb {I}(\textsf{A}^{1,0}_\textrm{out},\textsf{A}^{0,1}_\textrm{in}) }. \end{aligned}$$

Here $\mathbb {I}$ is given by (35).

#### Nonabelian case

In the nonabelian Chern–Simons theory with coefficients in a semisimple Lie algebra $\mathcal {G}$ (corresponding to a compact group^39^*G*), the superfield is $\mathcal {A}\in \Omega ^\bullet ( I\times \Sigma , \mathcal {G}[1])$ and all the splittings are as before, just with components understood as $\mathcal {G}$-valued forms, paired in the quadratic part of the action via the Killing form $\langle ,\rangle$ on $\mathcal {G}$. The interaction term of the nonabelian theory, when restricted to the gauge-fixing lagrangian, yields

$$\begin{aligned} S_\textrm{int}=\frac{1}{6} \int \langle \mathcal {A},[\mathcal {A},\mathcal {A}] \rangle = -\int _\Sigma \int _I dt \langle a^{1,0},\textrm{ad}_\sigma a^{0,1} \rangle -\int _\Sigma \int _I dt \langle c_\textrm{fl},\textrm{ad}_\sigma (\textsf{A}^*_\textrm{res}+\textsf{A}^*_\textrm{fl}) \rangle . \end{aligned}$$

This adds two new bivalent vertices and a univalent vertex to the Feynman rules.

Let us introduce the following notations:

59 $$\begin{aligned} F_+(x)= & {} \frac{x}{1-e^{-x}}= \sum _{n\ge 0}(-1)^n\frac{B_{n}}{n!}x^n ,\quad F_-(x)=-\frac{x}{e^x-1}= -\sum _{n\ge 0} \frac{B_n}{n!} x^n, \end{aligned}$$

60 $$\begin{aligned} j(\sigma )= & {} \sum _{n=2}^\infty \frac{B_n}{n\cdot n!}\textrm{tr}_\mathcal {G}(\textrm{ad}_\sigma )^n = \textrm{tr}_\mathcal {G}\log \frac{\sinh \frac{\textrm{ad}_\sigma }{2}}{\frac{\textrm{ad}_\sigma }{2}}, \end{aligned}$$

with $B_n$ the Bernoulli numbers, $B_0=1,\; B_1=-\frac{1}{2},\; B_2=\frac{1}{6},\; B_3=0,\; B_4=-\frac{1}{30},\ldots$

In the following lemma we assume that $\sigma$ is in a sufficiently small neighborhood of zero in $\mathcal {G}$, see Remark 5.13 below for details.

##### Lemma 5.12

The partition function of the nonabelian Chern–Simons theory on a cylinder is $Z=e^{\frac{i}{\hbar }S^\textrm{eff}}$ with the following effective action:

61 $$\begin{aligned} S^\textrm{eff}= & {} \int _\Sigma \langle \textsf{A}^{1,0}_\textrm{out}, e^{-\textrm{ad}_\sigma }\circ \textsf{A}^{0,1}_\textrm{in}\rangle + \langle \textsf{A}^{1,0}_\textrm{out}, \frac{1-e^{-\textrm{ad}_\sigma }}{\textrm{ad}_\sigma }\circ \bar{\partial }\sigma \rangle + \langle \textsf{A}^{0,1}_\textrm{in}, \frac{e^{\textrm{ad}_\sigma }-1}{\textrm{ad}_\sigma } \circ \partial \sigma \rangle \nonumber \\{} & {} -\,\langle \partial \sigma ,\frac{e^{-\textrm{ad}_\sigma }+\textrm{ad}_\sigma -1}{(\textrm{ad}_\sigma )^2} \circ \bar{\partial }\sigma \rangle - \langle \textsf{A}^*_\textrm{res},F_+(\textrm{ad}_\sigma )\circ c_\textrm{out}+ F_-(\textrm{ad}_\sigma )\circ c_\textrm{in}\rangle \nonumber \\{} & {} -i\hbar \mathbb {W}(\sigma ). \end{aligned}$$

In (61), the 1-loop correction $\mathbb {W}$ stands for the contribution of “ghost wheels”—cycles of $n\ge 1$ ghost-antifield propagators (at the vertices, they interact with the residual field $\sigma$). These graphs are ill-defined in the chosen axial gauge; their formal evaluation yields the expression

62 $$\begin{aligned} \mathbb {W}(\sigma )= \sum _{n\ge 1} \frac{B_n}{n\cdot n!}\, \textrm{tr}_{C^\infty (\Sigma ,\mathcal {G})} (\textrm{ad}_\sigma )^n = \textrm{tr}_{C^\infty (\Sigma )} j(\sigma )\cdot \end{aligned}$$

This expression heuristically stands for the “sum over points *z* of $\Sigma$” of $j(\sigma (z))$.

We refer the reader to Cattaneo et al. [14, Section 11.3] for a one-dimensional toy model of this statement.

##### Proof

One has the following classes of Feynman diagrams contributing to the effective action:

Here the solid lines represent the “physical propagator” (56) and the dashed lines represent the “ghost propagator” (57).

These diagrams are calculated easiest by introducing the propagators dressed with $\sigma$-insertions:

$$\begin{aligned}&\langle a^{0,1}(t,z)\otimes a^{1,0}(t',z') \rangle _\textrm{dressed}\\&\quad =-i\hbar \, \theta (t-t')\, \delta ^{(2)}(z-z')\frac{i}{2}d\bar{z}\,dz' \;\sum _{k=0}^\infty \int _{t'<t_1<\cdots<t_k<t}dt_1\cdots dt_k (-\textrm{ad}_\sigma )^k\\&\quad = -i\hbar \, \theta (t-t')\,e^{-(t-t')\textrm{ad}_\sigma }\, \delta ^{(2)}(z-z')\frac{i}{2}d\bar{z}\,dz',\\&\quad \langle c_\textrm{fl}(t,z) \otimes \textsf{A}^*_\textrm{fl}(t',z') \rangle _\textrm{dressed} =-i\hbar \, \delta ^{(2)}(z-z')\frac{i}{2}dz'\,d\bar{z}'\cdot \\&\quad \cdot \sum _{k=0}^\infty \underbrace{\int _{t_1,\ldots ,t_k\in [0,1]} dt_1\cdots dt_k \; (\theta (t-t_1)-t)\,(\theta (t_1-t_2)-t_1)\cdots (\theta (t_k-t')-t_k)}_{ {\left\{ \begin{array}{ll}\frac{B_{k+1}(1-t')-B_{k+1}(t-t')}{(k+1)!},\;\; t>t' \\ (-1)^k\frac{B_{k+1}(t'-t)-B_{k+1}(t')}{(k+1)!},\;\; t<t' \end{array}\right. } }\; (-\textrm{ad}_\sigma )^k\\&\qquad = -i\hbar \, \delta ^{(2)}(z-z')\frac{i}{2}dz'\,d\bar{z}'\cdot \frac{e^{(t'-t+\theta (t-t'))\textrm{ad}_\sigma }-e^{t'\textrm{ad}_\sigma }}{e^{\textrm{ad}_\sigma }-1}. \end{aligned}$$

Here $B_k(t)$ are the Bernoulli polynomials.

**Fig. 6:**
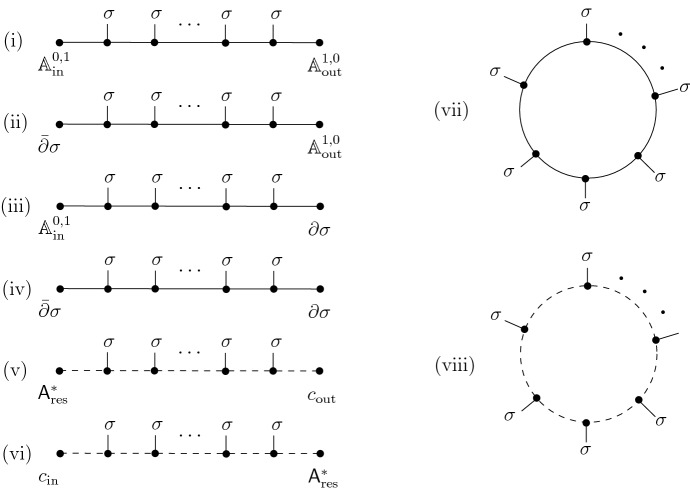
Feynman diagrams in nonabelian theory on a cylinder with “parallel ghost” polarization

Computing the tree Feynman diagrams (i)–(vi) in Fig. 6, we have the following.

(i). Here the contraction is the dressed propagator.
(ii).
(iii) Similarly to (5.3.2), .
(iv).
(v).
(vi) Similarly to (5.3.2), .

Thus, the Feynman diagrams (i)–(vi) in Fig. 6 yield the $O(\hbar ^0)$ part of the answer (61).

Next, consider the one-loop graphs in Fig. 6. The “physical wheels”—diagrams (vii)—vanish due to the form of the propagator (56): they are proportional to

$$\begin{aligned} \int _{t_1,\ldots ,t_k\in [0,1]} dt_1\cdots dt_k \, \theta (t_1-t_2)\theta (t_2-t_3)\cdots \theta (t_{k-1}-t_k) \theta (t_k-t_1) =0 . \end{aligned}$$

Finally, consider the “ghost wheels”—diagrams (viii). The propagator (57) is the integral kernel of an operator $K^\textrm{gh}=\textrm{id}\otimes K^\textrm{gh}_I$ acting on $C^\infty (\Sigma )\otimes \Omega ^\bullet (I)$ with $K^\textrm{gh}_I: f(t)+dt\, g(t) \mapsto \int _0^1dt'\,(\theta (t-t')-t)\,g(t')$. As a regularization, let us replace $C^\infty (\Sigma )$ with $C^\infty (X)$, with *X* a finite set of points—the set of vertices of some triangulation of the surface $\Sigma$. In particular, $C^\infty (X)$ is a finite-dimensional vector space. Then, the regularized value of the ghost wheel diagram (viii) with *k* $\sigma$-insertions is the supertrace:

$$\begin{aligned} -i\hbar \; \textrm{str}_{C^\infty (X)\otimes \Omega ^\bullet (I,\mathcal {G})} (- K^\textrm{gh}\, dt\, \textrm{ad}_\sigma )^k = -i\hbar \,\textrm{tr}_{C^\infty (X)}\, \textrm{str}_{\Omega ^\bullet (I,\mathcal {G})} (- K^\textrm{gh}_I\, dt\, \textrm{ad}_\sigma )^k. \end{aligned}$$

For the supertrace over the interval, we have (see, e.g., [32]):

$$\begin{aligned}{} & {} \textrm{str}_{\Omega ^\bullet (I,\mathcal {G})} (- K^\textrm{gh}_I\,dt\, \textrm{ad}_\sigma )^k\\{} & {} \quad = \textrm{tr}_\mathcal {G}\int _{t_1,\ldots ,t_k\in [0,1]} (\theta (t_1-t_2)-t_1)dt_2 \textrm{ad}_\sigma \cdots (\theta (t_{k-1}-t_k)-t_{k-1}) dt_k \textrm{ad}_\sigma \\{} & {} \qquad \times (\theta (t_k-t_1)-t_1) dt_1 \textrm{ad}_\sigma \\{} & {} \quad = \frac{B_k}{ k!}\textrm{tr}_\mathcal {G}(\textrm{ad}_\sigma )^k . \end{aligned}$$

Summing over the values of $k\ge 1$ and taking into account the symmetric factor 1/*k* (due to the automorphisms of the wheel graph), we obtain

$$\begin{aligned} \sum _{k\ge 1}\frac{1}{k}\textrm{str}_{\Omega ^\bullet (I,\mathcal {G})} (- K^\textrm{gh}_I\, dt\, \textrm{ad}_\sigma )^k = j(\sigma ), \end{aligned}$$

with *j* as in (60). Thus, finally, the total contribution of graphs (viii) to the effective action is

$$\begin{aligned} -i\hbar \,\mathbb {W}= -i\hbar \, \textrm{tr}_{C^\infty (X)}j(\sigma )=-i\hbar \sum _{z\in X} j(\sigma (z)). \end{aligned}$$

Trying to pass to a limit of dense triangulation *X* obviously leads to an ill-defined result here.

Put another way, the regularized computation of a ghost wheel diagram is:where the contractions are the nondressed propagators (57) with the delta form in *z* replaced with Kronecker symbol $\delta _{z z'}$. In this regularized setup we understand the fields $c_\textrm{fl},\textsf{A}^*_\textrm{fl},\sigma$ as supported at the vertices of *X*; fields $c_\textrm{fl},\textsf{A}^*_\textrm{fl}$ also depend on $t\in [0,1]$. $\square$

##### Remark 5.13

In Lemma 5.12 we assumed that the residual field $\sigma$ takes values in a sufficiently small neighborhood of zero in $\mathcal {G}$, so that the sums of Feynman diagrams in Fig. 6 converge.^40^ In fact, they converge iff $\sigma$ is valued in $B_0\subset \mathcal {G}$ where $B_0$ is the connected component of the origin in

$$\begin{aligned} \left\{ \sigma \in \mathcal {G}\,\big |\, {\det }_\mathcal {G}\frac{\sinh \frac{\textrm{ad}_\sigma }{2}}{\frac{\textrm{ad}_\sigma }{2}}\ne 0\right\} \qquad \subset \quad \mathcal {G}. \end{aligned}$$

In other words, $B_0$ is the subset of $\mathcal {G}$ where all eigenvalues of $\textrm{ad}_\sigma$ lie in the open interval $(-2\pi i, 2\pi i)\subset i\mathbb {R}$. Thus, we are assuming that $\sigma$ takes values in $B_0\subset \mathcal {G}$ (cf. the discussion of the Gribov region in the context of 2D Yang–Mills in [28, Section 2.4.1]). Furthermore, note that the exponential map $\exp :\mathcal {G}\rightarrow G$ is a diffeomorphism from $B_0$ onto its image $\exp (B_0)$. Moreover, $\exp (B_0)$ is an open dense subset of *G*.

#### Group-valued parametrization of the residual field

Let us parametrize the residual field $\sigma$ by a group-valued map $g=e^{-\sigma }:\Sigma \rightarrow G$.

##### Lemma 5.14

The effective action (61) can be rewritten as

63 $$\begin{aligned} S^\textrm{eff}= & {} \int _\Sigma \Big ( \langle \textsf{A}^{1,0}_\textrm{out}, g\, \textsf{A}^{0,1}_\textrm{in}g^{-1} \rangle - \langle \textsf{A}^{1,0}_\textrm{out},\bar{\partial }g\cdot g^{-1} \rangle - \langle \textsf{A}^{0,1}_\textrm{in}, g^{-1}\, \partial g \rangle \nonumber \\{} & {} +\, \langle \textsf{A}^*_\textrm{res}, F_-(\textrm{ad}_{\log g})\circ c_\textrm{out}+F_+(\textrm{ad}_{\log g})\circ c_\textrm{in}\rangle \Big ) +\textrm{WZW}(g) - i\hbar \mathbb {W}. \end{aligned}$$

Here

64 $$\begin{aligned} \textrm{WZW}(g)=- \frac{1}{2} \int _\Sigma \langle \partial g\cdot g^{-1}, \bar{\partial }g\cdot g^{-1} \rangle -\frac{1}{12} \int _{I\times \Sigma }\langle d\widetilde{g}\cdot \widetilde{g}^{-1},[d\widetilde{g}\cdot \widetilde{g}^{-1},d\widetilde{g}\cdot \widetilde{g}^{-1}] \rangle .\nonumber \\ \end{aligned}$$

is the Wess–Zumino–Witten action, where $\widetilde{g}=e^{(t-1)\sigma }$ is the extension of *g* to a mapping $I\times \Sigma \rightarrow G$, interpolating between $\widetilde{g}=g$ at $t=0$ and $\widetilde{g}=1$ at $t=1$.^41^

##### Remark 5.15

Under the convergence assumption that $\sigma$ is valued in $B_0$ (see Remark 5.13), or equivalently that *g* is valued in $\exp (B_0)$—a contractible open dense subset of *G*, $\textrm{WZW}(g)$ is a single-valued function of *g*, and hence $S^\textrm{eff}$ is also a single-valued expression. If *g* is allowed to roam the entire group *G*, $\textrm{WZW}(g)$ (and thus $S^\textrm{eff}$) becomes multi-valued, defined only $\bmod \, 4\pi ^2\mathbb {Z}$. In the latter case, for $e^{\frac{i}{\hbar }S^\textrm{eff}}$ to be a single-valued expression, one needs $\hbar =\frac{2\pi }{k}$ with $k\in \mathbb {Z}$ an integer level. The fact that quantization of $\hbar$ is necessary in one case but not in the other can be traced to the fact that the Cartan 3-form [whose pullback by $\widetilde{g}$ is the integrand in the second term in the r.h.s. of (64)] represents a nontrivial cohomology class on *G* but is exact when restricted to $\exp (B_0)$.

##### Proof of Lemma 5.14

First terms in (61) and (63) obviously match. We have

$$\begin{aligned} \begin{aligned} g^{-1}\partial g&= e^{\sigma }\int _0^1 d\tau \, e^{-\tau \sigma }(-\partial \sigma ) e^{-(1-\tau )\sigma } = \int _0^1 d\tau \, e^{\tau \textrm{ad}_\sigma }(-\partial \sigma )\\&=\frac{e^{\textrm{ad}_\sigma }-1}{\textrm{ad}_\sigma }(-\partial \sigma ) ,\\ \bar{\partial }g\cdot g^{-1}&= \int _0^1 d\tau \, e^{-\tau \sigma }(-\bar{\partial }\sigma )e^{-(1-\tau )\sigma }e^\sigma = \int _0^1 d\tau \, e^{-\tau \textrm{ad}_\sigma }(-\bar{\partial }\sigma ) \\ {}&= \frac{1-e^{-\textrm{ad}_\sigma }}{\textrm{ad}_\sigma }(-\bar{\partial }\sigma ). \end{aligned} \end{aligned}$$

Thus, second and third terms in (61) and (63) also match. Next, evaluating the Wess–Zumino term on our preferred extension $\widetilde{g}=e^{(t-1)\sigma }$, we have

65 $$\begin{aligned}{} & {} -\frac{1}{12}\int _{ I\times \Sigma } \langle d\widetilde{g}\cdot \widetilde{g}^{-1},[d\widetilde{g}\cdot \widetilde{g}^{-1},d\widetilde{g}\cdot \widetilde{g}^{-1}] \rangle \nonumber \\{} & {} \quad = -\frac{1}{4} \int _\Sigma \int _0^1 d t \Big \langle \sigma ,\int _0^{1-t}d\tau \int _0^{1-t}d\tau ' \Big [ e^{-\tau \sigma }(-d\sigma ) e^{-(1-t-\tau )\sigma }e^{(1-t)\sigma },\nonumber \\{} & {} \qquad \times e^{-\tau '\sigma }(-d\sigma ) e^{-(1-t-\tau ')\sigma }e^{(1-t)\sigma }\Big ] \Big \rangle \nonumber \\{} & {} \quad =\frac{1}{4} \int _\Sigma \int _0^1 dt \int _0^{1-t}d\tau \int _0^{1-t} d\tau ' \Big \langle d\sigma , \Big [\sigma , e^{(\tau '-\tau )\textrm{ad}_{\sigma }} d\sigma \Big ] \Big \rangle \nonumber \\{} & {} \quad = \frac{1}{2} \int _\Sigma \Big \langle d\sigma , \Big [\sigma , \Big (\frac{\sinh \textrm{ad}_\sigma -\textrm{ad}_\sigma }{(\textrm{ad}_\sigma )^3}\Big ) \, d\sigma \Big ] \Big \rangle \nonumber \\{} & {} \quad =\frac{1}{2} \int _\Sigma \Big \langle d\sigma , \Big (\frac{\sinh \textrm{ad}_\sigma -\textrm{ad}_\sigma }{(\textrm{ad}_\sigma )^2}\Big ) \, d\sigma \Big \rangle . \end{aligned}$$

The WZW kinetic term is:

66 $$\begin{aligned}{} & {} - \frac{1}{2} \int _\Sigma \langle \partial g\cdot g^{-1}, \bar{\partial }g\cdot g^{-1} \rangle \nonumber \\{} & {} \quad = -\frac{1}{2} \int _\Sigma \int _0^1 d\tau \int _0^1 d\tau ' \Big \langle e^{-\tau \sigma } (-\partial \sigma ) e^{-(1-\tau )\sigma }e^\sigma , e^{-\tau '\sigma } (-\bar{\partial }\sigma ) e^{-(1-\tau ')\sigma }e^\sigma \Big \rangle \nonumber \\{} & {} \quad = -\frac{1}{2}\int _\Sigma \int _0^1 d\tau \int _0^1 d\tau ' \Big \langle \partial \sigma , e^{(\tau -\tau ')\textrm{ad}_\sigma } \bar{\partial }\sigma \Big \rangle = - \int _\Sigma \Big \langle \partial \sigma , \frac{\cosh \textrm{ad}_\sigma -1}{(\textrm{ad}_\sigma )^2} \bar{\partial }\sigma \Big \rangle .\qquad \end{aligned}$$

Putting the kinetic term (66) and the Wess–Zumino term (65) together, we obtain

$$\begin{aligned} \textrm{WZW}(g)= & {} \int _\Sigma -\Big \langle \partial \sigma , \frac{\cosh \textrm{ad}_\sigma -1}{(\textrm{ad}_\sigma )^2} \bar{\partial }\sigma \Big \rangle + \Big \langle \partial \sigma , \frac{\sinh \textrm{ad}_\sigma -\textrm{ad}_\sigma }{(\textrm{ad}_\sigma )^2} \, \bar{\partial }\sigma \Big \rangle \\= & {} -\int _\Sigma \Big \langle \partial \sigma , \frac{ e^{-\textrm{ad}_\sigma } +\textrm{ad}_\sigma -1}{(\textrm{ad}_\sigma )^2}\, \bar{\partial }\sigma \Big \rangle . \end{aligned}$$

Thus, finally, $\textrm{WZW}$ term in (63) coincides with the fourth term in (61).

Ghost terms and the 1-loop contributions in (61) and (63) are identified directly.

#### A comment on ghost wheels

To understand the role of the term $\mathbb {W}$ (ghost wheels) in (63), recall that, for $\mu _G$ the Haar measure on the group *G* and $\mu _\mathcal {G}$ the Lebesgue measure on the Lie algebra, one has $\exp ^*\mu _G=e^j\cdot \mu _\mathcal {G}$, with *j* the function on $\mathcal {G}$ defined by the formula (60). Therefore, the half-density on the space of residual fields associated to the effective action (63) is, heuristically, the following^42^:

67 $$\begin{aligned}{} & {} e^{\frac{i}{\hbar }S^\textrm{eff}}\mathcal {D}^{\frac{1}{2}} \sigma \; \mathcal {D}^{\frac{1}{2}} \textsf{A}^*_\textrm{res}\sim e^{\frac{i}{\hbar }S^\textrm{eff}}\mathcal {D}\sigma \nonumber \\{} & {} \quad = e^{\frac{i}{\hbar }S^{\textrm{eff}\,(0)}}\;\; ``\prod _{z\in \Sigma } \underbrace{e^{j(\sigma (z))}\mu _\mathcal {G}(\sigma (z))}_{\mu _G(g(z))} "= e^{\frac{i}{\hbar }S^{\textrm{eff}\,(0)}}\; \mathcal {D}g . \end{aligned}$$

Here $S^{\textrm{eff}\,(0)}$ stands for (63) without the $\mathbb {W}$ term^43^—the latter was used in transforming the functional measure from the pointwise product of Lebesgue measures for $\sigma$ to the product of Haar measures for *g*. The equivalence (67) of a half-densities is an extension of a rigorous result presented in [14] for a finite-dimensional system.

The odd symplectic form on residual fields is

68 $$\begin{aligned} \omega _\textrm{res}= & {} \int _\Sigma \langle \delta \textsf{A}^*_\textrm{res}, \delta \sigma \rangle = \delta \int _\Sigma \langle \textsf{A}^*_\textrm{res}, \delta \sigma \rangle \nonumber \\= & {} \delta \int _\Sigma \langle \textsf{A}^*_\textrm{res}, -\frac{\textrm{ad}_\sigma }{1-e^{-\textrm{ad}_\sigma }}\circ (\delta g\cdot g^{-1}) \rangle = \delta \int _\Sigma \langle -\frac{\textrm{ad}_\sigma }{e^{\textrm{ad}_\sigma }-1}\circ \textsf{A}^*_\textrm{res}, \delta g\cdot g^{-1} \rangle \nonumber \\= & {} \int _\Sigma \langle \delta g^*, \delta g \rangle , \end{aligned}$$

where we introduced the notation

69 $$\begin{aligned} g^*=-g^{-1}\cdot \big (F_+(\textrm{ad}_{\log g})\circ \textsf{A}^*_\textrm{res}\big ) = \big (F_-(\textrm{ad}_{\log g})\circ \textsf{A}^*_\textrm{res}\big )\cdot g^{-1} \end{aligned}$$

—a reparametrization of the residual field $\textsf{A}^*_\textrm{res}$ such that $(g,g^*)$ form Darboux coordinates on $\mathcal {V}$.

Rewritten in terms of the parametrization $(g,g^*)$ for residual fields, the half-density (67) becomes

70 $$\begin{aligned} e^{\frac{i}{\hbar }S^\textrm{eff}} \mathcal {D}^{\frac{1}{2}} \sigma \; \mathcal {D}^{\frac{1}{2}} \textsf{A}^*_\textrm{res}= e^{\frac{i}{\hbar }S^{\textrm{eff}\,(0)}} \mathcal {D}^{\frac{1}{2}} g\; \mathcal {D}^{\frac{1}{2}} g^* . \end{aligned}$$

I.e., in the $(g,g^*)$-parametrization, the ghost loops go away and the effective action has no quantum corrections.

##### Remark 5.16

In the context of BV formalism, it is natural to think of $S^\textrm{eff}$ as a “log-half-density” (see, e.g., [32, section 2.6]) on the space of residual fields, rather than a function, i.e., behaving under a change of Darboux coordinates as

$$\begin{aligned} S^\textrm{eff}_{[x,\xi ]}(x,\xi )=S^\textrm{eff}_{[x',\xi ']}(x',\xi ')-i\hbar \log \textrm{sdet} \frac{\partial (x,\xi )}{\partial (x',\xi ')} , \end{aligned}$$

so that one has $e^{\frac{i}{\hbar }S^\textrm{eff}_{[x,\xi ]}(x,\xi )}d^{\frac{1}{2}}x D^{\frac{1}{2}} \xi =e^{\frac{i}{\hbar }S^\textrm{eff}_{[x',\xi ']}(x',\xi ')}d^{\frac{1}{2}}x' D^{\frac{1}{2}} \xi '$. Here the superdeterminant (Berezinian) $\textrm{sdet}\cdots$ is the Jacobian of the transformation. With that in mind, the effective action (61) is $S^\textrm{eff}_{[\sigma ,\textsf{A}^*_\textrm{res}]}$—relative to the coordinate system $(\sigma ,\textsf{A}^*_\textrm{res})$ on $\mathcal {V}$. On the other hand, $S^{\textrm{eff}\,(0)}$ given by (63) without the $-i\hbar \mathbb {W}$ term is $S^\textrm{eff}_{[g,g^*]}$—relative to the coordinate system $(g,g^*)$.

#### Effective action vs. Hamilton–Jacobi

Denote

71 $$\begin{aligned}{} & {} \mathbb {I}(\textsf{A}^{0,1}_\textrm{in}, \textsf{A}^{1,0}_\textrm{out};g)\nonumber \\{} & {} \quad =\int _\Sigma \Big ( \langle \textsf{A}^{1,0}_\textrm{out}, g \textsf{A}^{0,1}_\textrm{in}g^{-1} \rangle - \langle \textsf{A}^{1,0}_\textrm{out},\bar{\partial }g\cdot g^{-1} \rangle - \langle \textsf{A}^{0,1}_\textrm{in}, g^{-1} \partial g \rangle \Big ) +\textrm{WZW}(g)\qquad \end{aligned}$$

—the effective action (63) restricted to fields of ghost number zero and without the $O(\hbar )$ term.

Function (71) produces, as a generalized generating function (see [14, Appendix A] and Sect. 2), with *g* an auxiliary parameter, the following lagrangian $L\subset \overline{\mathcal {F}^\partial _\textrm{in}}\times \mathcal {F}^\partial _\textrm{out}$ in the phase space for the boundary of the cylinder:

72 $$\begin{aligned}{} & {} L=\Big \{\textsf{A}|_{t=1}=\textsf{A}_\textrm{out}^{1,0}+\underbrace{g \textsf{A}^{0,1}_\textrm{in}g^{-1} -\bar{\partial }g\cdot g^{-1}}_{\textsf{A}^{0,1}\big |_{t=1}=\frac{\delta \mathbb {I}}{\delta \textsf{A}^{1,0}_\textrm{out}}}\;,\nonumber \\{} & {} \textsf{A}|_{t=0}=\textsf{A}_\textrm{in}^{0,1}+\underbrace{g^{-1} \textsf{A}^{1,0}_\textrm{out}g + g^{-1}\cdot \partial g}_{\textsf{A}^{1,0}\big |_{t=0}=-\frac{\delta \mathbb {I}}{\delta \textsf{A}^{0,1}_\textrm{in}}} \;\; \Big |\;\; Y=0 \Big \} , \end{aligned}$$

where we denoted^44^

73 $$\begin{aligned} Y= & {} -\frac{\delta \mathbb {I}}{\delta g\cdot g^{-1}}\nonumber \\= & {} [\textsf{A}_\textrm{out}^{1,0},g \textsf{A}^{0,1}_\textrm{in}g^{-1} ]+(\bar{\partial }-\textrm{ad}_{\bar{\partial }g\cdot g^{-1}}) \textsf{A}_\textrm{out}^{1,0} +\partial (g \textsf{A}^{0,1}_\textrm{in}g^{-1}) -\partial (\bar{\partial }g\cdot g^{-1}) . \end{aligned}$$

##### Lemma 5.17

The lagrangian (72) generated by the functional (71)—the tree part of the effective action, restricted to $\textrm{gh}=0$ fields—coincides with the evolution relation in $\overline{\mathcal {F}^\partial _\textrm{in}}\times \mathcal {F}^\partial _\textrm{out}$ for Chern–Simons theory on the cylinder $I\times \Sigma$.

##### Proof

We are restricting our attention only the to $\textrm{gh}=0$ connection field $\textsf{A}+dt\cdot a$ with $\textsf{A}$ a *t*-dependent 1-form on $\Sigma$ and *a* a *t*-dependent 0-form on $\Sigma$ (both are $\mathcal {G}$-valued). The equation of motion—zero-curvature condition—$F_{\textsf{A}+dt\, a}=0$ splits into

74 $$\begin{aligned}&d_\Sigma \textsf{A}+ \frac{1}{2} [\textsf{A},\textsf{A}] = 0, \end{aligned}$$

75 $$\begin{aligned}&\partial _t \textsf{A}= (d_\Sigma + [\textsf{A},-]) a . \end{aligned}$$

Equation (75) says that $\textsf{A}$ changes by a continuous gauge transformation on $\Sigma$ as *t* changes, with *a* the infinitesimal generator. Thus,

76 $$\begin{aligned} \textsf{A}|_{t=1}=g\,\textsf{A}|_{t=0}\, g^{-1} + g d_\Sigma g^{-1},\quad \textrm{with}\;\; g=P\overleftarrow{\exp } \left( -\int _0^1 dt\, a\right) . \end{aligned}$$

This implies that we can recover the (1, 0) component of $\textsf{A}$ at $t=0$ from its known value at $t=1$ and can recover the (0, 1) component at $t=1$ from its known value at $t=0$. Thus,

77 $$\begin{aligned} \textsf{A}|_{t=1}&= \textsf{A}^{1,0}_\textrm{out}+\textsf{A}^{0,1}|_{t=1}=\textsf{A}^{1,0}_\textrm{out}+ g \textsf{A}^{0,1}_\textrm{in}g^{-1} + g \bar{\partial }g^{-1} ,\end{aligned}$$

78 $$\begin{aligned} \textsf{A}|_{t=0}&= \textsf{A}^{0,1}_\textrm{in}+\textsf{A}^{1,0}|_{t=0}=\textsf{A}^{0,1}_\textrm{in}+ g^{-1} \textsf{A}^{1,0}_\textrm{out}g + g^{-1} \partial g . \end{aligned}$$

Note that these two equations coincide with the first two equations in (72). Next, Eq. (74) means that the curvature of $\textsf{A}$ must vanish on $\Sigma \times \{t\}$ for any *t*. In fact, it suffices to verify it just for one value of *t*, because for all others it would follow from (75). Checking (74) at $t=1$, we have

79 $$\begin{aligned} F_\textsf{A}\big |_{t=1}=\underbrace{\bar{\partial }\textsf{A}^{1,0}_\textrm{out}+ \partial (g \textsf{A}^{0,1}_\textrm{in}g^{-1}-\bar{\partial }g\cdot g^{-1})}_{d_\Sigma \textsf{A}|_{t=1}} + [\textsf{A}^{1,0}_\textrm{out}, g \textsf{A}^{0,1}_\textrm{in}g^{-1}-\bar{\partial }g\cdot g^{-1}] \quad =0 .\qquad \end{aligned}$$

This equation coincides with the constraint $Y=0$ in (72) coming from equating to zero the variation of the generating function $\mathbb {I}$ in the auxiliary parameter *g*.

Thus, we have checked that the lagrangian in the boundary phase space induced from the equations of motion (the evolution relation) coincides with the lagrangian generated by $\mathbb {I}$. $\square$

##### Remark 5.18

The function $\mathbb {I}$ given by (71) is also the Hamilton–Jacobi action (see [14, Section 7.2]): it is the evaluation of the Chern–Simons action with polarization terms, restricted to degree-zero fields,

$$\begin{aligned} S^{f}_\textrm{ph}= & {} \int _{ I\times \Sigma } \Big (\frac{1}{2} \langle A, dA \rangle +\frac{1}{6} \langle A, [A,A] \rangle \Big ) + \int _{\{1\}\times \Sigma } \frac{1}{2} \langle \textsf{A}^{1,0},\textsf{A}^{0,1} \rangle \\ {}{} & {} - \int _{\{0\}\times \Sigma } \frac{1}{2} \langle \textsf{A}^{0,1},\textsf{A}^{1,0} \rangle , \end{aligned}$$

on any connection 1-form *A* solving the “evolution equation” $\iota _{\frac{\partial }{\partial t}} F_A=0$ subject to boundary conditions $(A|_{t=1})^{1,0}=\textsf{A}^{1,0}_\textrm{out}$, $(A|_{t=0})^{0,1}=\textsf{A}^{0,1}_\textrm{in}$ and with the parallel transport of *A* along the interval $I\times \{z\}$ given by $g(z)\in G$ for any $z\in \Sigma$. One proves this by an explicit computation similar to the proof of Lemma 5.14, picking a convenient gauge equivalent representative for $A=\textsf{A}+a\,dt$ with *a* constant along *I* (but allowed to vary in $\Sigma$ direction). Here we are using gauge-invariance of Chern–Simons action $\bmod \,4\pi ^2\mathbb {Z}$ with respect to gauge transformations trivial on the boundary.

#### Quantum master equation

Quantum BFV operators on in- and out-states $\Omega _\textrm{in},\Omega _\textrm{out}$ are given by canonical quantization of the boundary BFV action

$$\begin{aligned} S^\textrm{BFV}_\Sigma = \pm \int _\Sigma \langle c,F_\textsf{A}\rangle +\langle \textsf{A}^* ,\frac{1}{2}[c,c]\rangle \end{aligned}$$

with ± corresponding to out-/in-boundary. Explicitly, quantum BFV operators are^45^

80 $$\begin{aligned} \Omega _\textrm{out}&= \int _\Sigma \left\langle c_\textrm{out},\bar{\partial }\textsf{A}^{1,0}_\textrm{out}-i\hbar (\partial +[\textsf{A}^{1,0}_\textrm{out},-])\frac{\delta }{\delta \textsf{A}^{1,0}_\textrm{out}} \right\rangle -i\hbar \left\langle \frac{1}{2} [c_\textrm{out},c_\textrm{out}],\frac{\delta }{\delta c_\textrm{out}} \right\rangle , \end{aligned}$$

81 $$\begin{aligned} \Omega _\textrm{in}&= \int _\Sigma \left\langle c_\textrm{in},-\partial \textsf{A}^{0,1}_\textrm{in}-i\hbar (\bar{\partial }+[\textsf{A}^{0,1}_\textrm{in},-])\frac{\delta }{\delta \textsf{A}^{0,1}_\textrm{in}} \right\rangle -i\hbar \left\langle \frac{1}{2} [c_\textrm{in},c_\textrm{in}],\frac{\delta }{\delta c_\textrm{in}} \right\rangle . \end{aligned}$$

##### Lemma 5.19

The partition function $Z=e^{\frac{i}{\hbar }S^\textrm{eff}}$ with $S^\textrm{eff}$ given by (61), (63) satisfies the modified quantum master equation

82 $$\begin{aligned} (\Omega _\textrm{out}+\Omega _\textrm{in}-\hbar ^2\Delta _\textrm{res})Z=0 \end{aligned}$$

with $\Delta _\textrm{res}=\int _\Sigma \langle \frac{\delta }{\delta \sigma },\frac{\delta }{\delta \textsf{A}^*_\textrm{res}} \rangle$ the BV Laplacian on residual fields.

##### Proof

Given the ansatz $Z=e^{\frac{i}{\hbar }S^\textrm{eff}}$, the equation (82) can be written as

83 $$\begin{aligned} Z^{-1}\Omega _\textrm{in}Z + Z^{-1}\Omega _\textrm{out}Z +\frac{1}{2} \{S^\textrm{eff},S^\textrm{eff}\}_\textrm{res}-i\hbar \Delta _\textrm{res}S^\textrm{eff} {\mathop {=}\limits ^{!}} 0 \end{aligned}$$

with $\{,\}_\textrm{res}$ the odd Poisson bracket on residual fields associated with the symplectic structure (68). Moreover, using the decomposition $S^\textrm{eff}=S^{\textrm{eff}\,(0)}-i\hbar \,\mathbb {W}(\sigma )$, the mQME can be further rewritten as

84 $$\begin{aligned}{} & {} Z^{-1}\Omega _\textrm{in}Z + Z^{-1}\Omega _\textrm{out}Z +\frac{1}{2} \{S^{\textrm{eff}\,(0)},S^{\textrm{eff}\,(0)}\}_\textrm{res}\nonumber \\{} & {} \quad -\,i\hbar \Big ( \{S^{\textrm{eff}\,(0)},\mathbb {W}\}_\textrm{res}+ \Delta _\textrm{res}S^{\textrm{eff}\,(0)}\Big ) {\mathop {=}\limits ^{!}} 0 . \end{aligned}$$

It is easiest to compute the term $\frac{1}{2} \{S^{\textrm{eff}\,(0)},S^{\textrm{eff}\,(0)}\}_\textrm{res}$ using $(g,g^*)$ - parametrization of residual fields. We have

$$\begin{aligned} S^{\textrm{eff}\,(0)}= & {} \mathbb {I}+\int _\Sigma \langle g^*, c_\textrm{out}g- g c_\textrm{in}\rangle , \\ \frac{\delta }{\delta g}S^{\textrm{eff}\, (0)}= & {} -g^{-1} Y+g^* c_\textrm{out}+c_\textrm{in}g^* ,\\ \frac{\delta }{\delta g^*} S^{\textrm{eff}\,(0)}= & {} c_\textrm{out}g-g c_\textrm{in}, \end{aligned}$$

with $\mathbb {I}$ as in (71) and *Y* as in (73). Thus,

$$\begin{aligned} \frac{1}{2} \{S^{\textrm{eff}\,(0)},S^{\textrm{eff}\,(0)}\}_\textrm{res}= & {} S^{\textrm{eff}\,(0)}\left( \int _\Sigma \langle \frac{\overleftarrow{\delta }}{\delta g}, \frac{\overrightarrow{\delta }}{\delta g^*}\rangle \right) S^{\textrm{eff}\,(0)} \\= & {} \int _\Sigma \langle c_\textrm{out}-g c_\textrm{in}g^{-1},-Y+gg^* c_\textrm{out}+g c_\textrm{in}g^* \rangle . \end{aligned}$$

Acting on the partition function with the boundary BFV operators yields

$$\begin{aligned} Z^{-1}\Omega _\textrm{out}Z= & {} \int _\Sigma \Big \langle c_\textrm{out},\bar{\partial }\textsf{A}^{1,0}_\textrm{out}+ (\partial +[\textsf{A}^{1,0}_\textrm{out},-])(g \textsf{A}^{0,1}_\textrm{in}g^{-1}-\bar{\partial }g \; g^{-1}) \Big \rangle \\ {}{} & {} -\frac{1}{2} \langle [c_\textrm{out},c_\textrm{out}],gg^* \rangle , \\ Z^{-1}\Omega _\textrm{in}Z= & {} \int _\Sigma \Big \langle c_\textrm{in},-\partial \textsf{A}^{0,1}_\textrm{in}- (\bar{\partial }+[\textsf{A}^{0,1}_\textrm{in},-])(g^{-1} \textsf{A}^{1,0}_\textrm{out}g+ g^{-1}\partial g ) \Big \rangle \\ {}{} & {} +\frac{1}{2} \langle [c_\textrm{in},c_\textrm{in}],g^* g \rangle . \end{aligned}$$

Putting together these computations, we find that

85 $$\begin{aligned} Z^{-1}\Omega _\textrm{in}Z + Z^{-1}\Omega _\textrm{out}Z +\frac{1}{2} \{S^{\textrm{eff}\,(0)},S^{\textrm{eff}\,(0)}\}_\textrm{res}= 0 \end{aligned}$$

—all the terms in this combination cancel out. This gives us the mQME in the leading order $O(\hbar ^0)$.

For the two remaining terms, $\{S^{\textrm{eff}\,(0)},\mathbb {W}\}_\textrm{res}$ and $\Delta _\textrm{res}S^{\textrm{eff}\,(0)}$, we will use the $(\sigma ,\textsf{A}^*_\textrm{res})$-parametrization for residual fields. The variation of $j(\sigma )$ [see (60)] in $\sigma$ is

$$\begin{aligned} \delta _\sigma j(\sigma ) =\textrm{tr}_\mathcal {G}P(\textrm{ad}_\sigma )\textrm{ad}_{\delta \sigma },\qquad \textrm{where}\quad P(x)=\frac{1}{2} \coth \frac{x}{2}-\frac{1}{x} . \end{aligned}$$

Therefore, using (62), we have

86 $$\begin{aligned} \{S^{\textrm{eff}\,(0)},\mathbb {W}\}_\textrm{res}= & {} \sum _{z\in \Sigma } \textrm{tr}_\mathcal {G}P(\textrm{ad}_\sigma )[-F_+(\textrm{ad}_\sigma )\circ c_\textrm{out}- F_-(\textrm{ad}_\sigma )\circ c_\textrm{in},\;\bullet ] \nonumber \\= & {} \sum _{z\in \Sigma } \textrm{tr}_\mathcal {G}P(\textrm{ad}_\sigma )[- c_\textrm{out}+ c_\textrm{in},\;\bullet ] . \end{aligned}$$

Here the last simplification relies on the identity

87 $$\begin{aligned} \textrm{tr}_\mathcal {G}[(\textrm{ad}_x)^a\circ y,(\textrm{ad}_x)^b\bullet ]=0\qquad \textrm{for}\;\; a\ge 1,\; b\ge 0 , \end{aligned}$$

which follows from the cyclic property of the trace and Jacobi identity. We also have

88 $$\begin{aligned} \Delta _\textrm{res}S^{\textrm{eff}\,(0)}{} & {} =\sum _{z\in \Sigma } -\textrm{tr}_\mathcal {G}\sum _{r,s\ge 0} \frac{B_{r+s+1}}{(r+s+1)!} (\textrm{ad}_\sigma )^r \textrm{ad}_\bullet \Big ((-1)^{r+s+1}(\textrm{ad}_\sigma )^s c_\textrm{out}-(\textrm{ad}_\sigma )^s c_\textrm{in}\Big ) \nonumber \\{} & {} = \sum _{z\in \Sigma } \textrm{tr}_\mathcal {G}\sum _{r,s\ge 0} \frac{B_{r+s+1}}{(r+s+1)!} \Big [ (-1)^{r+s+1}(\textrm{ad}_\sigma )^s c_\textrm{out}-(\textrm{ad}_\sigma )^s c_\textrm{in}, (\textrm{ad}_\sigma )^r\bullet \Big ]\nonumber \\{} & {} \underset{(\textrm{87})}{=}\sum _{z\in \Sigma }\textrm{tr}_\mathcal {G}\sum _{r\ge 0}\frac{B_{r+1}}{(r+1)!} [c_\textrm{out}-c_\textrm{in},(\textrm{ad}_\sigma )^r\bullet ]\nonumber \\{} & {} = \sum _{z\in \Sigma } \textrm{tr}_\mathcal {G}\; \textrm{ad}_{c_\textrm{out}-c_\textrm{in}}\circ P(\textrm{ad}_\sigma ). \end{aligned}$$

Comparing (86) and (88), we see that they exactly cancel each other pointwise on $\Sigma$. Thus,

$$\begin{aligned} \{S^{\textrm{eff}\,(0)},\mathbb {W}\}_\textrm{res}+ \Delta _\textrm{res}S^{\textrm{eff}\,(0)}=0 . \end{aligned}$$

Together with (85), this finishes the proof of mQME (84). $\square$

##### Remark 5.20

The check of the mQME above clearly breaks into two parts:

- The classical part
  89 $$\begin{aligned} Z^{-1}\Omega _\textrm{in}Z + Z^{-1}\Omega _\textrm{out}Z +\frac{1}{2} \{S^{\textrm{eff}\,(0)},S^{\textrm{eff}\,(0)}\}=0, \end{aligned}$$
  which is unambiguous and requires no regularization.
- The quantum part
  $$\begin{aligned} \{S^{\textrm{eff}\,(0)},\mathbb {W}\}_\textrm{res}+ \Delta _\textrm{res}S^{\textrm{eff}\,(0)}=0 , \end{aligned}$$
  which makes sense with the same regularization as the one used in the proof of Lemma 5.12: replacing $\Sigma$ with the set of vertices of a triangulation.

*Aside: mQME and Polyakov–Wiegmann formula* The classical part of the mQME, Eq. (89), itself splits into two parts: terms involving the antifield $g^*$ and others. The terms involving $g^*$ cancel due to invariance of the inner product. The cancellation of the remaining terms can be understood in terms of the WZW model as follows. The part of the effective action $\mathbb {I}(\textsf{A}^{0,1}_\textrm{in}, \textsf{A}^{1,0}_\textrm{out};g)$ defined in (71) can be identified as the WZW action coupled to two external chiral gauge fields $\textsf{A}^{0,1}_\textrm{in}, \textsf{A}^{1,0}_\textrm{out}$, see, e.g., Eq. (4.5) in [21, §4.2]. This coupling is sometimes called “gauging the $G_L \times G_R$ symmetry”, for instance in [38]. For us it is more natural to call it the $G_\textrm{in}\times G_\textrm{out}$-action. Explicitly, the action of $(h_\textrm{in},h_\textrm{out}) \in G \times G$ on $(\textsf{A}^{0,1}_\textrm{in},\textsf{A}^{1,0}_\textrm{out};g)$ is

90 $$\begin{aligned} (h_\textrm{in},h_\textrm{out})\cdot (\textsf{A}^{0,1}_\textrm{in},\textsf{A}^{1,0}_\textrm{out};g) = \left( {}^{h_\textrm{in}}(\textsf{A}^{0,1}_\textrm{in}),{}^{h_\textrm{out}}(\textsf{A}^{1,0}_\textrm{out});h_\textrm{out}g h_\textrm{in}^{-1}\right) . \end{aligned}$$

It is well known that under the transformation (90) $\mathbb {I}$ is not invariant, but transforms according to the Polyakov–Wiegmann [34] formula:

91 $$\begin{aligned} \mathbb {I}\left( {}^{h_\textrm{in}}(\textsf{A}^{0,1}_\textrm{in}),{}^{h_\textrm{out}}(\textsf{A}^{1,0}_\textrm{out});h_\textrm{out}gh_\textrm{in}^{-1}\right)= & {} \mathbb {I}(\textsf{A}^{0,1}_\textrm{in}, \textsf{A}^{1,0}_\textrm{out};g) - \mathbb {I}(\textsf{A}^{0,1}_\textrm{in},0;h_\textrm{in}) \nonumber \\ {}{} & {} + \mathbb {I}(0, \textsf{A}^{1,0}_\textrm{out};h_\textrm{out}). \end{aligned}$$

We claim that this equation is just the finite version of the classical part of the mQME, Eq. (89) above. To see this, consider a path $(h_\textrm{in}(t),h_\textrm{out}(t))$ of gauge transformations starting at the identity and compute the derivative of (91) at $t=0$. The computation is quite straightforward and we just sketch it: using Eq. (73), we get

$$\begin{aligned} \left. \frac{d}{dt}\right| _{t=0}\mathbb {I}\left( \textsf{A}^{0,1}_\textrm{in},\textsf{A}^{1,0}_\textrm{out},h_\textrm{out}(t)gh_\textrm{in}(t)^{-1}\right) = \int _\Sigma \langle \dot{h}_\textrm{out}- g \dot{h}_\textrm{in}g^{-1},Y\rangle . \end{aligned}$$

Upon identifying $c_\textrm{in}= \dot{h}_\textrm{in}, c_\textrm{out}= \dot{h}_\textrm{out}$ this gives the piece of $\frac{1}{2} \{S^{\textrm{eff}\,(0)},S^{\textrm{eff}\,(0)}\}$ of (89) not involving $g^*$. Then, we find that

$$\begin{aligned}{} & {} \left. \frac{d}{dt}\right| _{t=0}\mathbb {I}\left( {}^{h_\textrm{in}(t)}(\textsf{A}^{0,1}_\textrm{in}),\textsf{A}^{1,0}_\textrm{out},g\right) + \mathbb {I}(\textsf{A}^{0,1}_\textrm{in},0,h_\textrm{in}(t)) \\{} & {} \quad = - \bar{\partial }_{\textsf{A}^{0,1}_\textrm{in}}\dot{h}_\textrm{in}(g^{-1}A^{1,0}_\textrm{out}g + g^{-1}\partial g) \\{} & {} \qquad - \int \langle \dot{h}_\textrm{in}, Y\big |_{g=1,A^{0,1}_\textrm{out}= 0}\rangle = Z^{-1}\Omega _\textrm{in}Z \bigg |_{g^*=0,c_\textrm{in}= \dot{h}_\textrm{in}, c_\textrm{out}= \dot{h}_\textrm{out}} \end{aligned}$$

and similarly for the action of $h_\textrm{out}$. Overall, we find

$$\begin{aligned}{} & {} \left. \frac{d}{dt}\right| _{t=0}\left[ \mathbb {I}\left( {}^{h_\textrm{in}}(\textsf{A}^{0,1}_\textrm{in}),{}^{h_\textrm{out}}(\textsf{A}^{1,0}_\textrm{out});h_\textrm{out}gh_\textrm{in}^{-1}\right) + \mathbb {I}(\textsf{A}^{0,1}_\textrm{in}, 0, h_\textrm{in}(t)) - \mathbb {I}(0,\textsf{A}^{1,0}_\textrm{out},h_\textrm{out}(t)\right] \\{} & {} \quad =\left[ Z^{-1}\Omega _\textrm{in}Z + Z^{-1}\Omega _\textrm{out}Z +\frac{1}{2} \{S^{\textrm{eff}\,(0)},S^{\textrm{eff}\,(0)}\}\right] _{g^* = 0, c_\textrm{in}= \dot{h}_\textrm{in}, c_\textrm{out}= \dot{h}_\textrm{out}} , \end{aligned}$$

which proves the claim that (a part of) the $\hbar = 0$ part of the mQME is equivalent to the infinitesimal Polyakov–Wiegmann formula. We will comment further on the relationship between Chern–Simons theory on $\Sigma \times I$ and WZW theory on $\Sigma$ in Sect. 5.3.8 below.

##### Remark 5.21

In the mQME (82) and the proof above we were using the $(g,\textsf{A}^*_\textrm{res})$-parametrization of residual fields for the BV Laplacian. The corresponding statement for the BV Laplacian in $(g,g^*)$-parametrization,

$$\begin{aligned} \Delta _{[g,g^*]}=\int _\Sigma \langle \frac{\delta }{\delta g} , \frac{\delta }{\delta g^*}\rangle = \int _\Sigma \langle \frac{\delta }{\delta g\; g^{-1}} , \frac{\delta }{g\, \delta g^*}\rangle , \end{aligned}$$

is:

92 $$\begin{aligned} (\Omega _\textrm{out}+\Omega _\textrm{in}-\hbar ^2\Delta _{[g,g^*]})\;e^{\frac{i}{\hbar }S^{\textrm{eff}\,(0)}}=0 . \end{aligned}$$

Note that here we should not be including the $-i\hbar \mathbb {W}$ term in the effective action, cf. Remark 5.16. The proof of (92) is exactly as before in the order $O(\hbar ^0)$. In the order $O(\hbar ^1)$, we have

$$\begin{aligned} \Delta _{[g,g^*]} S^{\textrm{eff}\, (0)}=\sum _{z\in \Sigma } \frac{1}{2} \textrm{div}_{T^*[-1]G}\big \{\langle g^*,c_\textrm{out}g-g c_\textrm{in}\rangle ,\bullet \big \} . \end{aligned}$$

The hamiltonian vector field generated by the ghost term in the effective action is the cotangent lift to $T^*[-1]G$ of the vector field

$$\begin{aligned} X=\langle c_\textrm{out},\frac{\partial }{\partial g\; g^{-1}} \rangle -\langle c_\textrm{in},\frac{\partial }{g^{-1}\partial g} \rangle . \end{aligned}$$

—This is a sum of a right-invariant and a left-invariant vector field on *G*. Since the Haar measure is bi-invariant, *X* has divergence zero. Therefore,

$$\begin{aligned} \Delta _{[g,g^*]} S^{\textrm{eff}\, (0)}=\sum _{z\in \Sigma } \textrm{div}_G X = 0 . \end{aligned}$$

Ultimately, to avoid the ambiguity as to whether we should be including the term $-i\hbar \mathbb {W}$ into the partition function or not, we can use the invariant formulation where the partition function is a *half-density* (rather than a function) on residual fields and the BV Laplacian is the canonical BV Laplacian on half-densities. Then the mQME is

$$\begin{aligned} (\Omega _\textrm{out}+\Omega _\textrm{in}-\hbar ^2 \Delta _\textrm{res}^\textrm{can})\,Z^\textrm{can}=0 , \end{aligned}$$

where $Z^\textrm{can}=e^{\frac{i}{\hbar }S^\textrm{eff}_{[x,\xi ]}}d^{\frac{1}{2}}x D^{\frac{1}{2}}\xi$. Here $(x,\xi )$ can be any Darboux coordinate system on $\mathcal {V}$, e.g., $(\sigma ,\textsf{A}^*_\textrm{res})$ or $(g,g^*)$.

*Summary* Summarizing the main results of Sect. 5.3, we have the following:

- The canonical partition function of the nonabelian theory on the cylinder $[0,1]\times \Sigma$ with the “parallel ghost” polarization is: $Z^\textrm{can}=e^{\frac{i}{\hbar }S^\textrm{eff}_{[g,g^*]}} \mathcal {D}^{\frac{1}{2}} g\, \mathcal {D}^{\frac{1}{2}} g^*$ where the effective action relative to the coordinate system $(g,g^*)$ on $\mathcal {V}$ is given explicitly by
  93 $$\begin{aligned}{} & {} S^\textrm{eff}_{[g,g^*]}\nonumber \\{} & {} \quad =\int _\Sigma \Big ( \langle \textsf{A}^{1,0}_\textrm{out}, g \textsf{A}^{0,1}_\textrm{in}g^{-1} \rangle - \langle \textsf{A}^{1,0}_\textrm{out},\bar{\partial }g\cdot g^{-1} \rangle - \langle \textsf{A}^{0,1}_\textrm{in}, g^{-1} \partial g \rangle \Big ) +\textrm{WZW}(g) \nonumber \ \\{} & {} \qquad +\, \int _\Sigma -\langle c_\textrm{out},g\, g^* \rangle + \langle c_\textrm{in}, g^* g \rangle . \end{aligned}$$
  In particular, there are no quantum corrections in $S^\textrm{eff}_{[g,g^*]}$.
- $Z^\textrm{can}$ satisfies the modified quantum master equation.
- The restriction of $S^\textrm{eff}_{[g,g^*]}$ to ghost number zero fields is the Hamilton–Jacobi action, i.e., is the generalized generating function for the evolution relation of the classical theory obtained by evaluating the classical action on a solution of the evolution equations, see Sect. 2.

##### Remark 5.22

The relation between 3D nonabelian Chern–Simons theory and the(gauged) WZW model was studied from different angles in the literature. The closest discussion to ours, perhaps, was in [9]: *G*/*G* WZW theory was recovered from Chern–Simons on a cylinder, using essentially the same gauge fixing and polarization as the ones we employ. But there are crucial differences in the two approaches. We have an explicit Feynman diagram computation of the partition function and prove the QME and the gauge invariance property at the quantum level. In [9], on the other hand, quantum gauge invariance was assumed and was used to evaluate the Chern–Simons partition function.

#### “Vertical” Wilson lines

One can enrich Chern–Simons theory with Wilson line observables given classicaly by the parallel transport of the connection field *A* along a curve $\gamma$ ending on the boundary; the parallel transport is evaluated in some linear representation $\rho$ of *G* on a vector space *R*.

**Fig. 7:**
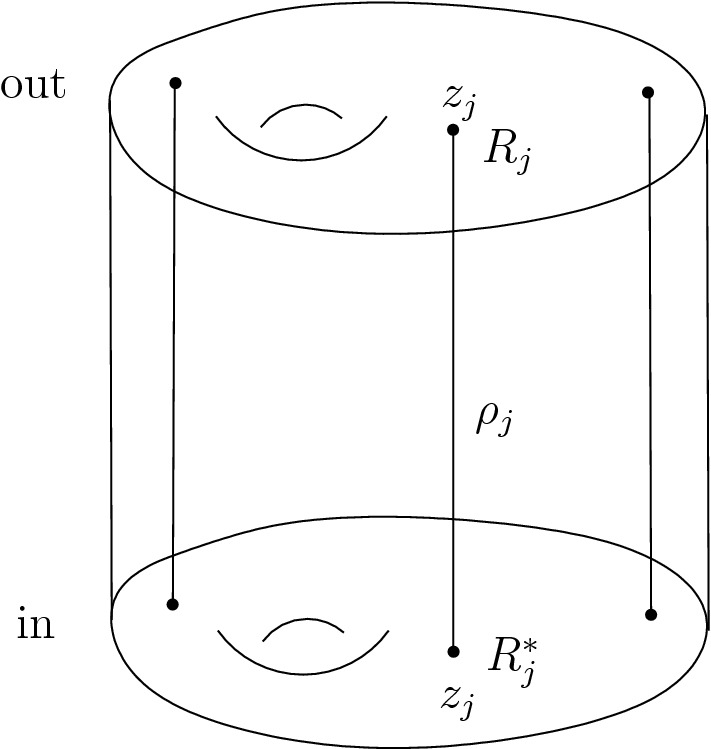
Vertical Wilson lines

Let us consider a very simple case: several “vertical” Wilson lines with $\gamma _j=I\times \{z_j\}$ connecting the in- and out-boundaries of the cylinder $I\times \Sigma$; here $z_j$ are a collection of points on $\Sigma$, $j=1,\ldots ,n$. We are fixing representations $\rho _j$ for the Wilson lines, with $R_j$ the respective representation spaces (Fig. 7).

Note that for our choice of gauge fixing, we have for the Wilson lines

$$\begin{aligned} W_j=\rho _j(P\overleftarrow{\exp }(-\int _{\gamma _j} A)) = \rho _j(e^{-\sigma (z_j)})= \rho _j(g(z_j)) \qquad \in \textrm{End}(R_j) . \end{aligned}$$

i.e., vertical Wilson lines depend only on the residual fields.

Thus, the partition function of the theory enriched with vertical Wilson lines is:

94 $$\begin{aligned} Z_{ I\times \Sigma , \{\gamma _j\}} = Z_{ I\times \Sigma } \cdot \bigotimes _j \rho _j(g(z_j)) \end{aligned}$$

with $Z_{ I\times \Sigma }=e^{\frac{i}{\hbar }S^\textrm{eff}}$ the partition function without the Wilson lines.

The space of out-states is given by functionals of $\textsf{A}^{1,0}_\textrm{out}, c_\textrm{out}$ with values in $\bigotimes _j R_j$, while the space of in-states is given by functionals of $\textsf{A}^{0,1}_\textrm{in}, c_\textrm{in}$ also with values in $\bigotimes _j R_j^*$.^46^ The BFV operators are^47^:

95 $$\begin{aligned} \Omega _\textrm{out}^{\Sigma ,\{z_j\}} = \Omega _\textrm{out}^\Sigma + i\hbar \sum _j \rho _j(c_\textrm{out}(z_j)) \;\;, \quad \Omega _\textrm{in}^{\Sigma ,\{z_j\}} = \Omega _\textrm{in}^\Sigma + i\hbar \sum _j \rho _j^*(c_\textrm{in}(z_j)) , \end{aligned}$$

where $\Omega _\textrm{out}^\Sigma$, $\Omega _\textrm{in}^\Sigma$ are the BFV operators for the theory without the Wilson lines, given by (80), (81); $\rho _j^*$ is the dual representation to $\rho _j$ with representation space $R_j^*$.

As a direct consequence of Lemma 5.19, one has that the partition function with Wilson lines (94) satisfies the modified quantum master equation:

$$\begin{aligned} (\Omega _\textrm{out}^{\Sigma ,\{z_j\}}+\Omega _\textrm{in}^{\Sigma ,\{z_j\}}-\hbar ^2 \Delta _\textrm{res})\, Z_{ I\times \Sigma , \{\gamma _j\}} =0 . \end{aligned}$$

Here we understand that the $\rho _j^*$ term in $\Omega _\textrm{in}$ acts on the second factor in $\rho _j(g(z_j))\in R_j\otimes R_j^*$, while the $\rho _j$ term in $\Omega _\textrm{out}$ acts on the first factor.

#### The CS-WZW correspondence: WZW theory as an effective theory of Chern–Simons

Equation (93) is evidence of a strong relationship between the Chern–Simons theory on a manifold with boundary $\Sigma$ and the WZW theory on the Riemann surface $\Sigma$. This relationship has, of course, already been subject to a lot of scrutiny after Witten’s seminal article [37]. In the approach of this paper, this relationship stems from the fact that the gauged WZW action emerges as an effective action of the Chern–Simons theory, as is clear from Eq. (93). To be precise, the following two theories are equivalent:

(i) The BV-BFV effective theory of Chern–Simons on a $I\times \Sigma$, restricted to the gauge-fixing lagrangian $\mathcal {L}= \{g^* = 0\} \subset T^*[-1]G$.
(ii) The WZW theory with gauged “$G_\textrm{in}\times G_\textrm{out}$”-symmetry.

This is a very strong statement of equivalence: It means that essentially all quantities computed from the action functional in gauged WZW theory have an expression in Chern–Simons theory. We summarize this relationship in Table 1 below.

**Table 1:** The CS-WZW correspondence

Object in CS on $I\times \Sigma$	Object in gauged WZW on $\Sigma$
Effective action $S^\textrm{eff}_{[g,g*]}$	Gauged WZW action $\mathbb {I}(\textsf{A}^{0,1}_\textrm{in},\textsf{A}^{1,0}_\textrm{out};g)$
mQME $(\Omega - \Delta _\textrm{res})Z = 0$	Polyakov–Wiegmann formula (91) (group 1-cocycle property)
Expectation value *W* of Wilson line $\gamma = I\times \{z\}$ in rep. $\rho$	Field insertion $\rho (g(z))$

##### Remark 5.23

One might wonder why in Table 1 on the left hand side we have objects defined in the quantization on the Chern–Simons theory, while on the right-hand side we have entirely classical objects in the WZW model. This apparent puzzle is resolved by the observation that on the left-hand side we are seeing only the semiclassical limit of the quantum Chern–Simons theory (which in this case happens to be exact, since there are no loop contributions).^48^

##### Remark 5.24

(*Nonequivalent gauge-fixing Lagrangians*). Instead of $\mathcal {L}= \{g^* = 0\}$, one could restrict the effective Chern–Simons action (93) also to another lagrangian $\mathcal {L}'\subset T^*[-1]G$ given by $g=1$. In that way, one obtains

96 $$\begin{aligned} S^\textrm{eff}_{[g,g^*]}\bigg |_{g=1} = \int _\Sigma \langle \textsf{A}^{1,0}_\textrm{out},\textsf{A}^{0,1}_\textrm{in}\rangle + \int _\Sigma \langle g^*, c_\textrm{in}- c_\textrm{out}\rangle . \end{aligned}$$

Upon integrating *Z* over $\mathcal {L}$ or $\mathcal {L'}$, one obtains two $(\Omega _\textrm{in}+ \Omega _\textrm{out})$-cocycles $Z_1$, $Z_2$. $Z_1$ is concentrated in ghost degree 0 (we will discuss it in more detail in the next subsection) while

$$\begin{aligned} Z_2 = \exp \left( \frac{i}{\hbar }\int _\Sigma \langle \textsf{A}^{1,0}_\textrm{out},\textsf{A}^{0,1}_\textrm{in}\rangle \right) \delta ( c_\textrm{in}- c_\textrm{out}) \end{aligned}$$

has nonzero ghost number (formally, it is infinite, $\textrm{gh}=\dim \Omega ^0(\Sigma ,\mathfrak {g})$). Therefore, $\mathcal {L}$ and $\mathcal {L}'$ provide an example of nonequivalent gauge-fixing Lagrangians.

In the $(g,g^*)$-coordinates we can define a particularly simple gauge-fixing lagrangian $\mathcal {L}$ given by $g^* = 0$ (for the general remarks in this section, we will allow ourselves to ignore issues arising from possible zero modes).

97 $$\begin{aligned} S^\textrm{eff}\big |_\mathcal {L}= & {} \textrm{WZW}(g) + \langle \textsf{A}^{1,0}_\textrm{out}, g \textsf{A}^{0,1}_\textrm{in}g^{-1} \rangle - \langle \textsf{A}^{1,0}_\textrm{out},\bar{\partial }g\cdot g^{-1} \rangle - \langle \textsf{A}^{0,1}_\textrm{in}, g^{-1} \partial g \rangle \nonumber \\ {}\equiv & {} \mathbb {I}[\textsf{A}^{1,0}_\textrm{out},\textsf{A}^{0,1}_\textrm{in};g]. \end{aligned}$$

Here $\mathbb {I}[\textsf{A}^{1,0}_\textrm{out},\textsf{A}^{0,1}_\textrm{in};g]$ is the standard way of gauging the WZW action, see, e.g., eq. (4.5) in [21, Sect. 4.2]. We can then express the Chern–Simons partition function on $I\times \Sigma$ as

98 $$\begin{aligned} Z_{I\times \Sigma }[\textsf{A}^{1,0}_\textrm{out},\textsf{A}^{0,1}_\textrm{in}, c_\textrm{in},c_\textrm{out}] \equiv Z_{I\times \Sigma }[\textsf{A}^{1,0}_\textrm{out},\textsf{A}^{0,1}_\textrm{in}] = \int _g \exp \frac{i}{\hbar } \mathbb {I}[\textsf{A}^{1,0}_\textrm{out},\textsf{A}^{0,1}_\textrm{in};g]\mathcal {D}g\qquad \end{aligned}$$

(notice the partition function does not depend on $c_\textrm{in},c_\textrm{out}$). This is the definition of the partition function $Z_\textsf{A}^\textrm{WZW}$ of gauged WZW, see, e.g., eq. (4.7) in [21, Sect. 4.2]. Here we abbreviate $\textsf{A}= (\textsf{A}^{1,0}_\textrm{out}, \textsf{A}^{0,1}_\textrm{in})$. Similarly, we see that a correlator in the gauged WZW theory can be expressed as the partition function of Chern–Simons theory enriched with Wilson lines:

99 $$\begin{aligned}{} & {} \langle \rho _1(g(z_1))\otimes \ldots \otimes \rho _n (g(z_n))\rangle _\textsf{A}\nonumber \\{} & {} \quad = \int _g \rho _1(g(z_1))\otimes \ldots \otimes \rho _n(g(z_n)) e^{\frac{i}{\hbar }\mathbb {I}[g,\textsf{A}]}\mathcal {D}g = \int _g Z_{I\times \Sigma ,\{\gamma _j\}} \mathcal {D}g. \end{aligned}$$

For the purposes of this subsection, we will treat the path integral expressions on the right hand side of (98) and (99) heuristically. In the literature, these objects are typically defined via representation theory. In this paper, we are typically interpreting path integral expressions as defined via Feynman graphs and rules, but for WZW the absence of a natural linear structure on the target (the group *G*) obstructs the treatment of the path integral as a perturbed Gaussian integral. We will therefore simply assume that the partition function exists and defines an element in $\Omega$-cohomology. In [1] it was shown that in genus 0, the $\Omega$-cohomology with *n* Wilson lines ending on the boundary can be identified with the *n*-point space of conformal blocks. We expect this to hold for all genera, and assume it for the purpose of the next section. We summarize the content of the CS-WZW correspondence after integrating over $\mathcal {L}$ in Table 2.

**Table 2:** The CS-WZW correspondence after integrating over the gauge-fixing Lagrangian $\mathcal {L}$

Object in CS on $I\times \Sigma$	Object in gauged WZW on $\Sigma$
CS partition function $Z_{I\times \Sigma }[\textsf{A}^{1,0}_\textrm{out},\textsf{A}^{0,1}_\textrm{in}]$	Gauged WZW partition function $Z_\textsf{A}^\textrm{WZW}$
Expectation value *W* of *n* Wilson lines $\gamma = I\times \{z_i\}$ in rep. $\rho _1,\ldots \rho _n$	Gauged WZW correlator $\langle \rho _1(g(z_1))\cdots \rho _n (g(z_n))\rangle _\textsf{A}$
$\Omega$-cohomology with *n* Wilson lines	*n*-point space of conformal blocks

#### An application: holomorphic factorization of the WZW theory

We will now discuss an application of the correspondence observed above. The arguments in this section will be more heuristic in nature.

**Fig. 8:**
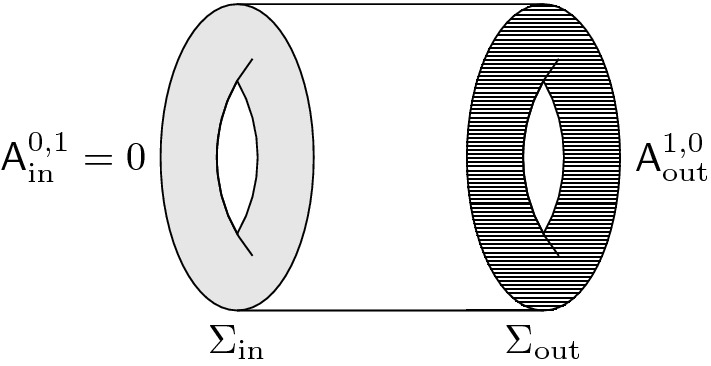
A chiral cylinder: antiholomorphic boundary conditions on $\Sigma _\textrm{in}$, $\textsf{A}^{1,0}_\textrm{out}$-representation on $\Sigma _\textrm{out}$. Gray indicates that we fix a boundary condition on this boundary, while hatching indicates we fix only the polarization

Suppose we fix the boundary condition on one side, e.g., fix the antiholomorphic boundary condition at the $\textrm{in}$-boundary by setting $\textsf{A}^{0,1}_\textrm{in}= 0$ (remember that in our treatment of boundary conditions, setting $\textsf{A}^{0,1}_\textrm{in}= 0$ means that $\textsf{A}\big |_{\Sigma _\textrm{in}} \in \Omega ^{1,0}(\Sigma ,\mathfrak {g})$ ), and take the $\textrm{out}$-boundary in the $\textsf{A}^{1,0}_\textrm{out}$-representation. We will call such a cylinder a “chiral cylinder.” See Fig. 8

**Fig. 9:**
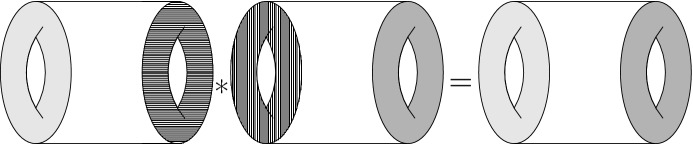
Gluing a chiral and an antichiral cylinder into a cylinder with opposite chiral boundary conditions. Gray indicates a fixed boundary condition, hatching indicates a polarized boundary

After integrating out *g*, we obtain the partition function $\psi (\textsf{A}_{\textrm{out}}^{1,0})$ of a “chiral gauged WZW theory,” i.e., a WZW theory coupled to a chiral gauge field, see, e.g., [38].^49^ This partition function is not a number, but rather a holomorphic gauge invariant section of a line bundle over the space of connections on $\Sigma$.^50^ We can glue the chiral cylinder to another “antichiral” cylinder with opposite boundary conditions (see Fig. 9).

In this way, we obtain—as explained in [38]—the square of the norm of $\psi$.^51^ Here, the “norm square” should be taken with respect to a well-defined inner product on the $\Omega$-cohomology, i.e., the finite-dimensional moduli space of gauge-invariant holomorphic sections.^52^ On the other hand, from the general principles of the BV-BFV formalism, we will then obtain the partition function of Chern–Simons theory with opposite chiral boundary conditions, which is given by specializing to $\textsf{A}^{1,0}_\textrm{out}= \textsf{A}^{1,0}_\textrm{in}= 0$ in (98):

100 $$\begin{aligned} |\psi |^2 = Z_{I\times \Sigma }^{\textrm{CS}} = Z_{\Sigma }^\textrm{WZW}. \end{aligned}$$

Here on the left-hand side we have the norm-square of the partition function of chiral WZW, in the middle we have Chern–Simons partition function on the cylinder with opposite chiral boundary conditions, while on the right-hand side we have the definition of the WZW partition function. Equation (100) is sometimes called “holomorphic factorization of the WZW model”, because one sees that the partition function of the full WZW model—which does not vary holomorphically on the moduli space of conformal structures—splits into a sum of products of holomorphic and antiholomorphic factors, which do depend (anti)holomorphically on the complex structure. Thus, holomorphic factorization of the WZW model follows from the self-similarity of the Chern–Simons partition function on cylinders.

Using the results of Sect. 5.3.7, in particular Eq. (94), these results for the partition function generalize to correlators in chiral and full WZW. Namely, suppose we are given *n* Wilson lines colored by representations $R_1,\ldots ,R_n$, and let $V = \otimes _j R_j$. Then, the Chern–Simons partition function with Wilson lines on a chiral cylinder $\psi _{\{\gamma _j\}}$ is naturally a degree zero element of $V \otimes \mathcal {H}_\textrm{out}$ with $\mathcal {H}_\textrm{out}$ the space of functionals of $\textsf{A}^{1,0}_\textrm{out},c_\textrm{out}$ with values in $V^*$. Gluing with an antichiral cylinder, we obtain the Chern–Simons partition function with Wilson lines and opposite boundary conditions - the correlator $\langle \rho _1(g(z_1)) \cdots \rho _n(g(z_n)) \rangle \in V \otimes V^*$ in a full WZW model. On the other hand, explicitly computing the BV-BFV gluing we obtain^53^$(\psi ,\overline{\psi }) \in V \otimes V^*$. Here $(\cdot ,\cdot )$ is the inner product on the space on *n*-point conformal blocks. Thus, we obtain the generalization of (100) to the case with Wilson lines:

101 $$\begin{aligned} (\psi _{\{\gamma _j\}},\overline{\psi }_{\{\gamma _j\}}) = Z^{CS}_{I\times \Sigma ,\{\gamma _j\}} = \langle \rho _1(g(z_1))\cdots \rho _n(g(z_n))\rangle . \end{aligned}$$

### 3D nonabelian Chern–Simons theory in holomorphic-to-holomorphic polarization

Next, consider the nonabelian Chern–Simons theory on $\Sigma \times [0,1]$ with polarizations as in Sect. 4.1. The residual fields

$$\begin{aligned} \textsf{A}^+_{I\,\textrm{res}}=\textsf{A}^{0}_{{I\,\textrm{res}}}+\textsf{A}^{1,0}_{{I\,\textrm{res}}},\qquad \textsf{A}^-_\textrm{res}= \textsf{A}^{0,1}_\textrm{res}+ \textsf{A}^2_\textrm{res}\end{aligned}$$

and the gauge fixing are as in Sect. 4.1 (but now all forms are $\mathcal {G}$-valued). We will use the notations $\sigma =\textsf{A}^{0}_{I\,\textrm{res}}$, $\lambda =\textsf{A}^{0,1}_\textrm{res}$ for $\textrm{gh}=0$ residual fields, as in Sect. 4.1.1.

In this subsection we will present only the results; the computations are similar to those of Sect. 5.3.

**Fig. 10:**
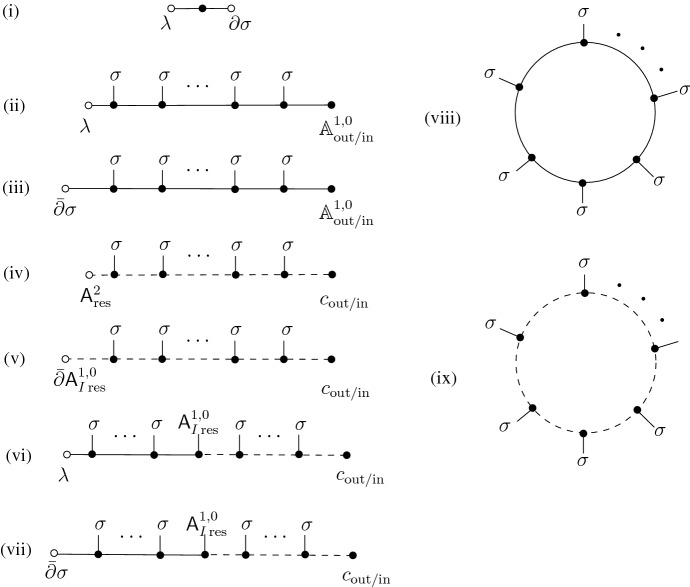
Feynman diagrams in nonabelian theory on a cylinder in holomorphic-to-holomorphic polarization

The Feynman diagrams for the partition function $Z=e^{\frac{i}{\hbar }S^\textrm{eff}}$ are given in Fig. 10:

Here the “physical wheels” (viii) and the “ghost wheels” (ix) cancel each other, due to the form of propagators in the chosen polarization.

Calculating the Feynman diagrams, one finds the following expression for the effective action:

102 $$\begin{aligned} S^\textrm{eff}= S^{\textrm{eff}}_\textrm{ph}+S^{\textrm{eff}}_\textrm{gh}, \end{aligned}$$

where the part depending only on “physical” ($\textrm{gh}=0$) fields (the contribution of diagrams (i), (ii), (iii)) is

103 $$\begin{aligned} S^{\textrm{eff}}_\textrm{ph}= & {} \int _\Sigma \langle \lambda ,\partial \sigma \rangle + \left\langle \textsf{A}^{1,0}_\textrm{out}, \frac{\textrm{ad}_\sigma }{e^{\textrm{ad}_\sigma }-1}\circ \lambda + \left( -\frac{1}{e^{\textrm{ad}_\sigma }-1}+\frac{1}{\textrm{ad}_\sigma } \right) \circ \bar{\partial }\sigma \right\rangle \nonumber \\{} & {} +\,\left\langle \textsf{A}^{1,0}_\textrm{in}, -\frac{\textrm{ad}_\sigma }{1-e^{-\textrm{ad}_\sigma }}\circ \lambda + \left( \frac{1}{1-e^{-\textrm{ad}_\sigma }}-\frac{1}{\textrm{ad}_\sigma } \right) \circ \bar{\partial }\sigma \right\rangle \end{aligned}$$

and the ghost-dependent part [the contribution of diagrams (iv)–(vii)] is

104 $$\begin{aligned} S^{\textrm{eff}}_\textrm{gh}= & {} \int _\Sigma \left\langle c_\textrm{out}, \sum _{k\ge 0}\frac{B_k^-}{k!}(\textrm{ad}_\sigma )^k \textsf{A}^2_\textrm{res}- \sum _{k\ge 0} \frac{B^-_{k+1}}{(k+1)!}(\textrm{ad}_\sigma )^k\bar{\partial }\textsf{A}^{1,0}_{I\,\textrm{res}}\right. \nonumber \\{} & {} \quad \left. +\,\sum _{k,l\ge 0}\frac{B^-_{k+l+1}}{(k+l+1)!}(\textrm{ad}_\sigma )^k\textrm{ad}_{\textsf{A}^{1,0}_{I\,\textrm{res}}}(\textrm{ad}_\sigma )^l \lambda - \sum _{k,l\ge 0} \frac{B^-_{k+l+2}}{(k+l+2)!} (\textrm{ad}_\sigma )^k\textrm{ad}_{\textsf{A}^{1,0}_{I\,\textrm{res}}}(\textrm{ad}_\sigma )^l \bar{\partial }\sigma \right\rangle \nonumber \\{} & {} \quad +\, \left\langle c_\textrm{in}, -\sum _{k\ge 0}\frac{B_k^+}{k!}(\textrm{ad}_\sigma )^k \textsf{A}^2_\textrm{res}+\sum _{k\ge 0} \frac{B^+_{k+1}}{(k+1)!}(\textrm{ad}_\sigma )^k \bar{\partial }\textsf{A}^{1,0}_{I\,\textrm{res}}\right. \nonumber \\{} & {} \quad \left. -\,\sum _{k,l\ge 0} \frac{B^+_{k+l+1}}{(k+l+1)!} (\textrm{ad}_\sigma )^k\textrm{ad}_{\textsf{A}^{1,0}_{I\,\textrm{res}}}(\textrm{ad}_\sigma )^l \lambda +\sum _{k,l\ge 0} \frac{B^+_{k+l+2}}{(k+l+2)!} (\textrm{ad}_\sigma )^k\textrm{ad}_{\textsf{A}^{1,0}_{I\,\textrm{res}}}(\textrm{ad}_\sigma )^l \bar{\partial }\sigma \right\rangle .\nonumber \\ \end{aligned}$$

Here $B_n^\pm$ are the Bernoulli numbers with $B^\pm _1=\pm \frac{1}{2}$ and with $B^+_n=B^-_n$ the usual Bernoulli numbers for $n\ne 1$ (thus, $B^-_n=B_n$ are the standard Bernoulli numbers for $n=0,1,2,\ldots$):

$$\begin{aligned} \begin{array}{c|cccccccc} n &{} 0 &{} 1 &{} 2 &{} 3 &{} 4&{} 5 &{} 6 &{} \cdots \\ \hline B^+_n &{} 1 &{} +\frac{1}{2} &{} \frac{1}{6} &{} 0 &{} -\frac{1}{30} &{} 0 &{} \frac{1}{42} &{} \cdots \\ B^-_n &{} 1 &{} -\frac{1}{2} &{} \frac{1}{6} &{} 0 &{} -\frac{1}{30} &{} 0 &{} \frac{1}{42} &{} \cdots \end{array} \end{aligned}$$

#### Remark 5.25

Another form of the ghost-dependent part of the effective action (104), with sums over *k*, *l* below evaluated explicitly, is:

$$\begin{aligned} S^{\textrm{eff}}_\textrm{gh}= & {} \int _\Sigma \Big \langle c_\textrm{out}, \frac{\textrm{ad}_\sigma }{e^{\textrm{ad}_\sigma }-1}\circ \textsf{A}^2_\textrm{res}+\Big (-\frac{1}{e^{\textrm{ad}_\sigma }-1}+\frac{1}{\textrm{ad}_\sigma } \Big )\circ \bar{\partial }\textsf{A}^{1,0}_{I\,\textrm{res}}\\{} & {} +\,\textrm{ad}_{\textsf{A}^{1,0}_{I\,\textrm{res}}}\frac{1}{e^{\textrm{ad}_\sigma }-1}\circ \lambda -\frac{\textrm{ad}_\sigma }{1-e^{-\textrm{ad}_\sigma }}\textrm{ad}\left( \frac{1-e^{-\textrm{ad}_\sigma }}{\textrm{ad}_\sigma }\circ \textsf{A}^{1,0}_{I\,\textrm{res}}\right) \frac{1}{e^{\textrm{ad}_\sigma }-1}\circ \lambda \\{} & {} -\,\frac{1}{\textrm{ad}_\sigma }\textrm{ad}_{\textsf{A}^{1,0}_{I\,\textrm{res}}}\frac{1}{\textrm{ad}_\sigma }\bar{\partial }\sigma + \frac{1}{e^{\textrm{ad}_\sigma }-1} \textrm{ad}\left( \frac{e^{\textrm{ad}_\sigma }-1}{\textrm{ad}_\sigma }\circ \textsf{A}^{1,0}_{I\,\textrm{res}}\right) \frac{1}{1-e^{-\textrm{ad}_\sigma }}\circ \bar{\partial }\sigma \Big \rangle \\{} & {} +\, \Big \langle c_\textrm{in}, \Big (\sigma \rightarrow -\sigma , \textsf{A}^2_\textrm{res}\rightarrow -\textsf{A}^2_\textrm{res}\Big ) \Big \rangle . \end{aligned}$$

Here the coefficient of $c_\textrm{in}$ is obtained from the coefficient of $c_\textrm{out}$ by replacing $\sigma$ with $-\sigma$ and replacing $\textsf{A}^2_\textrm{res}$ with $-\textsf{A}^2_\textrm{res}$.

Next, one can introduce a new parametrization of the space of residual fields by a group-valued map $$g:\Sigma \rightarrow G$$, a (0, 1)-form $$\Lambda $$, a (1, 0)-form $$\Lambda ^*$$ and a 2-form $$g^*$$^54^:

$$\begin{aligned} g&=e^{-\sigma },\\ \Lambda&= \text{ coefficient } \text{ of } \textsf{A}^{1,0}_\textrm{out} \text{ in } \text{(103) } \\&= \frac{\textrm{ad}_\sigma }{e^{\textrm{ad}_\sigma }-1}\circ \lambda + \left( -\frac{1}{e^{\textrm{ad}_\sigma }-1}+\frac{1}{\textrm{ad}_\sigma } \right) \circ \bar{\partial }\sigma , \\ \Lambda ^*&=\frac{1-e^{-\textrm{ad}_\sigma }}{\textrm{ad}_\sigma }\circ \textsf{A}^{1,0}_{I\,\textrm{res}},\\ g^*&= (\text{ coefficient } \text{ of } c_\textrm{in} \text{ in } \text{(104) } )\cdot g^{-1} . \end{aligned}$$

This change of parametrization has Jacobian 1 and changes one Darboux coordinate system with respect to the BV symplectic form on $\mathcal {V}$ into another one:

$$\begin{aligned} \omega _\textrm{res}=\int _\Sigma \langle \delta \sigma ,\delta \textsf{A}^2_\textrm{res}\rangle + \langle \delta \lambda , \delta \textsf{A}^{1,0}_{I\,\textrm{res}}\rangle = \int _\Sigma \langle \delta g,\delta g^* \rangle + \langle \delta \Lambda ,\delta \Lambda ^* \rangle . \end{aligned}$$

In terms of this new parametrization, the effective action (102) can be written more concisely:

105 $$\begin{aligned} S^\textrm{eff}= & {} - \textrm{WZW}(g^{-1}) -\int _\Sigma \langle \Lambda ,\partial g\cdot g^{-1} \rangle \nonumber \\{} & {} +\,\int _\Sigma \langle \textsf{A}^{1,0}_\textrm{out},\Lambda \rangle + \langle \textsf{A}^{1,0}_\textrm{in},g^{-1}\Lambda g+g^{-1}\bar{\partial }g\rangle +\langle c_\textrm{out}, -g\, g^*+\bar{\partial }\Lambda ^*+[\Lambda ,\Lambda ^*] \rangle \nonumber \\ {}{} & {} + \langle c_\textrm{in},g^*g \rangle , \end{aligned}$$

where $\textrm{WZW}$ is the Wess–Zumino–Witten action defined as in (64).

The effective action (105) satisfies the following properties:

- Its restriction to $\textrm{gh}=0$ fields satisfies the Hamilton–Jacobi property, i.e., it is the generalized generating function for the evolution relation of nonabelian Chern–Simons theory. From this identification one can see that, on-shell, $\Lambda$ can be interpreted as the (0, 1)-component of the connection field at $t=1$.^55^
- One has the modified quantum master equation
  $$\begin{aligned} (\Omega _\textrm{out}+\Omega _\textrm{in}-\hbar ^2\Delta _\textrm{res}) e^{\frac{i}{\hbar } S^\textrm{eff}}=0 \end{aligned}$$
  with the boundary BFV operators
  $$\begin{aligned} \Omega _{\textrm{out}/\textrm{in}}= \int _\Sigma \big \langle c,\pm \bar{\partial }\textsf{A}^{1,0} -i\hbar (\partial +[\textsf{A}^{1,0},-])\frac{\delta }{\delta \textsf{A}^{1,0}} \big \rangle -i\hbar \big \langle \frac{1}{2} [c,c],\frac{\delta }{\delta c} \big \rangle . \end{aligned}$$
  Here the sign ± is $+$ for out-boundary and − for in-boundary; we also suppressed the $\textrm{out}/\textrm{in}$ subscript in the boundary fields $\textsf{A}^{1,0}$ and *c*.

## BV-BFV Approach to Higher-Dimensional Chern–Simons Theories

The observations on abelian Chern–Simons theory in Sects. 3 and 4 generalize readily to cylinders $I\times M$ of other dimensions *d*. Observe that *d* must be odd because we want the field $\mathcal {A}$ to belong to the superspace $\Omega ^\bullet (I\times M)$ or $\Pi \Omega ^\bullet (I\times M)$ and, in either case, the BV action $S=\int _{ I\times M} \frac{1}{2} \mathcal {A}\wedge d\mathcal {A}$ is even iff *d* is odd.

In the following, we will actually focus on the graded case where the field $\mathcal {A}$ belongs to the graded space $\Omega ^\bullet (I\times M)[k]$ for some integer *k* and the BV action has degree zero. This forces $d=2k+1$. If *k* were even, we would have $\mathcal {A}\wedge d\mathcal {A}=\frac{1}{2}d\mathcal {A}^2$, so the BV action would have no bulk contribution. Therefore, we will have to assume that *k* is odd. To summarize :^56^

$$\begin{aligned} d=\dim (I\times M)=2k+1,\quad k=2l+1. \end{aligned}$$

The case $k=1$ has been considered in Sect. 4. We will briefly describe the general case before turning to the next example of interest, $k=3$.

Next we assume that the 2*k*-dimensional manifold *M* is closed and oriented. Again, we can construct a BV-BFV theory by the AKSZ construction as

$$\begin{aligned} \mathcal {F}= \textrm{Map}(T[1](I \times M), \mathbb {R}[k]) =\Omega ^\bullet (I \times M)[k] \end{aligned}$$

and rewrite this space of fields in the form

$$\begin{aligned} \mathcal {F}= \Omega ^\bullet (I,\Omega ^\bullet (M)[k]), \end{aligned}$$

exhibiting the theory as a 1-dimensional Chern–Simons theory with coefficients in $\mathfrak {g}= \Omega ^\bullet (M)$. The BV action is then, mimicking (18),

$$\begin{aligned} S = \int _{I \times M} \frac{1}{2} \mathcal {A} \wedge d\mathcal {A} = \int _I \frac{1}{2} (\mathcal {A},d_I\mathcal {A}) + \frac{1}{2}(\mathcal {A},d_M\mathcal {A}), \end{aligned}$$

where $d = d_I + d_M$ and $(a,b) = \int _M a \wedge b$. Again, the field $\mathcal {A}$ can be split as $\mathcal {A} = \textsf{A}+ dt \cdot \textsf{A}_I$ and the boundary phase space is $\mathcal {F}^\partial _M = \Omega ^\bullet (M)[k]$ with Noether 1-form

$$\begin{aligned} \alpha =\frac{1}{2} \int _{ \{1\} \times M}\textsf{A}\wedge \delta \textsf{A}- \frac{1}{2} \int _{ \{0\}\times M}\textsf{A}\wedge \delta \textsf{A}. \end{aligned}$$

Next, assume that *M* carries a complex structure. Then we can split the space of complexified *k*-forms as

$$\begin{aligned} \Omega ^k_\mathbb {C}(M) = \bigoplus _{j_1 + j_2 = k}\Omega ^{j_1,j_2}(M). \end{aligned}$$

Given that *k* is odd, the splitting

106 $$\begin{aligned} \Omega ^k_\mathbb {C}(M) = \underbrace{\bigoplus _{j = 0}^{l}\Omega ^{k-j,j}(M)}_{\Omega ^k_+(M)} \oplus \underbrace{\bigoplus _{j = l+1}^{k}\Omega ^{k-j,j}(M)}_{\Omega ^k_-(M)} \end{aligned}$$

provides a splitting into lagrangian subspaces of $\Omega ^k_\mathbb {C}(M)$ (which is the degree 0 subspace of $\mathcal {F}^\partial _M$). The splitting (20) generalizes to

$$\begin{aligned} \underbrace{\Omega ^\bullet _\mathbb {C}(M)}_{\mathfrak {g}_\mathbb {C}} = \underbrace{\bigoplus _{j=0}^{k-1}\Omega ^j_\mathbb {C}(M) \oplus \Omega ^k_+(M)}_{\mathfrak {g}^+_\mathbb {C}} \oplus \underbrace{\Omega ^k_-(M)\oplus \bigoplus _{j=k+1}^{d-1}\Omega _\mathbb {C}^j(M)}_{\mathfrak {g}^-_\mathbb {C}} . \end{aligned}$$

**Fig. 11:**
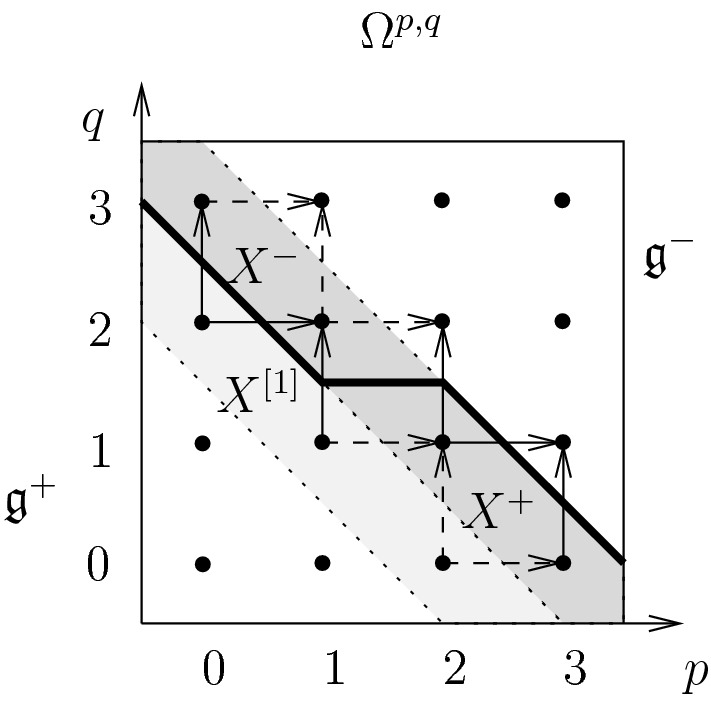
Splitting of $\Omega ^{p,q}$ into $\mathfrak {g}^+$ (below the thick line) and $\mathfrak {g}^-$ (above the thick line), in the case $k=3$. Solid arrows are components of $d^-$, dashed arrows are components of $d^+$. Horizontal arrows (dashed or not) are $\partial$, vertical ones are $\bar{\partial }$

Correspondingly, we split the fields $\textsf{A}$ into its components $\textsf{A}= \textsf{A}^+ + \textsf{A}^-$, and similarly for $\textsf{A}_I$. The de Rham differential restricted to the subcomplex $\Omega ^{k-1}(M)\rightarrow \Omega ^k_+(M) \oplus \Omega ^k_-(M) \rightarrow \Omega ^{k+1}(M)$ splits as $d_M = d_M^+ + d_M^-$ as in Sect. 5.1,^57^ see also Fig. 11.

In particular, we have $(\textsf{A}^+,d_M\textsf{A}^+) = (\textsf{A}^+,d_M^-\textsf{A}^+)$. Before turning to the particular example of $k=3$, let us briefly have a look at the general form of the partition function in the two polarizations considered already in the last section.

### Partition functions in $4l+3$-dimensional CS theories

First, let us take $\mathfrak {g}^+$ as the base of the polarization on both ends of the cylinder. This means that

$$\begin{aligned} \mathcal {B}= \mathfrak {g}^+[k] \oplus \mathfrak {g}^+[k] \ni (\textsf{A}^+_\mathrm{{in}},\textsf{A}^+_\mathrm{{out}}) \end{aligned}$$

with fiber

$$\begin{aligned} \mathcal {Y}= \Omega ^\bullet (I,\partial I; \mathfrak {g}^+[k]) \oplus \Omega ^\bullet (I,\mathfrak {g}^-[k]). \end{aligned}$$

Again, we can gauge fix the polarized theory by choosing

$$\begin{aligned} \mathcal {V} = dt \cdot \mathfrak {g}^+[k] \oplus 1\cdot \mathfrak {g}^-[k] \ni (dt\cdot \textsf{A}^+_{I,\textrm{res}}, \textsf{A}^-_\mathrm{{res}}) \end{aligned}$$

and using Hodge decomposition (7) with chain contraction (8) and corresponding propagator (9). Thus, we obtain the splitting

$$\begin{aligned} \mathcal {A} = \widetilde{\textsf{A}}_\mathrm{{in}}^+ + \widetilde{\textsf{A}}_\mathrm{{out}}^+ + dt\cdot \textsf{A}^+_{I,\textrm{res}} + \textsf{A}^-_\mathrm{{res}} + \textsf{A}^+_\mathrm{{fl}} + \textsf{A}^-_\mathrm{{fl}}, \end{aligned}$$

where $\widetilde{\textsf{A}}_\mathrm{{in}}^+,\widetilde{\textsf{A}}_\mathrm{{out}}^+$ are the discontinuous extensions of $\textsf{A}^+_\mathrm{{in}},\textsf{A}^+_\mathrm{{out}}$ into the bulk. In terms of this splitting, we can rewrite the “perturbation” $\frac{1}{2}\int _{I\times M}\mathcal {A}\wedge d_M\mathcal {A}$ as

$$\begin{aligned} \frac{1}{2} \int _{I\times M}\mathcal {A}\wedge d_M\mathcal {A} = \int _I dt \int _M \textsf{A}^+_{I,\textrm{res}} d_M (\textsf{A}^-_\mathrm{{res}} + \textsf{A}^+_\mathrm{{fl}} + \textsf{A}^-_\mathrm{{fl}}), \end{aligned}$$

since $dt\cdot \textsf{A}^+_{I,\textrm{res}}$ is the only term containing a *dt*. By definition [see (7)] the $\textsf{A}^-_\textrm{fl}$ fluctuations have vanishing integral over *I*. Applying the fact that $d_M = d_M^+ + d_M^-$, we obtain

$$\begin{aligned} \frac{1}{2} \int _{ I \times M }\mathcal {A}\wedge d_M\mathcal {A} = \int _I dt\left( \int _M \textsf{A}^+_{I,\textrm{res}} d_M \textsf{A}^-_\mathrm{{res}} + \int _M \textsf{A}^+_{I,\textrm{res}} d_M^- \textsf{A}^+_\textrm{fl}\right) . \end{aligned}$$

The BV-BFV partition function—as in (22)—is then given by

107 $$\begin{aligned}{} & {} Z(\textsf{A}^+_\mathrm{{in}},\textsf{A}^-_\mathrm{{out}},\textsf{A}^+_{I,\textrm{res}}, \textsf{A}^-_\mathrm{{res}}) \nonumber \\{} & {} \quad = \int _{\mathcal {Y}'_{K-ex}\subset \mathcal {Y}'}\mathcal {D} \textsf{A}^+_\textrm{fl}\;\mathcal {D} \textsf{A}^-_\textrm{fl}\;e^{\frac{i}{\hbar }S^f\left( \widetilde{\textsf{A}^+_\textrm{in}}+\widetilde{\textsf{A}^+_\textrm{out}}+ \textsf{A}^+_\textrm{fl}+\textsf{A}^-_\textrm{res}+\textsf{A}^-_\textrm{fl}+dt\cdot \textsf{A}^+_{I\,\textrm{res}} \right) }\nonumber \\{} & {} \quad =\int \mathcal {D}\textsf{A}^+_\textrm{fl}\;\mathcal {D} \textsf{A}^-_\textrm{fl}\;e^{\frac{i}{\hbar }\big ( \int _{I\times M} \textsf{A}^-_\textrm{fl}d_I\textsf{A}^+_\textrm{fl}+ \int _{\{1\}\times M} \textsf{A}^+_\textrm{out}\textsf{A}^- - \int _{\{0\}\times M} \textsf{A}^+_\textrm{in}\textsf{A}^- + \int _{I\times M} \frac{1}{2}\mathcal {A}\, d_M \mathcal {A}\big )} \nonumber \\{} & {} \quad =\int \mathcal {D} \textsf{A}^+_\textrm{fl}\;\mathcal {D} \textsf{A}^-_\textrm{fl}\; \exp \frac{i}{\hbar } \Big (\int _{I\times M} \textsf{A}^-_\textrm{fl}\, d_I\textsf{A}^+_\textrm{fl}+ \int _{M} \textsf{A}^+_\textrm{out}\, (\textsf{A}^-_\textrm{res}+\textsf{A}^-_\textrm{fl}\big |_{t=1}) \nonumber \\{} & {} \qquad -\, \int _{M} \textsf{A}^+_\textrm{in}\, (\textsf{A}^-_\textrm{res}+\textsf{A}^-_\textrm{fl}\big |_{t=0}) + \int _M \textsf{A}^+_{I\,\textrm{res}} d_M \textsf{A}^-_\textrm{res}+ \int _{I\times M} dt\; \textsf{A}^+_{I\,\textrm{res}} \, d_M^- \textsf{A}^+_\textrm{fl}\Big ).\qquad \end{aligned}$$

This is structurally the same formula as in Sect. 4 before, with the difference that the pairing on residual fields in slightly more complicated in this case. The Feynman diagrams defining this functional integral are the same as in (23) and yield

$$\begin{aligned} Z=\exp \frac{i}{\hbar }\int _M\Big ( (\textsf{A}^+_\textrm{out}- \textsf{A}^+_\textrm{in})\; \textsf{A}^-_\textrm{res}+\textsf{A}^+_{I\,\textrm{res}}\,d_M \textsf{A}^-_\textrm{res}+\frac{1}{2} (\textsf{A}^+_\textrm{out}+\textsf{A}^+_\textrm{in})\;d_M^- \textsf{A}^+_{I\,\textrm{res}} \Big ). \end{aligned}$$

Similarly, the partition function in the holomorphic-to-antiholomorphic polarization, with space of boundary conditions

$$\begin{aligned} \mathcal {B}=\mathfrak {g}^-[k]\oplus \mathfrak {g}^+[k]\ni (\textsf{A}^-_\textrm{in},\textsf{A}^+_\textrm{out}) \end{aligned}$$

and space of residual fields

$$\begin{aligned} \mathcal {V}= dt\cdot \mathfrak {g}_{\mathbb {C}}[k-1]\oplus (1-t)\cdot \mathfrak {g}^+[k] \oplus t\cdot \mathfrak {g}^-[k] \;\; \ni \;\; dt\cdot \textsf{A}_{I\,\textrm{res}}+ (1-t)\cdot \textsf{A}^+_\textrm{res}+ t\cdot \textsf{A}^-_\textrm{res}, \end{aligned}$$

is

$$\begin{aligned}&Z(\textsf{A}^-_\textrm{in},\textsf{A}^+_\textrm{out};\textsf{A}_{I\,\textrm{res}}, \textsf{A}^+_\textrm{res},\textsf{A}^-_\textrm{res}) \\&\quad =\exp \frac{i}{\hbar }\Bigg (\int _M \ -\textsf{A}^+_\textrm{out}\textsf{A}^-_\textrm{in}+\textsf{A}^+_\textrm{out}\textsf{A}^-_\textrm{res}-\textsf{A}^-_\textrm{in}\textsf{A}^+_\textrm{res}+\frac{1}{2} \textsf{A}^-_\textrm{res}\textsf{A}^+_\textrm{res}+\\&\qquad +\,\frac{1}{2} \int _M \textsf{A}_{I\,\textrm{res}}^+ \; (d_M^- \textsf{A}^+_\textrm{res}+d_M\textsf{A}^-_\textrm{res}) + \frac{1}{2}\int _M\textsf{A}_{I\,\textrm{res}}^-d_M\textsf{A}_\textrm{res}^+ \Bigg ).\end{aligned}$$

### Parallel ghost polarization

We can also choose again the “parallel ghost” polarization discussed in Sect. 5.1. To be more explicit, and in preparation for the next section, let us fix $k=3$. Then we have $\mathfrak {g}= \Omega ^\bullet (M,\mathbb {C})$ where *M* is a 6-dimensional manifold with a complex structure, and $X = \mathfrak {g}[3]$.^58^ For later purposes, let us suppose that *M* is endowed with a Kähler metric *g*. We use the complex structure to define the polarization of the $\textrm{gh}= 0$ component of *X*:

$$\begin{aligned} X^{[0]} = \Omega ^3_\mathbb {C}(M) = \underbrace{\Omega ^{3,0}(M) \oplus \Omega ^{2,1}(M)}_{X^+} \oplus \underbrace{\Omega ^{1,2}(M) \oplus \Omega ^{0,3}(M)}_{X^-} . \end{aligned}$$

Correspondingly, we split the field $\textsf{A}= \textsf{A}^{\le 2} + \textsf{A}^{3,+} + \textsf{A}^{3,-} + \textsf{A}^{\ge 4}$ and similarly for $\textsf{A}_I$. We denote by $\mathcal {P}^{[<0],-}$ the polarization given by

$$\begin{aligned} \mathcal {P}^{[<0],-} = \left\{ \frac{\delta }{\delta \textsf{A}^{\ge 4}},\frac{\delta }{\delta \textsf{A}^{3,-}}\right\} , \end{aligned}$$

whose base is parametrized by $(\textsf{A}^{\le 2}, \textsf{A}^{3,+})$, and by $\mathcal {P}^{[<0],+}$ the similar polarization with − and $+$ exchanged. The maps $d_\mathfrak {g}^+,d_\mathfrak {g}^-:X^{[1]} \rightarrow X^{[0]}$ defined in Sect. 5.2 are given by projecting the de Rham differential

$$\begin{aligned} d_M :\Omega ^2_\mathbb {C}(M) \rightarrow \Omega ^3_\mathbb {C}(M) \end{aligned}$$

to $X^\pm$. Explicitly, they are given by

$$\begin{aligned}&d^+_\mathfrak {g}\big |_{\Omega ^{2,0}} = d_M\big |_{\Omega ^{2,0}}, \qquad d^+_\mathfrak {g}\big |_{\Omega ^{1,1}} = \partial _M\big |_{\Omega ^{1,1}},\qquad d^+_\mathfrak {g}\big |_{\Omega ^{0,2}} =0 , \\&d^-_\mathfrak {g}\big |_{\Omega ^{2,0}} =0, \qquad d^-_\mathfrak {g}\big |_{\Omega ^{1,1}} = \bar{\partial }_M\big |_{\Omega ^{1,1}},\qquad d^-_\mathfrak {g}\big |_{\Omega ^{0,2}} =d_M\big |_{\Omega ^{0,2}} , \end{aligned}$$

see Fig. 11. Now, we consider the cylinder $I\times M$ with with $\mathcal {P}^{[<0],+}$ on the in-boundary and $\mathcal {P}^{[<0],-}$ on the out-boundary. We then have the following fields in the effective action (referring to notation from Sect. 5.1):

- $\psi _\textrm{out}^+ = \textsf{A}^{3,+}_\textrm{out}= \textsf{A}^{3,0}_\textrm{out}+ \textsf{A}^{2,1}_\textrm{out}$—physical boundary field on out-boundary,
- $\psi ^-_\textrm{in}=\textsf{A}^{3,-}_\textrm{in}= \textsf{A}^{0,3}_\textrm{in}+ \textsf{A}^{1,2}_\textrm{in}$ —physical boundary field on in-boundary,
- $(\psi ^{[>0]}_\textrm{in},\psi ^{[>0]}_\textrm{out}) = (\textsf{A}_\textrm{in}^{\le 2},\textsf{A}_\textrm{out}^{\le 2})$—boundary fields in higher ghost number (collected in a superfield),
- $A^{[1]}_\textrm{res}= \textsf{A}^2_{I\,\textrm{res}}= \textsf{A}_{I\,\textrm{res}}^{2,0}+\textsf{A}_{I\,\textrm{res}}^{1,1}+\textsf{A}_{I\,\textrm{res}}^{0,2}$—2-form, residual field in ghost number 0,
- $(A^{[>1]}_\textrm{res},\psi _\textrm{res})=(\textsf{A}^{<2}_{I\,\textrm{res}},\textsf{A}_\textrm{res}^{>3})$—residual fields of higher ghost number (form degree $<2$) or negative ghost number (form degree $>3$).

The effective action (46) then reads

$$\begin{aligned} S_\textrm{eff}[\textsf{A}_\textrm{out},\textsf{A}_\textrm{in},\textsf{A}_{I\,\textrm{res}},\textsf{A}_{\textrm{res}}] = S_\textrm{ph}+ S_\textrm{gh}, \end{aligned}$$

where

$$\begin{aligned} S_\textrm{ph}&= \int _M \textsf{A}^{3,0}_\textrm{out}\textsf{A}^{0,3}_\textrm{in}+ \textsf{A}^{2,1}_\textrm{out}\textsf{A}^{1,2}_\textrm{in}+ \textsf{A}^{3,0}_\textrm{out}\bar{\partial }\textsf{A}_{I\,\textrm{res}}^{0,2} + \textsf{A}^{0,3}_\textrm{in}\partial \textsf{A}_{I\,\textrm{res}}^{2,0}\\&\quad +\,\int _M\textsf{A}^{1,2}_\textrm{in}\partial \textsf{A}_{I\,\textrm{res}}^{1,1} +\textsf{A}^{1,2}_\textrm{in}\bar{\partial }\textsf{A}_{I\,\textrm{res}}^{2,0}+\textsf{A}^{2,1}_\textrm{out}\partial \textsf{A}_{I\,\textrm{res}}^{0,2} + \textsf{A}^{2,1}_\textrm{out}\bar{\partial }\textsf{A}_{I\,\textrm{res}}^{1,1} \\&\quad +\,\frac{1}{2}\int _M\bar{\partial }\textsf{A}_{I\,\textrm{res}}^{1,1}\partial \textsf{A}_{I\,\textrm{res}}^{1,1} , \\ S_\textrm{gh}&= \int _M(\textsf{A}^{\le 2}_\textrm{out}- \textsf{A}^{\le 2}_\textrm{in})\textsf{A}^{>3}_\textrm{res}+\textsf{A}_\textrm{res}^{>3}d_M\textsf{A}_{I\,\textrm{res}}^{<2}. \end{aligned}$$

A similar formula holds for higher-dimensional Chern–Simons theories. Some comments:

(i) As a consequence of Propositions 5.4 and 5.3, the effective action satisfies the modified quantum master equation, and the $\textrm{gh}=0$ part $S_\textrm{ph}$ satisfies the generalized Hamilton–Jacobi equations. In particular, $S_\textrm{ph}$ can be identified with the HJ action.
(ii) One can rewrite $S_\textrm{ph}$ as
  $$\begin{aligned} S_\textrm{ph}= \int _M (\textsf{A}^{3,+}_\textrm{out}- d_\mathfrak {g}^+\textsf{A}^2_{I\,\textrm{res}})(A^{3,-}_\textrm{in}+ d_\mathfrak {g}^-\textsf{A}_{I\,\textrm{res}}^2) -\frac{1}{2} \bar{\partial }A^{1,1}_{I\,\textrm{res}}\partial A^{1,1}_{I\,\textrm{res}}\end{aligned}$$
  —a higher-dimensional version of an abelian gauged WZW model, see also footnote 22.

#### Pushforward over residual fields

The space of residual fields is

$$\begin{aligned} \mathcal {V}=\{ dt\cdot \textsf{A}^{\le 2}_{I\,\textrm{res}}+ \textsf{A}_\textrm{res}^{\ge 4}\} \;\; = \Omega ^{\le 2}_\mathbb {C}(M)[2] \oplus \Omega _\mathbb {C}^{\ge 4}(M)[3]. \end{aligned}$$

In particular, the components and their ghost numbers are

$$\begin{aligned} \begin{array}{c|c|c|c|c|c|c} \hbox {field }&{} \textsf{A}^0_{I\,\textrm{res}}&{} \textsf{A}^1_{I\,\textrm{res}}&{} \textsf{A}^2_{I\,\textrm{res}}&{} \textsf{A}^4_\textrm{res}&{} \textsf{A}^5 _\textrm{res}&{} \textsf{A}^6_\textrm{res}\\ \hline \hbox {ghost number} &{} 2 &{} 1 &{} 0 &{} -1 &{} -2 &{} -3 \end{array} \end{aligned}$$

A gauge-fixing lagrangian can be constructed by using the Hodge decomposition for $\bar{\partial }$: Namely, using the Kähler metric *g*, we decompose

$$\begin{aligned} \Omega ^{p,q}(M) = H^{p,q}(M) \oplus \Omega ^{p,q}_{\bar{\partial }-\textrm{ex}} \oplus \Omega ^{p,q}_{\bar{\partial }-\textrm{coex}} \end{aligned}$$

where the middle and rightmost terms denote the spaces of $\bar{\partial }$-exact and $\bar{\partial }^*$-exact forms, respectively. The gauge-fixing lagrangian $\mathcal {L}\subset \mathcal {V}$ is then defined as

108 $$\begin{aligned} \mathcal {L}=\bigoplus _{p+q \le 2} \Omega ^{p,q}_{\bar{\partial }-\textrm{coex}} \oplus \bigoplus _{p+q \ge 4}\left( H^{p,q}(M) \oplus \Omega ^{p,q}_{\bar{\partial }-\textrm{coex}} \right) . \end{aligned}$$

Restricted to this gauge-fixing lagrangian, the effective action is nondegenerate in residual fields and the integral gives

$$\begin{aligned}{} & {} Z_*\propto \delta (\bar{\partial }\textsf{A}^{3,0}_\textrm{out}+\partial \textsf{A}^{2,1}_{\textrm{out},\bar{\partial }\mathrm {-ex}}) \, \delta (\bar{\partial }\textsf{A}^{1,2}_\textrm{in}+\partial \textsf{A}^{0,3}_{\textrm{in},\bar{\partial }\mathrm {-ex}}) \, \delta (\textsf{A}^{\le 2}_{\textrm{out},\textrm{harm}}-\textsf{A}^{\le 2}_{\textrm{in},\textrm{harm}})\cdot \\{} & {} \quad \cdot \exp \frac{i}{\hbar }\bigg ( \int _M \big (\textsf{A}^{3,0}_\textrm{out}\textsf{A}^{0,3}_\textrm{in}+ \textsf{A}^{2,1}_\textrm{out}(\textrm{id}-P_{\bar{\partial }\mathrm {-ex}}) \textsf{A}^{1,2}_\textrm{in}\big )-\\{} & {} \quad -\frac{1}{2} \int _{M\times M}\bar{\partial }\textsf{A}^{2,1}_\textrm{out}(x)K(x,x')\bar{\partial }\textsf{A}^{2,1}_\textrm{out}(x') -\frac{1}{2} \int _{M\times M}\partial \textsf{A}^{1,2}_\textrm{in}(x)K(x,x')\partial \textsf{A}^{1,2}_\textrm{in}(x') \bigg ), \end{aligned}$$

where $K(x,x')$ is the integral kernel of the inverse of the operator $(\partial \bar{\partial })$ restricted to $\Omega ^{1,1}_{\bar{\partial }-\textrm{coex}}$.

### 7D Chern–Simons and Kodaira–Spencer action functional

We now turn our attention to 7-dimensional Chern–Simons theory on a cylinder with a particular polarization on the out-boundary. This polarization was first discovered by Hitchin [27]. It was used in [24] to argue that the semi-classical approximation of the Chern–Simons wave function can be expressed in terms of the Kodaira–Spencer action functional introduced in [8] whose classical solutions are deformations of complex structures on a Kähler manifold (see Appendix B for a brief review of the Kodaira-Spencer theory). Here we argue that this semiclassical approximation is in fact exact in the axial gauge. From the general arguments of the BV-BFV formalism, it follows that a change of gauge fixing will result in an $\Omega$-exact change of the partition function, hence its $\Omega$-cohomology class is well-defined and given by the Kodaira–Spencer partition function. A caveat is that in this section we do not take care of determinants arising in Gaussian path integrals. Those might lead to anomalies similar to the discussion of Remark 4.4, and would have to be treated separately.

#### General polarizations in $4l+3$-dimensional Chern–Simons theory

Using the results of Sect. 5.2, one can consider also more general polarizations in higher-dimensional Chern–Simons theories, in dimension $d = 2k+1 = 4l+3$.

Suppose that $\mathcal {P}^{[0]}$ is any polarization on $X^{[0]} = \Omega ^k_\mathbb {C}(M)$ such that we have local coordinates $\textsf{A}^Q$ on the base and $\textsf{A}^P$ on the fibers, and let $G = G(\textsf{A}^{-},\textsf{A}^Q)$ be the corresponding generating function. From Sect. 5.2 we know that the partition function of abelian Chern–Simons theory with $\mathcal {P}^{[<0],+}$-polarization on the in-boundary and $\mathcal {P}^{[<0],P}$-polarization on the out-boundary is $Z = \exp (\frac{i}{\hbar } S_\textrm{eff})$ with $S_\textrm{eff}= S_\textrm{ph}+ S_\textrm{gh}$ and

109 $$\begin{aligned} S_\textrm{ph}[\textsf{A}^-_\textrm{in},\textsf{A}^Q_\textrm{out},\textsf{A}_{I\,\textrm{res}}]&= \frac{1}{2}\int _M \partial _M \textsf{A}^{l,l}_{I\,\textrm{res}}\bar{\partial }_M \textsf{A}^{l,l}_{I\,\textrm{res}}+ \int _M \textsf{A}^{k,-}_\textrm{in}d_\mathfrak {g}^+\textsf{A}^{k-1}_{I\,\textrm{res}}\nonumber \\&\quad - \,G(\textsf{A}^{k,-}_\textrm{in}+ d_\mathfrak {g}^-\textsf{A}^{k-1}_{I\,\textrm{res}},\textsf{A}^Q_\textrm{out}) , \nonumber \\ S_\textrm{gh}[\textsf{A}^{[>0]}_\textrm{in},\textsf{A}^{[>0]}_\textrm{out},\textsf{A}_{I\,\textrm{res}},\textsf{A}_\textrm{res}]&= \int _M(\textsf{A}^{[>0]}_\textrm{out}- \textsf{A}^{[>0]}_\textrm{in})\textsf{A}^{[<0]}_\textrm{res}+ \int _M\textsf{A}_\textrm{res}^{[<0]}d_M\textsf{A}_{I\,\textrm{res}}^{[>0]}. \end{aligned}$$

See also the toy model in considered [14, Section 12].

#### Hitchin polarization on 6-dimensional manifolds and effective action

In 7-dimensional Chern–Simons theory, there is an interesting—nonlinear—polarization on the boundary phase space, coming from the special geometry of three-forms in six dimensions first described by Hitchin in [27]. The idea is as follows. A complex 3-form *A* on a six-dimensional manifold with a complex structure decomposes as

110 $$\begin{aligned} A = A^{+,\textrm{nl}} + A^{-,\textrm{nl}} \end{aligned}$$

where $A^{+,\textrm{nl}}$ and $A^{-,\textrm{nl}}$ are decomposable complex 3-forms, i.e., triple wedge products:

$$\begin{aligned} A^{\pm ,\textrm{nl}} = \theta _1^\pm \wedge \theta _2^\pm \wedge \theta _3^\pm , \qquad \theta _i^\pm \in \Omega ^1(M,\mathbb {C}). \end{aligned}$$

The 3-form *A* is called *nondegenerate* if $A^{+,\textrm{nl}} \wedge A^{-,\textrm{nl}}$ is everywhere nonvanishing (which is equivalent to the fact that the form *A* is not decomposable). In this case $A^{+,\textrm{nl}}$ and $A^{-,\textrm{nl}}$ are uniquely determined by *A* and define a polarization of $\Omega ^3(M)_{\textrm{nd}}$, the subset of nondegenerate forms. For more details on this polarization, we refer to Gerasimov and Shatashvili [24]. The effective action on the cylinder with $\mathcal {P}^{[<0],-,\textrm{l}}$-polarization on the in-boundary^59^ and $\mathcal {P}^{[<0],+,\textrm{nl}}$-polarization on the out-boundary thus reads $S_\textrm{eff}= S_\textrm{ph}+ S_\textrm{gh}$ with $S_\textrm{gh}$ given by (109) and111

##### Proposition 6.1

The partition function is given by

$$\begin{aligned} Z = \exp \frac{i}{\hbar }\left( S_{\textrm{ph}} + S_{\textrm{gh}}\right) , \end{aligned}$$

with $S_\textrm{ph}$ given by (111) and $S_\textrm{gh}$ given by (109). It satisfies the modified quantum master equation

$$\begin{aligned} ( \Omega _\textrm{in}+ \Omega _\textrm{out}- \hbar ^2\Delta _\textrm{res})Z = 0, \end{aligned}$$

with $\Omega _\textrm{in}$, $\Omega _\textrm{out}$ given by the standard quantization of the BFV boundary action $\frac{1}{2}\int _M \textsf{A}d\textsf{A}$:

$$\begin{aligned} \Omega _\textrm{in}&= \int _M d\textsf{A}_\textrm{in}^{2}\left( \textsf{A}^{+,\textrm{l}}_\textrm{in}- i\hbar \frac{\delta }{\delta \textsf{A}_\textrm{in}^{+,\textrm{l}}}\right) -i\hbar \int _M d\textsf{A}_\textrm{in}^{1}\frac{\delta }{\delta \textsf{A}_\textrm{in}^{4}}-i\hbar \int _M d\textsf{A}_\textrm{in}^{0}\frac{\delta }{\delta \textsf{A}_\textrm{in}^{5}}, \\ \Omega _\textrm{out}&= \int _M d\textsf{A}_\textrm{out}^{2}\left( \textsf{A}^{-,\textrm{nl}}_\textrm{out}- i\hbar \frac{\delta }{\delta \textsf{A}^{-,\textrm{nl}}_\textrm{out}}\right) -i\hbar \int _M d\textsf{A}_\textrm{out}^{1}\frac{\delta }{\delta \textsf{A}_\textrm{out}^{4}}-i\hbar \int _M d\textsf{A}_\textrm{out}^{0}\frac{\delta }{\delta \textsf{A}_\textrm{out}^{5}}. \end{aligned}$$

##### Proof

We are in the situation of Remark 5.8 here because of the fact that the boundary action is linear in the canonical variables defining the Hitchin polarization, see equation (110). It follows that the mQME is satisfied for the standard quantization of the boundary action. $\square$

##### Remark 6.2

We want to stress that in this gauge, the semiclassical approximation to this partition function that was used as an ansatz in [24] (the integral kernel of the generalized Segal–Bargmann transform^60^ from the linear to the nonlinear polarization) is found to be *exact*: there are no quantum corrections. Adapting the proof of [14, Appendix B] to the infinite-dimensional setting one can show that changing the gauge fixing by changing the propagator on the interval results in a change of the partition function by a $(\hbar ^2\Delta _\textrm{res}- \Omega )$-cocycle.

##### Remark 6.3

To be precise in comparison with Gerasimov and Shatashvili [24], one should identify the residual fields in (111) with the Lagrange multipliers enforcing the constraint $d_M\textsf{A}=0$ in [24]. This, of course, is precisely their role when interpreting (111) as the Hamilton–Jacobi action for 7D Chern–Simons theory in the chosen polarizations.

#### Comparison with Kodaira–Spencer gravity

Following Gerasimov and Shatashvili [24], we want to compare the Chern–Simons effective action (111) with the Kodaira–Spencer action functional (145). Let us fix a reference holomorphic 3-form $\omega _0\in \Omega ^{3,0}(M)$. Its conjugate is an antiholomorphic 3-form $\overline{\omega }_0 \in \Omega ^{0,3}(M)$. We can then parametrize $A^{+,\textrm{nl}}$ and $A^{-,\textrm{nl}}$ as

$$\begin{aligned} A^{+,\textrm{nl}}&= \rho e^\mu \omega _0, \\ A^{-,\textrm{nl}}&= \overline{\rho } e^{\overline{\mu }}\overline{\omega }_0, \end{aligned}$$

where $$\rho ,\overline{\rho } \in \Omega ^0_\mathbb {C}(M)$$, $$\mu \in \Omega ^{-1,1}(M)$$, $$\overline{\mu }\in \Omega ^{1,-1}(M)$$ and

112 $$\begin{aligned} \rho e^\mu \omega _0 = \rho \left( \omega _0 + \mu \omega _0 + \frac{\mu ^2}{2}\omega _0 + \frac{\mu ^3}{6}\omega _0 \right) , \end{aligned}$$

where $\mu \omega _0$ should be interpreted as extension of contraction to forms with values in vector fields.

Of course, a complex 3-form still has a decomposition $A = A^{+,\textrm{l}} + A^{-,\textrm{l}}$, with $A^{+,\textrm{l}} \in \Omega ^{3,0}(M) \oplus \Omega ^{2,1}(M)$, and $A^{-,\textrm{l}} \in \Omega ^{1,2}(M) \oplus \Omega ^{0,3}(M)$.

The following expression for *G* is given in [24]:

113 $$\begin{aligned}{} & {} G(A^{3,0},A^{2,1},\overline{\rho },\overline{\mu }) \nonumber \\{} & {} \quad =\int _M \overline{\rho }( A^{3,0}\overline{\omega }_0 + A^{2,1}\overline{\mu }\,\overline{\omega }_0) + \overline{\rho }^2\langle \overline{\mu }^3\rangle \omega _0\overline{\omega }_0 - \frac{\left\langle \left( (A^{2,1} - \frac{1}{2}\overline{\rho }\,\overline{\mu }^2\overline{\omega }_0)^\vee \right) ^3\right\rangle }{(A^{3,0})^\vee - \overline{\rho }\langle \overline{\mu }^3\rangle }\omega _0\overline{\omega }_0 . \nonumber \\ \end{aligned}$$

For completeness, we present a derivation of this formula in Appendix B.2. Here for $\overline{\mu } \in \Omega ^{1,-1}(M)$ the function $\langle \mu ^3 \rangle$ is defined in (127) and for any $A \in \Omega ^{p,q}$ we define $A^\vee \in \Omega ^{p-3,q}$ by $A^\vee \omega _0 =A$.

Consider now the state

114 $$\begin{aligned} \psi \left( \overline{\rho }_\textrm{out},\overline{\mu }_\textrm{out},\overline{\textsf{A}^{[>0]}_\textrm{out}}\right) = \delta (\overline{\mu }_\textrm{out})\delta \left( \textsf{A}^{[>0]}_\textrm{out}\right) \exp \frac{i}{\hbar }\int _M\overline{\rho }_\textrm{out}\omega _0\overline{\omega }_0. \end{aligned}$$

This is an extension of the physical state^61^

$$\begin{aligned} \psi _\textrm{ph}(\overline{\mu },\overline{\rho }) = \delta (\overline{\mu }_\textrm{out})\exp \frac{i}{\hbar }\int _M\overline{\rho }_\textrm{out}\omega _0\overline{\omega }_0 \end{aligned}$$

to the ghost sector. Note that this state trivially satisfies the mQME because $\Omega _\textrm{out}\psi _\textrm{ph}$ is linear in the ghost fields, but we are multiplying with the ghost delta function.^62^ Now we compute (formally) the vector $Z\left| \psi \right\rangle$ (put differently, we are specifying a boundary condition on the $\textrm{out}$-boundary for the quantum theory). This means computing the following functional integral:

$$\begin{aligned} Z \left| \psi \right\rangle&= \int \mathcal {D}\overline{\rho }_\textrm{out}\mathcal {D}\overline{\mu }_\textrm{out}\mathcal {D}\textsf{A}_\textrm{out}^{[>0]} Z \cdot \psi \\&= \int \mathcal {D}\overline{\rho }_\textrm{out}Z[\textsf{A}^{+,\textrm{l}}_\textrm{in},\textsf{A}^{[>0]}_\textrm{in},\overline{\rho }_\textrm{out},0,0,\textsf{A}^{\ge 4}_\textrm{res}, \textsf{A}^{\le 2}_{I\,\textrm{res}}]\exp \frac{i}{\hbar }\int _M\overline{\rho }_\textrm{out}\omega _0\overline{\omega }_0 \\&=: Z'[\textsf{A}^{+,\textrm{l}}_\textrm{in},\textsf{A}^{[>0]}_\textrm{in},\textsf{A}^{\ge 4}_\textrm{res}, \textsf{A}^{\le 2}_{I\,\textrm{res}}] . \end{aligned}$$

The partition function *Z* depends on $\overline{\rho }_\textrm{out}$ only through *G* and we have

115 $$\begin{aligned}{} & {} \int \mathcal {D}\overline{\rho }\;e^{-\frac{i}{\hbar }G(A^{3,0},A^{2,1},\overline{\rho },0)+\frac{i}{\hbar }\int _M\overline{\rho }\omega _0\overline{\omega }_0} \nonumber \\{} & {} \quad =\delta (A^{3,0}-\omega _0)\, \exp \left( -\frac{i}{\hbar } \int _M \frac{1}{6} \langle A^{2,1}, A^{2,1},A^{2,1}\rangle \right) . \end{aligned}$$

Here for $A \in \Omega ^{2,1}(M)$ we have $\frac{1}{6}\langle A,A,A\rangle := \langle (A^\vee )^3\rangle \omega _0\overline{\omega }_0$. Thus, $Z'$ has the following expression:

116 $$\begin{aligned} Z'&= Z'_\textrm{ph}Z'_\textrm{gh}, \quad \text{ where } \nonumber \\ Z'_\textrm{ph}&= \delta (\textsf{A}^{3,0}_\textrm{in}+ \partial \textsf{A}^{2,0}_{I\,\textrm{res}}- \omega _0) \, \exp \frac{i}{\hbar } \bigg (\frac{1}{2} \int _M \partial \textsf{A}^{1,1}_{I\,\textrm{res}}\bar{\partial }\textsf{A}^{1,1}_{I\,\textrm{res}}+ \int _M \textsf{A}^{3,0}_\textrm{in}\bar{\partial }\textsf{A}^{0,2}_{I\,\textrm{res}} \end{aligned}$$

117 $$\begin{aligned}&\quad +\,\int _M \textsf{A}^{2,1}_\textrm{in}\partial \textsf{A}^{0,2}_{I\,\textrm{res}}+ \textsf{A}^{2,1}_\textrm{in}\bar{\partial }\textsf{A}^{1,1}_{I\,\textrm{res}}- \langle ( (\textsf{A}_\textrm{in}^{2,1}+ \bar{\partial }\textsf{A}^{2,0}_{I\,\textrm{res}}+ \partial \textsf{A}^{1,1}_{I\,\textrm{res}})^\vee )^3\rangle \omega _0\overline{\omega }_0\bigg ),\nonumber \\ Z'_\textrm{gh}&= \exp \frac{i}{\hbar }\left( \int _M -\textsf{A}^{\le 2}_\textrm{in}\textsf{A}^{\ge 4}_\textrm{res}+ \int _M \textsf{A}^{\ge 4}_\textrm{res}d_M\textsf{A}^{\le 1}_{I\,\textrm{res}}\right) . \end{aligned}$$

We stress that $Z'$ was obtained from *Z* through a formal functional integral. However, we have the following result.

##### Lemma 6.4

The function $Z'[\textsf{A}^{+,\textrm{l}}_\textrm{in},\textsf{A}^{[>0]}_\textrm{in},\textsf{A}^{\ge 4}_\textrm{res},\textsf{A}^{\le 2}_{I\,\textrm{res}}]$ satisfies the modified quantum master equation, i.e., is an $(\Omega _\textrm{in}- \hbar ^2\Delta _\textrm{res})$-cocycle, where $\Omega _\textrm{in}= \Omega ^{(0)}_\textrm{in}+ \Omega ^{(1)}_\textrm{in}$ is the standard quantization of $-\frac{1}{2}\int _M \textsf{A}d_M \textsf{A}$ in the $\mathcal {P}^{[<0],-}$-polarization, explicitly given by

$$\begin{aligned} \Omega ^{(0)}_\textrm{in}&= -\int _M \textsf{A}^{2,1}_\textrm{in}\bar{\partial }\textsf{A}^{1,1}_\textrm{in}+ \textsf{A}^{2,1}_\textrm{in}\partial \textsf{A}_\textrm{in}^{0,2} + \textsf{A}_\textrm{in}^{3,0}\bar{\partial }\textsf{A}_\textrm{in}^{0,2}, \\ \frac{i}{\hbar }\Omega ^{(1)}_\textrm{in}&= -\int _M \frac{\delta }{\delta \textsf{A}^{3,0}_\textrm{in}}\partial \textsf{A}^{2,0}_\textrm{in}+ \frac{\delta }{\delta \textsf{A}^{2,1}_\textrm{in}}\bar{\partial }\textsf{A}^{2,0}_\textrm{in}+ \frac{\delta }{\delta \textsf{A}^{2,1}_\textrm{in}}\partial \textsf{A}^{1,1}_\textrm{in}+ \frac{\delta }{\delta \textsf{A}^{\le 2}_\textrm{in}}d\textsf{A}^{\le 1}_\textrm{in}. \end{aligned}$$

This is expected because $\psi$ satisfies $\Omega _\textrm{out}\psi = 0$ and *Z* satisfies the mQME. See also the discussions of gluing in [14, Sections 11.4,12.2]. The interpretation of this Lemma is that $Z'$ is a valid state in the linear polarization.

##### Proof

We will only prove the claim in ghost number 0, since in positive ghost number the effective action is the same as for linear polarizations. To begin, we note that any function of $\textsf{A}^{3,0}_\textrm{in}+ \partial \textsf{A}^{2,0}_{I\,\textrm{res}}$ or $\textsf{A}_\textrm{in}^{2,1} + \bar{\partial }\textsf{A}^{2,0}_{I\,\textrm{res}}+ \partial \textsf{A}^{1,1}_{I\,\textrm{res}}$ is $( i \hbar \{S_\textrm{gh},\bullet \}_\textrm{res}- \Omega ^{(1)}_\textrm{in})$-closed since $\{S_\textrm{gh},\bullet \}_\textrm{res}\big |_{\textrm{gh}=0} = \int _M\textsf{A}^{i,j}_\textrm{in}\frac{\delta }{\delta \textsf{A}^{i,j}_{I\,\textrm{res}}}$. This implies that the delta function in (116) and the last term in (117) are $\hbar ^2\Delta _\textrm{res}- \Omega ^{(1)}$ closed. It is a straightforward check that the remaining exponential terms are $(\hbar ^2\Delta _\textrm{res}- \Omega ^{(0)} - \Omega ^{(1)})$-closed, which concludes the proof. $\square$

We will now argue that formally integrating out the residual fields, in ghost number 0 we obtain the Kodaira–Spencer action. Let us restrict to the gauge-fixing lagrangian $\mathcal {L}$ defined similarly to (108), but given in ghost number 0 by $\partial ^*$-exact 2-forms. We will denote

$$\begin{aligned} Z''[\textsf{A}^{3,0}_\textrm{in},\textsf{A}^{2,1}_\textrm{in},\textsf{A}^{[>0]}_\textrm{in}] = \int _\mathcal {L}Z'[\textsf{A}^{+,\textrm{l}}_\textrm{in},\textsf{A}^{[>0]}_\textrm{in},\textsf{A}^{\ge 4}_\textrm{res},\textsf{A}^{\le 2}_{I\,\textrm{res}}] . \end{aligned}$$

The modified quantum master equation implies that for a (2, 0)-form $\chi$ one has

$$\begin{aligned} Z'[\textsf{A}^{+,\textrm{l}}_\textrm{in}+d \chi ,\textsf{A}^{[>0]}_\textrm{in},\textsf{A}^{\ge 4}_\textrm{res},\textsf{A}^{\le 2}_{I\,\textrm{res}}] = Z'[\textsf{A}^{+,\textrm{l}}_\textrm{in},\textsf{A}^{[>0]}_\textrm{in},\textsf{A}^{\ge 4}_\textrm{res},\textsf{A}^{\le 2}_{I\,\textrm{res}}- \chi ]. \end{aligned}$$

By a change of variables, this implies

$$\begin{aligned} Z''[\textsf{A}^{3,0}_\textrm{in}+\partial \chi ,\textsf{A}^{2,1}_\textrm{in}+\bar{\partial }\chi ,\textsf{A}^{[>0]}_\textrm{in}] = Z''[\textsf{A}^{3,0}_\textrm{in},\textsf{A}^{2,1}_\textrm{in},\textsf{A}^{[>0]}_\textrm{in}] . \end{aligned}$$

We can use this property to reduce the computation of $Z''$ to the case $\textsf{A}^{3,0} = \rho _0\omega _0$, where $\rho _0$ is a constant. The $\delta$ function in $Z'_\textrm{ph}$ then factorizes as $\delta (\rho _0 - 1)\delta ( \partial A^{2,0}_{I\,\textrm{res}})$. Since $\partial$ is an isomorphism on the gauge-fixing lagrangian, the integral over $\textsf{A}^{2,0}_{I\,\textrm{res}}$ gives

$$\begin{aligned} Z''_\textrm{ph}= & {} \int \mathcal {D}A^{0,2}_{I\,\textrm{res}}A^{1,1}_{I\,\textrm{res}}\delta (\rho _0 -1) \exp \frac{i}{\hbar } \bigg (-\frac{1}{2} \int _M \partial \textsf{A}^{1,1}_{I\,\textrm{res}}\bar{\partial }\textsf{A}^{1,1}_{I\,\textrm{res}}+\\{} & {} +\,\int _M \textsf{A}^{2,1}_\textrm{in}\partial \textsf{A}^{0,2}_{I\,\textrm{res}}+ \textsf{A}^{2,1}_\textrm{in}\bar{\partial }\textsf{A}^{1,1}_{I\,\textrm{res}}- \langle ( (\textsf{A}_\textrm{in}^{2,1} + \partial \textsf{A}^{1,1}_{I\,\textrm{res}})^\vee )^3\rangle \omega _0\overline{\omega }_0\bigg ) \end{aligned}$$

and the integral over $A^{0,2}_{I\,\textrm{res}}$ then gives

$$\begin{aligned} Z''_\textrm{ph}= & {} \int \mathcal {D}\textsf{A}^{1,1}_{I\,\textrm{res}}\delta (\rho _0 -1)\delta (\partial \textsf{A}_\textrm{in}^{2,1})\cdot \\{} & {} \cdot \exp \frac{i}{\hbar } \bigg ( \int _M \frac{1}{2}\partial \textsf{A}^{1,1}_{I\,\textrm{res}}\bar{\partial }\textsf{A}^{1,1}_{I\,\textrm{res}}+ \textsf{A}^{2,1}_\textrm{in}\bar{\partial }\textsf{A}^{1,1}_{I\,\textrm{res}}-\langle ( (\textsf{A}_\textrm{in}^{2,1} + \partial \textsf{A}^{1,1}_{I\,\textrm{res}})^\vee )^3\rangle \omega _0\overline{\omega }_0\bigg ). \end{aligned}$$

Finally, writing $\textsf{A}^{2,1}_\textrm{in}$ in the Hodge decomposition $\textsf{A}^{2,1}_\textrm{in}= x + \partial \lambda + \partial ^*\tau$, we obtain by another change of variables^63^$b = \textsf{A}^{1,1}_{I\,\textrm{res}}+ \lambda$ the expression

118 $$\begin{aligned} Z''_\textrm{ph}[\rho _0,x,\lambda ,\tau ]= & {} \delta (\rho _0 -1)\delta (\partial \partial ^*\tau )\cdot \int _{\mathcal {L}\cap \Omega ^{1,1}(M)}\mathcal {D}b\;\exp \frac{i}{\hbar }\int _M\nonumber \\{} & {} \times \left( -\frac{1}{2}\partial \lambda \bar{\partial }\lambda +\frac{1}{2}\partial b \bar{\partial }b+ \bar{\partial }b \partial \lambda + \frac{1}{6}\langle (x+\partial b), (x + \partial b), (x + \partial b) \rangle \right) , \nonumber \\ \end{aligned}$$

which coincides with eq. (2.50) in [24]. Thus, we see that the Chern–Simons partition function on a cylinder, paired with the state (114), coincides with the partition function of Kodaira–Spencer theory with background *x* and action functional given in (145) for $\lambda = 0$. The latter integral can be evaluated perturbatively in terms of Feynman graphs and rules. It would be interesting to compare our results to other constructions of the BCOV theory, such as in [15].

##### Remark 6.5

(*On gauge invariance of* $Z''$) If one uses formally the properties of the BV integral, it is immediate that the $Z''$ gives a class in $\Omega _\textrm{in}$-cohomology independent of the gauge-fixing lagrangian $\mathcal {L}$.^64^ We see here that this cohomology class has a representative given in terms of the KS partition function. The partition function $Z''$ can be also interpreted as the BV-BFV partition function on the cylinder paired with the state $\psi$ at the $\textrm{out}$-boundary, with *all* fields integrated out using an axial-type gauge (the components of the gauge field involving *dt* are set to zero). Another open question is how $Z''$ behaves when we deform away from this type of gauge to a general gauge fixing on the cylinder (say, one given by a Riemannian metric). This is a subject of ongoing research.

## Segal–Bargmann Transform via BV-BFV

Recall (see [26] for details) that the Segal–Bargmann space $\mathcal {H}^\textrm{SB}$ is the Hilbert space of holomorphic functions $\psi (z)$ on $\mathbb {C}$ satisfying

$$\begin{aligned} \int _\mathbb {C}\frac{i}{4\pi \hbar } dz\,d\bar{z} \; e^{-\frac{|z|^2}{2\hbar }} \;\overline{\psi (z)}\psi (z)<\infty \end{aligned}$$

(here we assume that $\hbar$ is a fixed positive number), equipped with inner product

119 $$\begin{aligned} \langle \psi _1,\psi _2 \rangle =\int _\mathbb {C}\frac{i}{4\pi \hbar } dz\,d\bar{z} \; e^{-\frac{|z|^2}{2\hbar }} \;\overline{\psi _1(z)}\psi _2(z). \end{aligned}$$

The Segal–Bargmann space is isomorphic to the Hilbert space $L^2(\mathbb {R})$ of square-integrable functions on $\mathbb {R}$, with the unitary isomorphism $L^2(\mathbb {R})\rightarrow \mathcal {H}^\textrm{SB}$ given by the Segal–Bargmann transform:

120 $$\begin{aligned} \chi (x)\mapsto \psi (z)=(\pi \hbar )^{-\frac{1}{4}}\int _\mathbb {R}dx\; e^{-\frac{1}{\hbar }\left( \frac{z^2}{4}-zx+\frac{x^2}{2}\right) }\; \chi (x). \end{aligned}$$

Now we would like to show how the transformation (120) can be seen as the partition function for topological quantum mechanics on an interval with appropriate boundary polarizations.

Consider topological quantum mechanics on the interval *I* parametrized by $0\le t\le 1$—the theory with 0-form fields $x,p\in \Omega ^0(I)$ and action

121 $$\begin{aligned} S=\int _I p\, d x . \end{aligned}$$

In the BV-BFV formalism, we adjoin the anti-fields $x^*,p^*\in \Omega ^1(I)$—1-form fields carrying ghost number $-1$ (while *x*, *p* carry ghost number 0), so that the odd symplectic form on BV fields is: $\int _I \delta x\wedge \delta x^* + \delta p\wedge \delta p^*$. The BFV phase space assigned to a point $\textrm{pt}^\pm$ (where ± is the orientation) is: $\mathcal {F}^\partial =\mathbb {R}^2\; \ni (x,p)$ with the Noether 1-form $\alpha _{\textrm{pt}^\pm }=\pm p\,\delta x$ and vanishing BFV action $S_\textrm{pt}=0$.

Alongside the real coordinates *x*, *p* on the phase space, we will consider the complex coordinates $z=x-ip$, $\bar{z}=x+ip$. The symplectic structure on the phase space is $\omega _{\textrm{pt}^\pm }=\delta \alpha _{\textrm{pt}^\pm }={\pm }\delta p\wedge \delta x$. Written in complex coordinates it has the form ${\mp }\frac{i}{2}\delta \bar{z}\wedge \delta z$.

Consider the polarization $\textrm{Span}\{\frac{\partial }{\partial p}\}$ (i.e., *x* fixed) at $t=0$ and the polarization $\textrm{Span}\{\frac{\partial }{\partial \bar{z}}\}$ (i.e., *z* fixed) at $t=1$. The corresponding modification of the action (121) by a boundary term is:

122 $$\begin{aligned} S^f=S\underbrace{-\big (\frac{i}{4}x^2+\frac{1}{2} xp +\frac{i}{4} p^2 \big )}_{f}\Big |_{t=1} \end{aligned}$$

—this boundary term is chosen so that one has $-\frac{i}{2}\bar{z}\delta z= p\delta x+\delta f$. Thus, the corresponding boundary Noether 1-form is:

123 $$\begin{aligned} \alpha _{\partial I}^f=-\frac{i}{2}\bar{z}\,\delta z\big |_{t=1} - p\,\delta x \big |_{t=0} \end{aligned}$$

—it vanishes along the chosen polarization, as desired.

Next consider the following splitting of the (complexified) phase space

$$\begin{aligned} \mathcal {F}^\partial _\mathbb {C}=\mathfrak {g}^+\oplus \mathfrak {g}^- , \end{aligned}$$

where $\mathfrak {g}^+$ is parametrized by *z* and $\mathfrak {g}^-$ is parametrized by *x* (we are borrowing the notations from (5) here). The space of fields $\mathcal {F}=\Omega ^\bullet (I,\mathfrak {g}^+)\oplus \Omega ^\bullet (I,\mathfrak {g}^-)$ is fibered over $\mathcal {B}\ni (z_\textrm{out},x_\textrm{in})$ with the fiber

$$\begin{aligned} \mathcal {Y}= & {} \Omega ^\bullet (I,\{1\};\mathfrak {g}^+)\oplus \Omega ^\bullet (I,\{0\};\mathfrak {g}^-)\\= & {} \underbrace{\big (\Omega ^0(I,\{1\};\mathfrak {g}^+)\oplus \Omega ^0(I,\{0\};\mathfrak {g}^-)\big )}_{\mathcal {Y}'_{K-ex}}\bigoplus \underbrace{\big (\Omega ^1(I;\mathfrak {g}^+[-1])\oplus \Omega ^1(I;\mathfrak {g}^-[-1])\big )}_{\mathcal {Y}'_{d-ex}} . \end{aligned}$$

This is an acyclic complex, and thus we can choose the space of residual fields to be zero (cf. Remark 3.1). The corresponding propagator—the integral kernel of the chain contraction *K*—is:

$$\begin{aligned} \eta (t,t')= -\pi ^+\otimes \theta (t'-t) + \pi ^-\otimes \theta (t-t') . \end{aligned}$$

The BV-BFV partition function is then given by the following path integral

124 $$\begin{aligned} Z(z_\textrm{out},x_\textrm{in})= & {} \int \mathcal {D}z_\textrm{fl}\;\mathcal {D}x_\textrm{fl}\; e^{\frac{i}{\hbar }S^f(\widetilde{z_\textrm{out}}+z_\textrm{fl},\widetilde{x_\textrm{in}}+x_\textrm{fl})}\nonumber \\= & {} \int \mathcal {D}z_\textrm{fl}\;\mathcal {D}x_\textrm{fl}\;e^{\frac{i}{\hbar } \Big (i\int _I z_\textrm{fl}\,dx_\textrm{fl}+\big (-ix_\textrm{fl}(1)z_\textrm{out}+\frac{i}{4}z^2_\textrm{out}\big ) +\big (-iz_\textrm{fl}(0)\,x_\textrm{in}+\frac{i}{2}x_\textrm{in}^2\big ) \Big ) }\nonumber \\= & {} e^{-\frac{1}{\hbar }\big (\frac{z^2_\textrm{out}}{4}-z_\textrm{out}x_\textrm{in}+\frac{x^2_\textrm{in}}{2}\big )} . \end{aligned}$$

Here we have a contribution from the Feynman diagram with single propagator connecting $z_\textrm{out}$ and $x_\textrm{in}$. In (124) we recognize the integral kernel of the Segal–Bargmann transform (120). This is, of course, to be expected: the partition function for a cylinder (in this case, an interval), with polarization $\mathcal {P}_1$ on the in-boundary and polarization $\mathcal {P}_2$ on the out-boundary, maps $\mathcal {P}_1$-states to $\mathcal {P}_2$-states.

### Remark A.1

Note that the Hamilton–Jacobi action for the theory (121) on the interval with our choice of in/out polarizations is:

$$\begin{aligned} S_\text {HJ}=S^f\big (x(t)=x_\textrm{in},z(t)=z_\textrm{out}\big )\quad = \quad \frac{i}{4}z_\textrm{out}^2-iz_\textrm{out}x_\textrm{in}+\frac{i}{2} x_\textrm{in}^2 . \end{aligned}$$

Here we again recognize the expression in the exponent in (120).

Finally, the measure $e^{-\frac{|z|^2}{2\hbar }}$ in (119) from the BV-BFV standpoint originates from the gluing of intervals (more precisely, from gluing the out-end of one interval with *z*-fixed polarization to the in-end of another interval with $\bar{z}$-fixed polarization). Indeed, consider the theory (121) on the interval $I=[t_0,t_1]$ with some polarization $\mathcal {P}$ at $t_0$ and *z*-fixed polarization at $t_1$, and also the same theory on the interval $I'=[t_1,t_2]$, with some polarization $\mathcal {P}'$ at $t_2$ and with $\bar{z}$-fixed polarization at $t_1$.

The respective actions including the boundary terms adjusting for the polarization are

$$\begin{aligned} S^f_{I}=-\frac{i}{2}\int _{I} \bar{z} dz + f^\mathcal {P}\big |_{t_0},\qquad S^f_{I'}=-\frac{i}{2}\int _{I'} \bar{z} dz -\frac{i}{2} z\bar{z}\big |_{t_1} + f^\mathcal {P'}\big |_{t_2} , \end{aligned}$$

with $f^\mathcal {P},f^{\mathcal {P}'}$ the appropriate^65^ boundary terms at $t_0$, $t_2$. It follows that the partition function for the glued interval $I\cup I'=[t_0,t_2]$ is:

125 $$\begin{aligned} Z_{I\cup I'}(v_\textrm{out}, u_\textrm{in})= & {} \int \mathcal {D}z(t)\,\mathcal {D}\bar{z}(t)\; e^{\frac{i}{\hbar }(S^f_I+\frac{i}{2}z\bar{z}\big |_{t_1}+S^f_{I'})} \nonumber \\= & {} \int _\mathbb {C}\frac{i\, dz\, d\bar{z}}{4\pi \hbar }\; Z_{I'}(v_\textrm{out},\bar{z})\; e^{-\frac{z\bar{z}}{2\hbar }} \; Z_I (z,u_\textrm{in}) . \end{aligned}$$

In the first integral, the term $\frac{i}{2}z\bar{z}\big |_{t_1}$ compensates the boundary term of $S^f_{I'}$ at $t_1$. The final integral is over the values of $z,\bar{z}$ at $t=t_1$. Also, we denoted $u_\textrm{in}$ a coordinate parametrizing the space of leaves of the polarization $\mathcal {P}$ and similarly $v_\textrm{out}$ a coordinate paramterizing the space of leaves of $\mathcal {P}'$. In (125), we see the Segal–Bargmann measure $\frac{i\,dz\,d\bar{z}}{4\pi \hbar }\,e^{-\frac{|z|^2}{2\hbar }}$ , cf. (119), appearing. The normalization factor $\frac{i}{4\pi \hbar }$ is chosen in such a way that, choosing $\mathcal {P}'=\textrm{Span}\{\frac{\partial }{\partial \bar{z}}\}$, we have

$$\begin{aligned} Z_{I\cup I'}(z_\textrm{out},u_\textrm{in})=Z_I(z_\textrm{out},u_\textrm{in}) , \end{aligned}$$

which in turn follows from the identity

$$\begin{aligned} \int \frac{i}{4\pi \hbar } dz\, d\bar{z}\; \underbrace{e^{\frac{z_\textrm{out}\bar{z}}{2\hbar }} }_{Z_{I'}(z_\textrm{out},\bar{z})} e^{-\frac{|z|^2}{2\hbar }}\Psi (z) = \Psi (z_\textrm{out}) \end{aligned}$$

true for any holomorphic function $\Psi (z)$ (for which the l.h.s. converges), applied to $\Psi (z)=Z_I(z,u_\textrm{in})$.

### Aside: contour integration in the complexified space of fields, a lattice toy model

Throughout the paper we are dealing with complexified phase spaces (so that we can impose the convenient holomorphic/antiholomorphic polarizations) and complexified spaces of fields where the path integral should be understood as an integral over a real contour.

A toy model is provided by topological quantum mechanics $S=\int _I pdx$, as above. The phase space for a point is $\Phi =\mathbb {R}^2$. We consider the model with boundary polarization $\textrm{Span}\left\{ \frac{\partial }{\partial z} \right\}$ (i.e., $\bar{z}$ fixed) at $t=0$ and $\textrm{Span}\left\{ \frac{\partial }{\partial \bar{z}} \right\}$ (i.e., *z* fixed) at $t=1$; these polarizations are defined on the complexified phace space $\Phi _\mathbb {C}=\mathbb {C}^2$. The action modified by the appropriate boundary term is $S^f=\int _I (-\frac{i}{2}\bar{z} dz) -\frac{i}{2} z\bar{z}\big |_{t=0}$.

The path integral for this model can be presented by a lattice approximation (which happens to be exact):

126 $$\begin{aligned}{} & {} Z(z_\textrm{out}=z_N,\bar{z}_\textrm{in}=\bar{z}_0)\nonumber \\ {}{} & {} \quad =\int _{C\subset Y_\mathbb {C}} \prod _{k=1}^{N-1} \frac{i\, dz_k d\bar{z}_k}{4\pi \hbar }\;\; e^{ \frac{1}{2\hbar } \big (\sum _{k=1}^N(z_k-z_{k-1})\bar{z}_{k-1}+z_0 \bar{z}_0\big )} \nonumber \\{} & {} \quad = \int _{C\subset Y_\mathbb {C}} \prod _{k=1}^{N-1} \frac{i\, dz_k d\bar{z}_k}{4\pi \hbar }\;\; e^{\frac{1}{2\hbar } \big ( z_1 \bar{z}_0 - z_1 \bar{z}_1 + z_2 \bar{z}_1 - z_2\bar{z}_2 +\cdots - z_{N-1} \bar{z}_{N-1}+ z_N \bar{z}_{N-1} \big )} . \end{aligned}$$

Here:

- We understand that the interval $I=[0,1]$ is partitioned into $N\ge 1$ smaller intervals $[t_0=0,t_1]$, $[t_1,t_2]$, ..., $[t_{N-1},t_N=1]$.
- The space $Y_\mathbb {C}=(\mathbb {C}^2)^{N-1}$—the fiber of the complexified space of fields over boundary conditions—is the product of complexified phase spaces corresponding to $t_1,\ldots ,t_{N-1}$. In particular, we understand that $z_k$ and $\bar{z}_k$ are independent complex variables: they do not have to be complex conjugates of each other.
- The integration is over a “contour” $C\subset Y_\mathbb {C}$—a real $2(N-1)$-dimensional subspace. In particular, the integrand in (126) is a holomorphic $2(N-1)$-form on $Y_\mathbb {C}$ pulled back to *C* by the inclusion.

For the contour *C*, we can consider the following two examples:

(i) Contour $C_1$ given by $\bar{z}_k=z_k^*$, $k=1,\ldots ,N-1$, where $*$ is the complex conjugation.
(ii) Contour $C_2$ given by reality conditions $z_k\in \mathbb {R}\subset \mathbb {C}$, $\bar{z}_k\in i\mathbb {R}\subset \mathbb {C}$.

For $C=C_1$, (126) is an absolutely convergent Gaussian integral (for arbitrary boundary conditions) and yields

$$\begin{aligned} Z(z_\textrm{out}, \bar{z}_\textrm{in}) = e^{ \frac{1}{2\hbar } \bar{z}_\textrm{in}z_\textrm{out}} . \end{aligned}$$

For $C=C_2$, (126) is an oscillatory Fresnel integral which is only conditionally convergent and even that only under special assumptions on the boundary conditions ($\bar{z}_\textrm{in}\in i\mathbb {R}, z_\textrm{out}\in \mathbb {R}$).^66^ When the integral over $C_2$ converges, its value coincides with the result of integration over $C_1$ (which is clear, e.g., from a contour deformation argument).

In summary, we have the complexified space of fields of the lattice theory $F^I_{\mathbb {C}}\ni (\bar{z}_0,z_1,\bar{z}_1,\ldots ,z_{N-1},\bar{z}_{N-1},z_N)$ fibered over the complex space of boundary conditions $\mathcal {B}^{\partial I}_\mathbb {C}\ni \{\bar{z}_\textrm{in},z_\textrm{out}\}$ with complex fiber $Y_\mathbb {C}$ (lattice fields with zero boundary conditions), and the integration in the lattice path integral (126) is over a contour $C\subset Y_\mathbb {C}$—a half-dimensional real submanifold.

## Kodaira–Spencer Theory

We briefly review the definition of the Kodaira–Spencer action functional that was introduced in [8, Section 5], where it was used to analyze the target space physics of the *B*-model. See also [24, Section 2.1].

### Some operations on complex forms

Let *M* be a 6-dimensional Calabi–Yau manifold with a reference holomorphic 3-form $\omega _0$ (sometimes the pair $(M,\omega _0)$ is called a gauged Calabi–Yau manifold). We denote by $\Omega ^{p,q}(M)$ complex forms of Hodge type (*p*, *q*)—sections of the bundle $\wedge ^p (T_\mathbb {C}^*M)^{1,0} \otimes \wedge ^q (T^*_\mathbb {C}M)^{0,1}$ and by $\Omega ^{-p,q}(M)$ sections of the bundle $\wedge ^p (T_\mathbb {C}M)^{1,0} \otimes \wedge ^q(T^*_\mathbb {C}M)^{0,1}$, i.e., (0, *q*)-forms with values in (*p*, 0)-vector fields. Contraction with the reference holomorphic 3-form provides a map

$$\begin{aligned} \Omega ^{-p,q}(M)&\rightarrow \Omega ^{3-p,q}(M) \\ A&\mapsto A^\vee = A\omega _0 \end{aligned}$$

(we omit the symbols for wedge products and contractions). For a (*p*, *q*)-form *A* with $p\ge 0$, we set $A^\vee = A\omega _0^{-1} \in \Omega ^{p-3,q}$, in particular we have $(A^\vee )^\vee = A$. For $A,B,C \in \Omega ^{-1,1}(M)$, we further define the operations

127 $$\begin{aligned} A^\vee \circ B^\vee&= (AB)^\vee = (AB)\omega _0&\in \Omega ^{1,2}(M), \nonumber \\ \langle A^\vee ,B^\vee ,C^\vee \rangle&= A^\vee (B^\vee \circ C^\vee ) = A^\vee (BC)\omega _0&\in \Omega ^{3,3}(M), \nonumber \\ \langle A^3\rangle&= -\frac{1}{6} \frac{\langle A^\vee ,A^\vee ,A^\vee \rangle }{\omega _0\overline{\omega }_0} = \frac{1}{6} (A^3\omega _0)(\overline{\omega }_0)^{-1}&\in \Omega ^{0,0}(M), \end{aligned}$$

and the same operations make sense for $\bar{A},\bar{B}, \bar{C}\in \Omega ^{1,-1}(M)$ if we replace $\omega _0$ by $\bar{\omega }_0$. The minus sign in (127) ensures that $\langle A^3\rangle \overline{\omega }_0 = \frac{1}{6} A^3\omega _0$. Also, in the last equation we made use of the fact that one can divide by sections of a line bundle. By a lemma of Tian [36], if $\partial A = \partial B = 0$, we have

128 $$\begin{aligned}{}[A,B]^\vee = \partial (A^\vee \circ B^\vee ). \end{aligned}$$

### The generating function for Hitchin polarization

For completeness, we include here a derivation of the generating function (113) for the transformation from the linear polarization to the nonlinear polarization. A complex 3-form *A* has decompositions $A = A^{+,\textrm{l}} + A^{-,\textrm{l}}$, with $A^{+,\textrm{l}} \in \Omega ^{3,0}(M) \oplus \Omega ^{2,1}(M)$, and $A^{-,\textrm{l}} \in \Omega ^{1,2}(M) \oplus \Omega ^{0,3}(M)$, and

$$\begin{aligned} A = A^{+,\textrm{nl}} + A^{-,\textrm{nl}} , \end{aligned}$$

where $A^{+,\textrm{nl}}$ and $A^{-,\textrm{nl}}$ are decomposable complex 3-forms. The 3-form *A* is called *nondegenerate* if $A^{+,\textrm{nl}} \wedge A^{-,\textrm{nl}}$ is everywhere nonvanishing. We parametrize

$$\begin{aligned} A^{+,\textrm{nl}}&= \rho e^\mu \omega _0 , \\ A^{-,\textrm{nl}}&= \overline{\rho } e^{\overline{\mu }}\overline{\omega }_0 , \end{aligned}$$

where $$\rho ,\overline{\rho } \in \Omega ^0_\mathbb {C}(M)$$, $$\mu \in \Omega ^{-1,1}(M)$$, $$\overline{\mu }\in \Omega ^{1,-1}(M)$$ and

129 $$\begin{aligned} \rho e^\mu \omega _0 = \rho \left( \omega _0 + \mu \omega _0 + \frac{\mu ^2}{2}\omega _0 + \frac{\mu ^3}{6}\omega _0 \right) . \end{aligned}$$

To write the generating function from the linear to the nonlinear polarization, we use

130 $$\begin{aligned} G(A^{+,\textrm{l}},A^{-,\textrm{nl}}) = \frac{1}{2} \int _M A^{+,\textrm{l}}A^{-,\textrm{l}} - A^{-,\textrm{nl}}A^{+,\textrm{nl}} . \end{aligned}$$

#### Lemma B.1

In the variables $A^{3,0},A^{2,1},\overline{\rho },\overline{\mu }$, the generating function is given by

131 $$\begin{aligned}{} & {} G(A^{3,0},A^{2,1},\overline{\rho },\overline{\mu }) \nonumber \\{} & {} \quad =\int _M \overline{\rho }( A^{3,0}\overline{\omega }_0 + A^{2,1}\overline{\mu }\,\overline{\omega }_0) + \overline{\rho }^2\langle \overline{\mu }^3\rangle \omega _0\overline{\omega }_0 - \frac{\left\langle \left( (A^{2,1} - \frac{1}{2}\overline{\rho }\,\overline{\mu }^2\overline{\omega }_0)^\vee \right) ^3\right\rangle }{(A^{3,0})^\vee - \overline{\rho }\langle \overline{\mu }^3\rangle }\omega _0\overline{\omega }_0 . \nonumber \\ \end{aligned}$$

#### Proof

This is a tedious but straightforward computation. One way to do it is to express *G* in terms of $\rho ,\mu ,\overline{\rho },\overline{\mu }$ first. To this end, notice that the decomposition in (129) is a decomposition into forms of definite Hodge type. Thus, we can write

132 $$\begin{aligned} A^{3,0}&= {\rho }\,{\omega }_0+ \overline{\rho }\frac{\overline{\mu }^3}{6} \overline{\omega }_0 = \left( \rho + \overline{\rho }\langle \overline{\mu }^3\rangle \right) \omega _0, \end{aligned}$$

133 $$\begin{aligned} A^{2,1}&={\rho }\,{\mu }\,{\omega }_0 + \overline{\rho }\frac{\overline{\mu }^2}{2}\overline{\omega }_0, \end{aligned}$$

and similarly for $A^{0,3}$ and $A^{1,2}$. We thus obtain

134 $$\begin{aligned} A^{3,0}A^{0,3}&= \rho \overline{\rho }(1+ \langle \mu ^3\rangle \langle \overline{\mu }^3\rangle )\omega _0\overline{\omega }_0 + \left( \rho ^2\langle \mu ^3\rangle \ + \overline{\rho }^2\langle \overline{\mu }^3\rangle \right) \omega _0\overline{\omega }_0, \end{aligned}$$

135 $$\begin{aligned} A^{2,1}A^{1,2}&= \rho \overline{\rho }\left( \mu \omega _0\overline{\mu }\,\overline{\omega }_0 + \frac{1}{4}\overline{\mu }^2\overline{\omega }_0\mu ^2\omega _0\right) +\frac{1}{2}\left( \rho ^2\mu \omega _0{\mu }^2\omega _0 - \overline{\rho }^2\overline{\mu }\,\overline{\omega }_0\overline{\mu }^2\overline{\omega }_0\right) . \end{aligned}$$

On the other hand, we have

136 $$\begin{aligned} A^{-,\textrm{nl}}A^{+,\textrm{nl}} = \rho \overline{\rho }\left( \overline{\omega }_0\omega _0 + \overline{\mu }\,\overline{\omega }_0\mu \omega _0 + \frac{1}{4}\overline{\mu }^2\overline{\omega }_0\mu ^2\omega _0 + \langle {\overline{\mu }^3}\rangle \langle \mu ^3\rangle \omega _0\overline{\omega }_0\right) . \end{aligned}$$

Summing (134) and (135) and subtracting (136), the last two terms in (136) cancel and we obtain

137 $$\begin{aligned} A^{+,\textrm{l}}A^{-,\textrm{l}} - A^{-,\textrm{nl}}A^{+,\textrm{nl}}= & {} 2\rho \overline{\rho }\left( \omega _0\overline{\omega }_0 -\overline{\mu }\,\overline{\omega }_0\mu \omega _0\right) \nonumber \\{} & {} +\, \rho ^2\left( \langle \mu ^3\rangle \omega _0\overline{\omega }_0 + \frac{1}{2} \mu \omega _0 \mu ^2\omega _0\right) \nonumber \\{} & {} +\overline{\rho }^2\left( \langle \overline{\mu }^3\rangle \omega _0\overline{\omega }_0-\frac{1}{2}\overline{\mu }\,\overline{\omega }_0\overline{\mu }^2\overline{\omega }_0\right) . \end{aligned}$$

Recall that $\mu \omega _0 \mu ^2\omega _0 = (\mu ^3\omega _0)\omega _0 = 6\langle \mu ^3\rangle \overline{\omega }_0\omega _0$, hence we can simplify this expression to

$$\begin{aligned} A^{+,\textrm{l}}A^{-,\textrm{l}} - A^{-,\textrm{nl}}A^{+,\textrm{nl}} = 2\rho \overline{\rho }\left( \omega _0\overline{\omega }_0 -\overline{\mu }\,\overline{\omega }_0\mu \omega _0\right) -2 \left( \rho ^2\langle \mu ^3\rangle +\overline{\rho }^2\langle \overline{\mu }^3\rangle \right) \omega _0\overline{\omega }_0. \end{aligned}$$

From equations (132), (133) we get

$$\begin{aligned} \rho \omega&= A^{3,0} - \overline{\rho }\langle \overline{\mu }^3\rangle \omega _0, \\ \rho \mu \omega _0&= A^{2,1} - \frac{1}{2}\overline{\rho }\, \overline{\mu }^2\overline{\omega }_0, \end{aligned}$$

which we use to rewrite the first term as

$$\begin{aligned} 2\rho \overline{\rho }\left( \omega _0\overline{\omega }_0 -\overline{\mu }\,\overline{\omega }_0\mu \omega _0\right) =2\overline{\rho }A^{3,0}\overline{\omega }_0 - 2\overline{\rho }^2\langle \overline{\mu }^3\rangle \omega _0 + 2\overline{\rho }A^{2,1}\mu \omega _0 + 6\overline{\rho }^2\langle \overline{\mu }^3\rangle \omega _0\overline{\omega }_0. \end{aligned}$$

In total, (130) evaluates to

138 $$\begin{aligned} G = \int _M \overline{\rho }A^{3,0}\overline{\omega }_0 + \overline{\rho }A^{2,1}\overline{\mu }\,\overline{\omega }_0 + \overline{\rho }^2\langle \overline{\mu }^3\rangle \omega _0\overline{\omega }_0 - \frac{\langle (\rho \mu )^3\rangle }{\rho }\omega _0\overline{\omega }_0. \end{aligned}$$

Formula (131) may now be obtained by solving equations (132), (133) for $\rho$ and $\rho \mu$, which gives

139 $$\begin{aligned} \rho&= \frac{A^{3,0} - \frac{1}{6}\overline{\rho }\,\overline{\mu }^3\overline{\omega }_0}{\omega _0} = (A^{3,0})^\vee - \overline{\rho }\langle \overline{\mu }^3\rangle , \end{aligned}$$

140 $$\begin{aligned} \rho \mu&= \frac{A^{2,1} - \frac{1}{2}\overline{\rho }\,\overline{\mu }^2\overline{\omega }_0}{\omega _0} = \left( A^{2,1} - \frac{1}{2}\overline{\rho }\,\overline{\mu }^2\overline{\omega }_0\right) ^\vee . \end{aligned}$$

Plugging (139), (140) into (138) we obtain (131). $\square$

The defining property of *G* is the following.

#### Lemma B.2

We have $\delta G = \theta ^\textrm{l} - \theta ^\textrm{nl}$ where $\theta ^\textrm{l} = A^{-,\textrm{l}}\delta A^{+,\textrm{l}}$ and $\theta ^\textrm{nl}= A^{+,\textrm{nl}}\delta A^{-,\textrm{nl}}$.

#### Proof

This follows from Eq. (130). But one can also check it through direct computation: we have

$$\begin{aligned} \frac{\delta G}{\delta A^{3,0}} = \overline{\rho }\,\overline{\omega }_0 + \frac{\left\langle \left( \left( A^{2,1} -\frac{1}{2}\overline{\rho }\,\overline{\mu }^2\overline{\omega }\right) ^\vee \right) ^3\right\rangle }{(A^{3,0})^\vee - \overline{\rho }\langle \overline{\mu }^3\rangle ^2}(\omega _0)^{-1}\omega _0\overline{\omega }_0 = \overline{\rho }\,\overline{\omega }_0+ \frac{\langle (\rho \mu )^3\rangle }{\rho ^2}\overline{\omega }_0 = A^{3,0}. \end{aligned}$$

Notice that we have

$$\begin{aligned} \frac{\delta }{\delta \mu }\langle \mu ^3\rangle = \frac{1}{2} (\mu ^2\omega )(\overline{\omega })^{-1}. \end{aligned}$$

It follows that

$$\begin{aligned} \frac{\delta }{\delta A^{2,1}} \langle ((A^{2,1})^\vee )^3\rangle = \frac{1}{2} (((A^{2,1})^\vee )^2\omega _0)\overline{\omega }_0^{-1}\omega _0^{-1} = -\frac{\frac{1}{2}(((A^{2,1})^\vee )^2\omega _0)}{\omega _0\overline{\omega }_0^{-1}} \end{aligned}$$

(note the sign) and therefore

$$\begin{aligned} \frac{\delta G}{\delta A^{2,1}} = \overline{\rho }\,\overline{\mu }\,\overline{\omega }+ \frac{1}{2}\rho \mu ^2\omega _0 = A^{1,2}. \end{aligned}$$

This proves that $\delta G/\delta A^{+,\textrm{l}} = A^{-,\textrm{l}}$. Computing $\delta G /\delta \overline{\rho }$ gives

$$\begin{aligned} \frac{\delta G}{\delta \bar{\rho }}\delta \overline{\rho }&= \delta \overline{\rho }\left( A^{3,0}\overline{\omega }_0 + A^{2,1}\overline{\mu }\,\overline{\omega }_0 + 2\overline{\rho }\langle \overline{\mu }^3\rangle \omega _0\overline{\omega }_0 + \frac{1}{2}\rho \mu ^2\omega _0(\frac{1}{2}\overline{\mu }^2\overline{\omega }_0) + \rho \langle \mu ^3\rangle \omega _0\langle \overline{\mu }^3\rangle \overline{\omega }_0\right) \\&= -\rho e^\mu \omega _0e^{\overline{\mu }}\overline{\omega }_0\delta \overline{\rho }. \end{aligned}$$

Finally, computing $\delta G/\delta \overline{\mu }$ gives

$$\begin{aligned} \frac{\delta G}{\delta \overline{\mu }}\delta \overline{\mu }&= - \overline{\rho }A^{2,1}(\delta \overline{\mu }\, \overline{\omega }_0) + \frac{1}{2}\overline{\rho }^2(\overline{\mu }^2\overline{\omega }_0)(\delta \overline{\mu }\,\overline{\omega }_0) + \frac{1}{2}\rho \mu ^2\omega _0(\overline{\rho }\,\overline{\mu }\,\delta \overline{\mu }\,\overline{\omega }_0)\\ {}&\quad + \rho \langle \mu ^3\rangle (\delta \overline{\mu }\,\overline{\omega }_0)\frac{1}{2}\overline{\rho }\,\overline{\mu }^2\overline{\omega }_0 \\&= -\overline{\rho }\rho \,\mu \,\omega _0 (\delta \overline{\mu }\,\overline{\omega }_0) + \frac{1}{2}\rho \mu ^2\omega _0(\overline{\rho }\,\overline{\mu }\,\delta \overline{\mu }\,\overline{\omega }_0) + \rho \langle \mu ^3\rangle (\delta \overline{\mu }\,\overline{\omega }_0)\frac{1}{2}\overline{\rho }\,\overline{\mu }^2\overline{\omega }_0 \\&= -\rho \overline{\rho }(e^\mu \omega _0)\delta \overline{\mu }(\overline{\omega }_0 + \overline{\mu }\omega _0 + \frac{1}{2}\overline{\mu }^2\overline{\omega }_0). \end{aligned}$$

Using

$$\begin{aligned} \delta A^{-,\textrm{nl}} = \delta \overline{\rho }e^{\overline{\mu }}\overline{\omega }_0 = e^{\overline{\mu }}\overline{\omega }_0\delta \overline{\rho }+ \overline{\rho }\delta \overline{\mu }(\overline{\omega }_0 + \overline{\mu }\omega _0 + \frac{1}{2}\overline{\mu }^2\overline{\omega }_0), \end{aligned}$$

we obtain

$$\begin{aligned} \delta G = A^{+,\textrm{l}}\delta A^{-,\textrm{l}} - A^{+,\textrm{nl}}\delta A^{-,\textrm{nl}}. \end{aligned}$$

$$\square $$

### Deformations of complex structures

Let *M* be a compact Calabi–Yau manifold supplied with a reference holomorphic 3-form $\omega _0$. A deformation of the complex structure is equivalent to a deformation of the $\bar{\partial }$ operator $\bar{\partial }\rightarrow \bar{\partial }_{\bar{A}} = \bar{\partial }+ \bar{A}$, where $\bar{A} \in \Omega ^{-1,1}(M) = \Gamma (T^{1,0}M \otimes (T^*)^{0,1}M)$. The integrability condition $\bar{\partial }_A^2 = 0$ is equivalent to [29]

141 $$\begin{aligned} \bar{\partial }\bar{A} + \frac{1}{2} [\bar{A},\bar{A}] = 0. \end{aligned}$$

The moduli space of complex structures is thus given by solutions of (141) modulo gauge transformations

142 $$\begin{aligned} \delta \bar{A} = \bar{\partial }\varepsilon + [\bar{A},\varepsilon ], \end{aligned}$$

with $\varepsilon \in \Omega ^{-1,0}(M)$. The tangent space to the moduli space of complex structures is given by the linearization of (141), i.e., it is the quotient of $\{\alpha :\bar{\partial }\bar{\alpha } = 0\}$ by linearized gauge transformations $\delta \alpha = \bar{\partial }\varepsilon$.

After Tian ([36]), this problem can be reformulated using $\bar{A}^\vee$ as follows. Imposing the constraint $\partial \bar{A}^\vee =0$ and using (128), we can rewrite (141) as

143 $$\begin{aligned} \bar{\partial }\bar{A}^\vee + \partial (\bar{A}^\vee \circ \bar{A}^\vee ) = 0. \end{aligned}$$

### Kodaira–Spencer action

The Kodaira–Spencer action functional as introduced in [8] is

144 $$\begin{aligned} S_{KS}[\bar{A}^\vee ]= \int _M\frac{1}{2} \bar{A}^\vee \partial ^{-1}\bar{\partial }\bar{A}^\vee + \frac{1}{6} \langle \bar{A}^\vee ,\bar{A}^\vee ,\bar{A}^\vee \rangle . \end{aligned}$$

Here the first term is well-defined due to $\partial \bar{\partial }$-lemma. The equation of motion of (144) is (143). One can resolve the nonlocality by writing $\bar{A}^\vee = x + \partial b$, where *x* is a $\partial$-harmonic (2, 1)-form. The action functional then becomes

145 $$\begin{aligned} S_{KS}(x;b) = \int _M \frac{1}{2} \partial b\bar{\partial }b + \frac{1}{6} \langle (x + \partial b),(x + \partial b),(x + \partial b)\rangle . \end{aligned}$$

This action functional has the following remarkable property. From Eq. (143), it follows that any harmonic (2, 1)-form $x = \bar{A}^\vee _1$ can be interpreted as a first order deformation of the complex structure. The tree level diagrams of (145) then generate forms $\bar{A}^\vee _n$ with the property that $\bar{A}^\vee = \sum \varepsilon ^n \bar{A}^\vee _n$ is a solution of the Kodaira–Spencer equation (143). We refer to Bershadsky et al. [8, Section 5.2] for details.

## Footnotes

1 In our framework, this is due to the fact that the constraints are affine functions of the variables describing the field configurations on either boundaries (such theories are called “biaffine” in [14]).

2 The symplectic form is odd because it pairs components of $\mathcal {A}$,$\mathcal {A}'$ of opposite parity, which in turn is due to the fact that $\dim N$ is odd.

3 BFV is short for Batalin–Fradkin–Vilkovisky [7, 18].

4 The vector field $Q_N$ is no longer the hamiltonian vector field of $\mathcal {S}_N$. It is instead defined via the formula above.

5 A comment on complex vs. real spaces: by default, spaces of fields and spaces of boundary fields are real vector spaces. In this paper, we have to complexify them to impose convenient boundary conditions/polarizations. The splittings involved in the gauge fixing (and thus in the corresponding spaces $\mathcal {Y}$, $\mathcal {V}$, $\mathcal {Y}'$, $\mathcal {L}$) are only defined over $\mathbb {C}$. The equations of motion and the evolution relation are defined over $\mathbb {R}$ but need to be complexified to be described by a generating function (involving a complex polarization). When writing down path integrals an implied step is a choice of a real contour in the complexified space of fields, see Appendix A.1 for an illustration of the principle.

6 In general, one might have to restrict to neighborhoods in $\mathcal {F}^\partial _{\partial N}$ and $\mathcal {B}$ to achieve this isomorphism.

7 Since we are inverting only the de Rham differential along the interval $d_I$, this is the unique possible choice of gauge-fixing lagrangian. Invertibility of $d_I$ restricted to $\mathcal {L}$ requires that $\mathcal {L}$ contains no 1-forms. The lagrangian condition then requires that all 0-forms along *I* in $\mathcal {Y}$ belong to $\mathcal {L}$.

8 As a matter of fact, they even depend on the *Riemanian metric* inducing the complex structure on the boundary, a phenomenon known as conformal anomaly. See Remark 4.3.(b).

9 Such an action functional is the classical part of an AKSZ theory [3] on an interval *I*. See also [6] for the study of such a theory in the Dirac formalism.

10 In particular, this implies that *L* is lagrangian, i.e., that the symplectic orthogonal of $T_vL$ is $T_vL$ itself for every point *v* of *L*.

11 In the case of 3D Chern–Simons theory, Example 2.1, *C* is the space of flat connections on the surface $\Sigma$, and the reduced phase space is the space of flat connections modulo gauge transformations.

12 More precisely, one looks for solutions of the EL equations that are critical points for the action. This requires changing the boundary one-form by an exact term in such a way that it vanishes on $L_0$ and $L_1$. In particular, this can only happen if $L_0$ and $L_1$ are isotropic. Moreover, we want the intersection of $L_0\times L_1$ with *L* to be discrete, so that locally the solution is unique. At each intersection point, the tangent spaces to $L_0\times L_1$ and to *L* are then complementary, which implies that they are not only isotropic but split lagrangian. We want this to happen for generic boundary conditions. This is the reason why *L* is required to be split lagrangian.

13 In the examples of this paper, *B* is a vector space, so $T^*B$ is of the form $B^*\oplus B$. We are also interested in complex polarizations. In the case at hand, this simply means allowing *B* to be a complex vector space. Then the complexified phase space is $B^*\oplus B$.

14 This polarization is known in the literature on Chern–Simons theory. In a context close to the context of the present paper, it was discussed in [12, Section 2.4.4] and in [1].

15 Here we are using the conventions of Sect. 5.3 (Lemma 5.14) which are different from the conventions of [14].

16 This is the abelian version of the theory considered in [2].

17 It is nonzero in nonabelian theory: there one has $S_{pt^\pm }=\pm \frac{1}{6} (\psi ,[\psi ,\psi ])$.

18 See [13] and [14, Section 9] for the details on discontinuous extension of boundary fields.

19 Note that here we have chosen $\mathcal {V}$ to be larger than cohomology (which in fact vanishes in this case).

20 Depending on the choice of boundary conditions (e.g., in the case of Sect. 4.2), the space of forms subject to boundary conditions $\mathcal {Y}[-1]$ may fail to be a subcomplex of $\Omega ^\bullet (I\times \Sigma )$ with respect to the total de Rham differential $d_I+d_\Sigma$. However, the operator *K* we are constructing can be seen as a chain contraction for just the “axial” differential $d_I$.

21 The integral over $\Sigma$ in (26) is understood as being with respect to the “standard” orientation, which coincides with the induced one from the cylinder on the out-boundary and is opposite to the induced one on the in-boundary.

22 One can see $S_\text {HJ}$ as the abelian version of the gauged Wess–Zumino–Witten theory, see e.g (2.7) in [22].

23 We have to split off the constants from $\sigma$, because they are in the kernel of the Laplacian $\partial \bar{\partial }$ and thus would obstruct the evaluation of the integral of (33) over $\sigma$.

24 Recall that, in terms of Dolbeault operators and the area form, the metric Laplace operator is: $\Delta _g=\frac{2i}{\mu }\partial \bar{\partial }$.

25 I.e., with no auxiliary fields.

26 This is the quantization of the hamiltonian $H = \textsf{A}^2_{\textrm{res},c}\int _\Sigma \underline{\sigma }\dot{\mu }$ generating the family of lagrangians given by $\mu _{g^\tau }$, see [13, Section 2].

27 We are referring to the fact that in a conformal field theory with central charge *c*, the partition function has the following behavior under Weyl transformations of metric: $Z_\textrm{CFT}^{e^{\phi }g} =Z_\textrm{CFT}^{g} \cdot e^{\frac{c}{48\pi }\int _\Sigma \frac{1}{2} d\phi \wedge * d\phi +R\phi \mu _g}$, see, e.g., [20].

28 Here the factor 2 comes from the fact that the odd symplectic form on $\mathcal {V}$, induced from the standard BV 2-form on the space of fields, $\omega _\mathcal {F}=\int _{ I\times \Sigma }\frac{1}{2} \delta \mathcal {A}\,\delta \mathcal {A}$, is $\omega _\mathcal {V}=\frac{1}{2} \int _\Sigma \delta \textsf{A}^+_\textrm{res}\, \delta \textsf{A}^-_{I\,\textrm{res}}+ \delta \textsf{A}^-_\textrm{res}\, \delta \textsf{A}^+_{I\,\textrm{res}}$. The factor $\frac{1}{2}$ in the latter expression comes from $\int _0^1 dt (1-t)=\int _0^1 dt\, t=\frac{1}{2}$.

29 The prime example being $\mathfrak {g}= \Omega ^\bullet (M)$ with *M* a 2*k*-manifold, $d_\mathfrak {g}$ the de Rham differential and $(\alpha ,\beta ) = \int _M \alpha \wedge \beta$.

30 If $\{t_a\}$ is a basis in $\mathfrak {g}$ and $\{\psi ^a\}$ is the shifted dual basis in $X^*$, then we have $\psi =\sum _a t_a \psi ^a \in \textrm{Hom}(X,\mathfrak {g})$. If the degree of $t_a$ is |*a*|, then the ghost number of $\psi ^a$ is $k-|a|$, and thus $\psi$ is indeed an object of total degree *k*.

31 I.e., coordinates, e.g., on $X^{[>0]}$ have positive ghost number so $X^{[>0]} = \bigoplus _{i<k}\mathfrak {g}^i[k]$.

32 A particularly important example is the case $\mathfrak {g}= \Omega ^\bullet (M)$ where *M* is a 2*k*-manifold, with $k=2l+1$ odd, endowed with a complex structure that allows us to decompose $\Omega ^k_\mathbb {C}(M) = \Omega ^+(M) \oplus \Omega ^-(M)$ into lagrangian subspaces with respect to $(\alpha ,\beta )=\int _M \alpha \wedge \beta$, with the splitting given by (106) below. In the case $k=1$, we have $d^+_\mathfrak {g}= \partial ,d^-_\mathfrak {g}= \bar{\partial }$.

33 Since $\psi$ is odd, we have, e.g., $\delta (\psi ^+,\psi ^-) = (\psi ^-,\delta \psi ^+) - (\psi ^+,\delta \psi ^-)$.

34 We understand $A^{[k]}_\textrm{res}$ as valued in $X^{[k]}$ but with ghost number $k-1$; the shift is due to the fact that $A^{[k]}_\textrm{res}$ is a coefficient of a 1-form on the source. This shift is a standard feature of the AKSZ construction. In particular, $A^{[1]}_\textrm{res}$ is an object of ghost number *zero*.

35 We are ignoring here the ghost vertices that in this case do not contribute to the effective action since no vertex carries as $\psi ^{[>0]}$.

36 I.e., the function $G(\psi ^-,\psi ^Q)$ satisfying $\delta G = \psi ^+ \delta \psi ^- - \psi ^P \delta \psi ^Q$.

37 Strictly speaking, the range of $\Phi$ is not $\mathcal {F}$, but rather a certain regularization of $\mathcal {F}$ more suitable for quantization. See the discussion in [14, Section 9.2.3].

38 There are no bulk vertices in this polarization. The two contributing terms come from multivalent $\textrm{out}$-boundary vertices in ghost number 0 and univalent boundary vertices in the ghost sector.

39 To simplify the notations, and to be able to write expressions like $g^{-1}\partial g$ below, we will assume that *G* is a matrix group. Otherwise, we should use left/right translations in *G*.

40 Curiously, the issue of convergence arises only in diagrams (v), (vi), (viii)—the diagrams involving ghosts.

41 By the standard result on Wess–Zumino terms, $\textrm{WZW}(g) \bmod 4\pi ^2 \mathbb {Z}$ is independent of the choice of $\widetilde{g}$ interpolating between *g* on one end of the cylinder and 1 on the other.

42 Recall, see [35], that on an (*n*|*n*)-dimensional odd symplectic supermanifold $(\mathcal {M},\omega )$, half-densities can be understood as cohomology classes of the differential $\omega \wedge$ acting on differential forms $\Omega ^\bullet (\mathcal {M})$. Moreover, if $(x^i,\xi _i)$ are Darboux coordinates, $i=1,\ldots ,n$, with $x^i$ the even coordinates, then each cohomology class has a unique representative of the form $\rho (x,\xi ) dx^1\cdots dx^n \in \Omega ^n(\mathcal {M})$ corresponding to the half-density $\rho (x,\xi )\prod _i d^{\frac{1}{2}} x D^{\frac{1}{2}}\xi$, with $\rho$ some function. In (67), “$\sim$” refers this choice of preferred representative for a half-density.

43 The superscript (0) means the “0-loop part,” corresponding to the expansion in powers of $\hbar$: $S^\textrm{eff}=\sum _{k=0}^\infty (-i\hbar )^k S^{\textrm{eff}\,(k)}$. In the present case, we have only $k=0,1$ terms.

44 The notation $Y=-\frac{\delta \mathbb {I}}{\delta g\cdot g^{-1}}$ means that variation of $\mathbb {I}$ under the variation of *g* is $\delta _g \mathbb {I}=-\int _\Sigma \langle \delta g\cdot g^{-1}, Y \rangle$.

45 The BFV operator (81), its generalization to the case of Wilson lines intersecting the boundary—see (95) below—and its cohomology in genus zero were discussed in [1].

46 Our convention is that the partition function is not a homomorphism from in-states to out-states (depending on residual fields), but an element of $\mathcal {H}_\textrm{out}\widehat{\otimes }\mathcal {H}_\textrm{in}$, i.e., without dualization of the in-factor. In this description, gluing of two cylinders involves a pairing between the out-states of the first cylinder $\mathcal {H}_\textrm{out}^I$ and the in-states $\mathcal {H}_\textrm{in}^{II}$ of the second.

47 See [1] for a construction of these BFV operators from the presentation of Wilson lines via a path integral over auxiliary fields supported on $\gamma _j$ (the Alekseev–Faddeev–Shatashvili formula).

48 Up to the subtleties concerning ghost loops discussed above.

49 Where Witten suggests that “this in fact can be regarded as the essential relation between the WZW model and Chern–Simons theory.”

50 In our approach, holomorphicity simply follows from the fact that it depends only on $\textsf{A}^{1,0}_\textrm{out}$, while gauge invariance is the statement that $\Omega _\textrm{out}\psi = 0$.

51 A missing factor of $\int _\Sigma A^{1,0}A^{0,1}$ in comparison with [38] comes from the gluing conventions in BV-BFV, see Appendix A.

52 See, for instance, Gawedzki and Kupiainen [23] for a more detailed discussion in genus 0, or [17] for a discussion in genus 1. The authors do not know of an explicit construction of this inner product in higher genera.

53 The gluing is defined as a formal functional integral, but its restriction to the finite-dimensional $\Omega$-cohomology gives rise to a well-defined inner product.

54 $\Lambda ,\Lambda ^*$ are $\mathcal {G}$-valued forms while $g^*$ is a $\mathcal {G}$-valued form translated by $g^{-1}$. The ghost number is 0 for $g,\Lambda$ and is $-1$ for $g^*,\Lambda ^*$.

55 There is, of course, a similar change of variables where instead we take the (0, 1)-component at $t=0$ to be a coordinate on $\mathcal {V}$. It leads to an expression for $S^\textrm{eff}$ where the symmetry between in/out boundaries is broken in the opposite way to (105): the coefficients of $\textsf{A}^{1,0}_\textrm{in}$, $c_\textrm{out}$ are simple and the coefficients of $\textsf{A}^{1,0}_\textrm{out}$, $c_\textrm{in}$ are more complicated.

56 There are other ways to get at this results. For example, we may consider the classical theory with action $S_\text {cl}=\int _{ I\times M} \frac{1}{2} AdA$ with *A* a *k*-form for some *k*. This immediately forces $d=2k+1$. For *k* even, we have $AdA=\frac{1}{2}dA^2$, so the classical action has no bulk term. We then have to assume *k* odd. Another option is to consider the AKSZ construction with target $\mathbb {R}[k]$, for some *k*, endowed with a symplectic form. If we denote by *x* the coordinate, the general 2-form is $\omega =f(x)dxdx$ with *f* a function. If *k* is even, $dxdx=0$ and $\omega$ is degenerate, so we have to assume *k* odd. In this case $f(x)=a+bx$ for some real numbers *a* and *b*. Now $\omega$ is closed iff $b=0$ and nondegenerate iff $a\not =0$ (we may, e.g., take $a=\frac{1}{2}$). We then have that $\omega$ has degree 2*k*. Since we want to produce a BV form (i.e., degree equal to $-1$) on $I\times M$ by the AKSZ construction, we need $d=2k+1$.

57 In more detail, for a (*p*, *q*)-form $\alpha$ with $p+q = k-1$ we have

$$\begin{aligned} d_M^+\alpha ={\left\{ \begin{array}{ll} d_M\alpha , &{} q<l, \\ \partial \alpha , &{} q=l, \\ 0, &{} q > l. \end{array}\right. } \end{aligned}$$

and vice versa for $d_M^-$.

58 We use freely the notation from Sect. 5.1.

59 For consistency with [24] and [8], we switch here to the polarization with base parametrized by $\textsf{A}^{3,+,\textrm{l}}$, as opposed to the rest of the paper.

60 See Appendix A for a motivation for the comparison of BV-BFV partition function with standard Segal–Bargmann transform.

61 This state $\psi _\textrm{ph}$ was proposed in [24] as a way of “fixing the string coupling constant.” It is a quantization of the lagrangian given by $\overline{\mu }=0$, $p_{\overline{\rho }}=1$, where $p_{\overline{\rho }}$ denotes the canonical momentum of $\overline{\rho }$.

62 This is just the identity $x \delta (x) = 0$.

63 We can choose $\lambda$ such that $\partial ^*\lambda = 0$, so that $b \in \mathcal {L}\cap \Omega ^{1,1}(M)$.

64 To give a rigorous proof of this statement would require to give a strict interpretation of the functional integral in (118), which is beyond the scope of the present paper. It should be noted that the restriction of *b* to the subspace $\partial ^*b = 0$ is a gauge-fixing condition for the KS theory, so one should consider also the the gauge independence of $Z_{KS}$.
